# Metabolism and transcriptome profiling provides insight into the genes and transcription factors involved in monoterpene biosynthesis of borneol chemotype of *Cinnamomum camphora* induced by mechanical damage

**DOI:** 10.7717/peerj.11465

**Published:** 2021-07-01

**Authors:** Zerui Yang, Chunzhu Xie, Yuying Huang, Wenli An, Shanshan Liu, Song Huang, Xiasheng Zheng

**Affiliations:** School of Pharmacy, Guangzhou University of Chinese Medicine, Guangzhou, Guangdong, China

**Keywords:** Borneol chemotype of Cinnamomum camphora, Metabolism, Transcriptome, Monoterpene biosynthesis, Transcription factor

## Abstract

**Background:**

The borneol chemotype of *Cinnamomum camphora* (*BCC*), a monoterpene-rich woody plant species, is the sole source prescribed by the Chinese Pharmacopoeia for the production of natural D-borneol, a major monoterpene in *BCC* used for millennia as a topical analgesic in China. Nevertheless, the possible gene-regulatory roles of transcription factors (TFs) in *BCC*’s monoterpenoid biosynthesis remained unknown. Here, a joint analysis of the transcriptome and terpenoid metabolome of *BCC* induced by mechanical damage (MD) was used to comprehensively explore the interaction between TFs and terpene synthase (TPS) unigenes that might participate in monoterpene biosynthesis in* BCC*.

**Results:**

Gas chromatography–mass spectrometry analysis detected 14 monoterpenes and seven sesquiterpenes. All but two monoterpenes underwent a significantly increased accumulation after the MD treatment. RNA sequencing data revealed that 10 TPS, 82 MYB, 70 AP2/ERF, 38 BHLH, 31 WRKY, and 29 bZIP unigenes responded to the MD treatment. A correlation analysis revealed that three monoterpene synthase genes (CcTPS1, CcTPS3, CcTPS4) highly correlated with multiple monoterpenes, namely D-borneol, camphor, and bornyl acetate, which could be responsible for monoterpenoid biosynthesis in *BCC*. Furthermore, five WRKY, 15 MYB, 10 ERF/AP2, five bZIP, and two BHLH genes had strong, positive correlations with *CcTPS1* or *CcTPS4*, judging by their high coefficient values (R^2^ > 0.8). The bioinformatics results were verified by quantitative real-time PCR.

**Conclusion:**

This study provides insight into the genes involved in the biosynthesis and regulation of monoterpene in *BCC* and thus provides a pool of candidate genes for future mechanistic analyses of how monoterpenes accumulate in *BCC*.

## Introduction

*Cinnamomum camphora*, a broad-leaved evergreen plant species in the Lauraceae family, is a precious timber and aromatic tree that has been cultivated in China for at least 2000 years (Nirmal Babu et al., 2003). All plant parts of *C. camphora* contain aromatic oils, especially in its leaves. The essential oil of *C. camphora* leaves is rich in monoterpenes and sesquiterpenes, but this oil’s main constituents display significant phytochemical intraspecific variation. According to the proportion of main components in the essential oil extracted from *C. camphora* leaves, at least five chemotypes are known to exist (Guo et al., 2017), of which the borneol chemotype of *C. camphora* (hereon *BCC*) is the only source of natural D-borneol prescribed by the Chinese Pharmacopoeia (Pharmacopoeia, 2015).

According to the Chinese Pharmacopoeia, natural D-borneol is effective for relieving pain and fever as well as inducing resuscitation, and it can be used for treating strokes and many other cardiovascular diseases, for which support can be found in modern pharmacological and clinical studies (Pharmacopoeia, 2015). It was isolated from leaves of the *BCC* tree and historically used as a topical analgesic for millennia (Wang et al., 2017). Nevertheless, the current supply of D-borneol is limited because the strong demand exceeds its poor production yields (Xie et al., 2019; Yu et al., 2019). Besides D-borneol, the essential oil of *BCC* leaves also contains various monoterpenes, such as camphor, eucalyptol, limonene, and pinene, among others. Camphor is an antimicrobial agent able to inhibit the growth of bacteria and fungi (Tabanca et al., 2001); it is also commonly used in household sanitary products. A popular volatile ingredient, eucalyptol is widely used in many essential oils to relieve sinus and lung congestion caused by various conditions and ailments (Goto et al., 2020; Kennedy-Feitosa et al., 2019). More recently, limonene was shown to exert neuroprotective effects against A *β*42-induced cytotoxicity (Shin et al., 2020). In sum, the monoterpenes in the *BCC*’s essential oil are of considerable research interest due to their potential biological activity.

Two independent biosynthetic pathways, namely the cytosolic mevalonic acid (MVA) pathway and the plastic 2-C-methyl-D-erythritol-4-phosphate (MEP) pathway, are thought to figure prominently in the biosynthesis of plant terpenoids (Yin et al., 2017). Most monoterpenes are derived from the geranyl diphosphate (GPP) in the plastic MEP pathway, and then catalyzed by monoterpene synthases to form various monoterpenes (Adal et al., 2019; Mahmoud & Croteau, 2002; Rabiei, Bahador & Kordrostami, 2018). Although the molecular mechanisms of certain monoterpenes dominant in *C. camphora* oil are unknown, studies did show that the expression of many monoterpenoid synthases coding genes are mainly regulated at the level of transcription (Adal et al., 2017; Adal et al., 2019; Sarker & Mahmoud, 2015). Transcription factors (TFs) generally play an important role in plant growth, evolution, metabolism, and responses to abiotic and biotic stress factors by regulating the expression of related genes (He et al., 2018; Zhao et al., 2020). Recent research has shown that MYB (myeloblastosis related)-, bHLH (basic helix-loop-helix)-, ERF (APETALA2/ethylene responsive factor)-, WRKY- and bZIP (basic leucine zipper)-type TFs can regulate the expression of plant TPS genes by binding to their promoter. For example, SlWRKY73 can transiently transactivate several *Solanum lycopersicum* terpene synthase genes’ promoters, including *SlTPS5, SlTPS3*, and *SlTPS7*, in *Nicotiana benthamiana* (Spyropoulou, Haring & Schuurink, 2014). In other work, a bZIP transcription factor HY5 isolated from *Artemisia annua* is capable of binding to the promoter region of a *β*-pinene synthase gene QH6 to modulate the latter’s rhythmic expression (Zhou et al., 2015). In *Solanum lycopersicum,* SlMYC1 is a basic helix-loop-helix TF that functions as a positive regulator of monoterpene biosynthesis, in both leaf and stem trichomes, while it acts as a negative regulator of sesquiterpene biosynthesis in stem trichomes (Xu & van Herwijnen, 2018). Among the cloned members of the AP2/ERF transcription factor gene family from *Citrus sinensis* Osbeck, *CitERF71* has a similar expression pattern to *CitTPS16* and directly binds to ACCCGCC and GGCGGG motifs in the *CitTPS16* promoter, indicating a crucial role for CitERF71 in the transcriptional regulation of *CitTP16* and, accordingly, in controlling the production of E-geraniol in citrus fruit (Li et al., 2017). Despite the several identified candidate terpene synthases and miRNAs involved in the biosynthesis of terpenes (Chen et al., 2018; Chen et al., 2020), little attention has been paid to those TFs involved in regulating the expression of monoterpene synthase in *BCC*.

In nature, plants often suffer from mechanical wounding (i.e., mechanical damage, hereon MD) and herbivory. To cope with these challenges, they have evolved advanced signaling systems and anti-herbivore defenses (Wu & Baldwin, 2010). One of the plant responses to wounding or herbivory are the mass release of volatile substances, which are important for enhancing resistance to insect enemies (Brilli et al., 2011; Portillo-Estrada & Niinemets, 2018; Rasulov, Talts & Niinemets, 2019). For example, when attacked by *Empoasca* (*Matsumurasca*) *onukii* Matsuda, oolong tea plants can release large amounts of linalool due to the up-regulation of linalool synthases (Mei et al., 2017). When *Gossypium hirsutum* was stimulated by mechanical wounding, production of terpenes and the expression of corresponding synthase genes were significantly induced (Yang et al., 2013). Concerning mechanical wounding, two monoterpene synthases from *Lilium* ‘Siberia’ showed considerable involvement in its floral defense, by inducing the emission of (Z)- *β*-ocimene and ( ±)-linalool, thereby increasing the accumulation of *LoTPS1* and *LoTPS3* transcripts (Abbas et al., 2019). Since MD can induce the release of volatile terpenes and upregulate the expression of corresponding terpene synthase enzymes, here we hypothesized that those TFs involved in regulating terpene biosynthesis would also be MD-inducible genes. Nevertheless, in *BCC*, we actually know rather little about how wounding might influence on the regulation of monoterpenes in its leaves.

To gain fresh insight into how wounds could influence *BCC* and to distinguish candidate genes and transcriptional factors involved in the monoterpene biosynthetic pathways, we performed for the first time a fully integrated analysis of its global transcriptome and metabolome profiles when challenged with MD. Hence, our results constitute a useful resource of potential genes and metabolites for investigating defense responses in *BCC* plants. Furthermore, our findings shed new light onto those genes involved in the biosynthesis and regulation of monoterpenes in *BCC* and provide a promising pool of candidate genes for analyzing the mechanism responsible for the accumulation of monoterpenes in *BCC*

## Material and methods

### Plant material, chemicals, and reagents

We purchased the borneol chemotype of *C. camphora* from the Ji’an Yu Feng Natural Species Co., Ltd (Ji’an, China), and these plants were grown in an artificial climate box (Shanghai Yiheng Instrument Co. Ltd, Shanghai, China), at 25 °C, with a 12-h-light/12-h-dark photoperiod. The D-borneol compound was bought from the Shanghai Yuanye Bio-Technology Co., Ltd (Shanghai, China).

### Mechanical damage (MD) treatment

Nine plants showing uniform growth and no disease symptoms were divided into three groups, each containing three biological samples. A stress treatment in the form of MD was applied to six of the nine *BCC* plants, by referring to two studies (Reymond et al., 2000; Ross, Kupper & Jacobs, 2006). Specifically, we used a medical hemostatic button to damage about 40% of each leaf’s area on a plant. These damaged leaves were then harvested at 2 h and 6 h (respectively, MD_2h and MD_6h) after wounding, leaving three undamaged BCC plants to serve as the control group (CK). We collected all their leaves and immediately froze them using liquid nitrogen and stored them at −80 °C until their use.

### Extraction of chemical contents

After imposing the MD treatment, the leaves collected at different times were quickly placed in liquid nitrogen and ground into a powder. From each sample, 0.15 g of powder was accurately weighed and put into an Eppendorf tube with 2.0 mL of petroleum ether. After 30 min of sonication (using an ultrasonic cleaner), the samples were centrifuged at 12,000 rpm for 10 min prior to their Gas Chromatography-Mass Spectrometer (GC/MS) analysis. Three technical replicates were performed for each of the three biological replicate samples.

### GC-MS analysis of chemical contents

To analyze the leaf extracts, we used an Agilent 7890B gas chromatograph equipped with a 5977A inert mass selective detector (Agilent, USA). Helium was the carrier gas, at a flow rate of one mL/min, while a Cyclosil-B GC column (30 m × 0.25 mm × 0.25 µm) at an initial temperature of 50 °C separated the analytes. The GC column’s temperature program consisted of an oven temperature rising from 50 °C to 180 °C at a rate of 2 °C/min, and then from 180 °C to 300 °C at 4 °C/min, then holding it at 300 °C for 4 min. For the injector, it was used in the split mode with a split ratio of 20:1, with an injection volume of 1.0 µL and an inlet temperature of 250 °C. To identify the ensuing components, their recorded mass spectra were compared to existing data stored in the NIST14/Wiley275 Mass Spectral Library. Further, we confirmed the presence of D-borneol by comparing its retention time to that of a known standard, this determined under the same GC/MS conditions above. The concentration of D-borneol per plant sample was calculated from its standard curve, while the contents of other components were quantified using D-borneol as an external standard. To determine the proportion of each component, its peak area/total peak area ratio was calculated.

### RNA extraction, cDNA library preparation, and sequencing

To extract the total RNA of the samples collected at each time point after the MD treatment, we used the EASYspin Plus Plant RNA kit (Aidlab, Beijing, China). To check the integrity and quantity of the RNA samples, 1% agarose gel electrophoresis and a NanoDrop 2000C Spectrophotometer (Thermo Scientific, USA) were respectively used. To qualify for the analyses, the RNA samples had to meet these criteria: an OD 260/280 = 1.8∼2.2, OD 260 / 230 ≥ 2.0, an RIN ≥ 6.5, and a 28S:18S ≥ 1.0; these were sent to Majorbio (Shanghai, China) to build the cDNA library after which transcriptome sequencing was carried out on an Illumina HiSeq 4000 platform (Illumina Inc., San Diego, CA, USA). This generated paired-end (PE) reads whose sequence quality was initially assessed using fastx_toolkit_0.0.14 software (http://hannonlab.cshl.edu/fastx_toolkit/). To clean the raw reads, we first removed any adaptor sequences, empty reads, and low quality sequences (i.e., those reads with unknown sequences ‘N’ or less than 25 bp in length), using SeqPrep (https://github.com/jstjohn/SeqPrep) and Sickle software (https://github.com/najoshi/sickle) set to their default parameters.

### *De novo* transcriptome assembly and annotation

Next, this high quality-sequenced clean data set underwent *de novo* assembly in TRINITY v2.5.0 software (https://github.com/trinityrnaseq/trinityrnaseq) set to default parameters, which integrates three independent software modules (Inchworm, Chrysalis and Butterfly) to process and splice large amounts of RNA-Seq data in turn. First, the Inchworm tool assembles the clean data into linear contigs; next, Chrysalis clusters the overlapping contigs into sets of connected components; finally, the Butterfly tool constructs the transcripts. The assembly results were then further filtered and optimized by TransRate (http://hibberdlab.com/transrate/), as well as clustered, to obtain the non-redundant unigenes via CD-HIT (http://weizhongli-lab.org/cd-hit/). For this assembly, we used BUSCO (Benchmarking Universal Single-Copy Orthologs, http://busco.ezlab.org) (Simao et al., 2015), set to its default parameters, to estimate the completeness of a conserved set of genes.

To annotate the transcripts, we used the BLAST program with an *E*-value cut-off of 1E^−5^, running against four public databases: NCBI non-redundant protein sequences (Nr, https://blast.ncbi.nlm.nih.gov/), Protein family (Pfam, http://pfam.sanger.ac.uk/), Swiss-Prot (http://web.expasy.org/docs/swiss-protguideline.html), and Clusters of Orthologous Groups of proteins (COG, http://www.ncbi.nlm.nih.gov/COG/). Functional annotation according to the Gene Ontology (GO) terms was implemented in Blast2GO v2.5.0 (http://www.geneontology.org). We also performed a Kyoto Encyclopedia of Genes and Genomes (KEGG) pathways’ analysis, in KOBAS v2.1.1 (http://www.genome.jp/kegg/), to systematically explore the functions of the genes. Their transcripts were also blasted with the PlantTFDB (http://planttfdb.cbi.pku.edu.cn/), using the HMMER method, to obtain the homologous TFs and their family information.

### Differential gene expression analysis

To estimate the relative expression levels, the FPKM (fragments per kilo bases per million reads) of transcripts were determined in RSEM v1.2.15 software (http://deweylab.github.io/RSEM/) set to its default parameters. The raw counts were statistically analyzed directly, in DESeq2 v1.10.1 software, using a negative binomial distribution. Those transcripts having a significantly different expression between pairs of treatment groups were obtained by using threshold parameters of a P-adjusted <0.05 and —log2FC— ≥ 1. Next, these designated differentially expressed genes (DEGs) underwent a KEGG enrichment analysis in KOBAS v2.1.1 (http://www.genome.jp/kegg/).

### Screening of candidate TPS genes and TFs related to monoterpene biosynthesis

To screen out candidate TPS genes, we first selected those unigenes directly annotated as TPSs by KEGG. Then, a local BLASTP analysis for candidate TPSs sequences was carried out against the unigenes, using previously reported related sequences from other plants. The selected unigenes had an identity >40%, bit score >100, and a sequence length >800 bp. Furthermore, to locally search for unigenes in the Pfam-A database, we used hmmsearch in HMMER 3.0 for the two Pfam domains (PF01397 and PF03936), with an e-value cut-off of 1 × 10^−5^. To not overlook any TPS candidates, the TERZYME program was relied upon to identify and classify TPSs (Priya et al., 2018). Finally, we integrated results of the KEGG annotation, BLASTP, HMM and TERZYME searches, and all candidate TPS genes were further examined to confirm the presence of both PF01397 and PF03936 domains using the Pfam database and the NCBI’s conserved domains database (CDD). We subjected the candidate CcTPSs to a phylogenetic analysis—this done in MEGA6.0, using the maximum likelihood (ML) method with *n* = 1000 bootstrap replicates and best models of JTT+G. The resulting tree’s presentation was augmented in EvolView v3 (Subramanian et al., 2019). The conserved motifs of CcTPSs were discovered using MEME (http://meme-suite.org/tools/meme). Further, to obtain the Spearman correlation coefficients between terpene synthases and volatile terpenoids, we used IBM SPSS 19.0 (Armonk, NY, United States). Finally, using Cytoscape (Shannon et al., 2003), a gene–metabolite network map was constructed according to those Spearman results (using only correlation coefficients >0.6, *P*-values <0.05).

To uncover those TFs associated with monoterpnene biosynthesis, we tested the differentially expressed AP2/ERF-, WRKY-, bZIP-, BHLH-, MYB- family genes for their correlation with the candidate monoterpene synthase. This Spearman correlation analysis was done with expression data for TF genes and candidate monoterpene synthase (also in IBM SPSS 19.0). For those Spearman correlation coefficients >0.8 with a *P*-value <0.05, a graphic of the putative TPSs–TFs networks were constructed in Cytoscape. The sequences of those TFs positively correlated with monoterpene synthases were submitted online to the website ORF finder (https://www.ncbi.nlm.nih.gov/orffinder/), to determine the presence of full-length sequences. Then, the deduced amino sequences were submitted to the online website of PfamScan (https://www.ebi.ac.uk/Tools/pfa/pfamscan/), to determine the presence of conserved domains.

### Gene expression analysis by quantitative real-time PCR (qRT-PCR)

To verify the reliability of the obtained RNA sequencing data, we relied on qRT-PCR, as previously described (Yang et al., 2020). The expression levels of 23 DEGs—9 related to the biosynthesis of monoterpenes, 6 TFs, and 8 related to the jasmonic acid (JA) signaling pathway—were evaluated using the 2^−△△Ct^ method, with CcActin serving as the reference gene. The measurements from three biological replicates were performed in triplicates. All primers of the 23 unigenes, designed with Primer Premier 5.0 software, can be found in Table S1.

**Figure 1:**
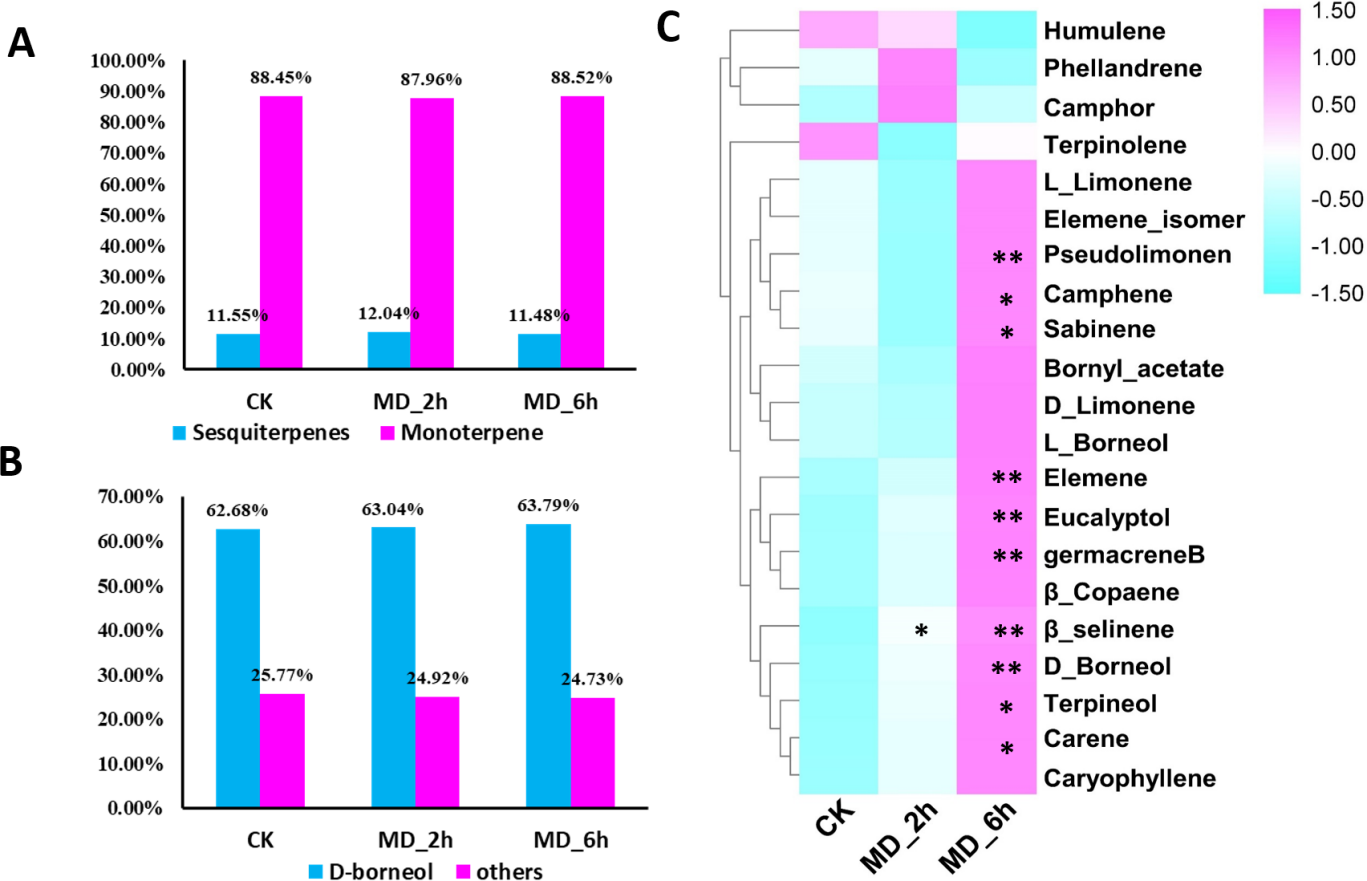
Volatile terpene analysis of *BCC* in response to mechanical damage. (A) The ratios of monoterpene to sesquiterpene in CK, MD_2 h and MD_6 h group of *BCC*; (B) the ratios of D-boreneol in each group; D Comparisons of the relative abundance of the identified volatile terpene among different treatment groups, an asterisk (*) indicated *p* < 0.05 when compared with the CK group while two asterisks (**) indicated that *P* < 0.01 when compared with the CK group.

## Results

### Mechanical damage (MD) increased the production of terpenes

Higher contents of terpenes in MD-treated plants relative to the CK group were evidenced except for one monoterpene and one sesquiterpene. Specifically, from *BCC* leaves we detected a total of 21 terpenes induced by MD treatment, including 14 monoterpenes (accounting for 88%, of which 63% was D-borneol) and 7 sesquiterpenes (accounting for 11%), using GC/MS (Fig. 1A, 1B, Table S2). Seventeen terpenes (11 monoterpenes, 6 sesquiterpenes) had the highest content 6 h after the MD treatment, and for 2 monoterpenes their content peaked after 2 h and then declined by 6 h post-MD. Furthermore, the carene, pseudolimonene, camphene, sabinene, eucalyptol, D-borneol, terpineol, elemene, and germacrene B contents were all prominently enhanced in the MD_6h samples compared with the CK group, while their levels in the MD_2h samples also tended to surpass those of CK, albeit not significantly (Fig. 1C).

### Transcriptome sequencing and annotation, expression analysis of unigenes

**Figure 2:**
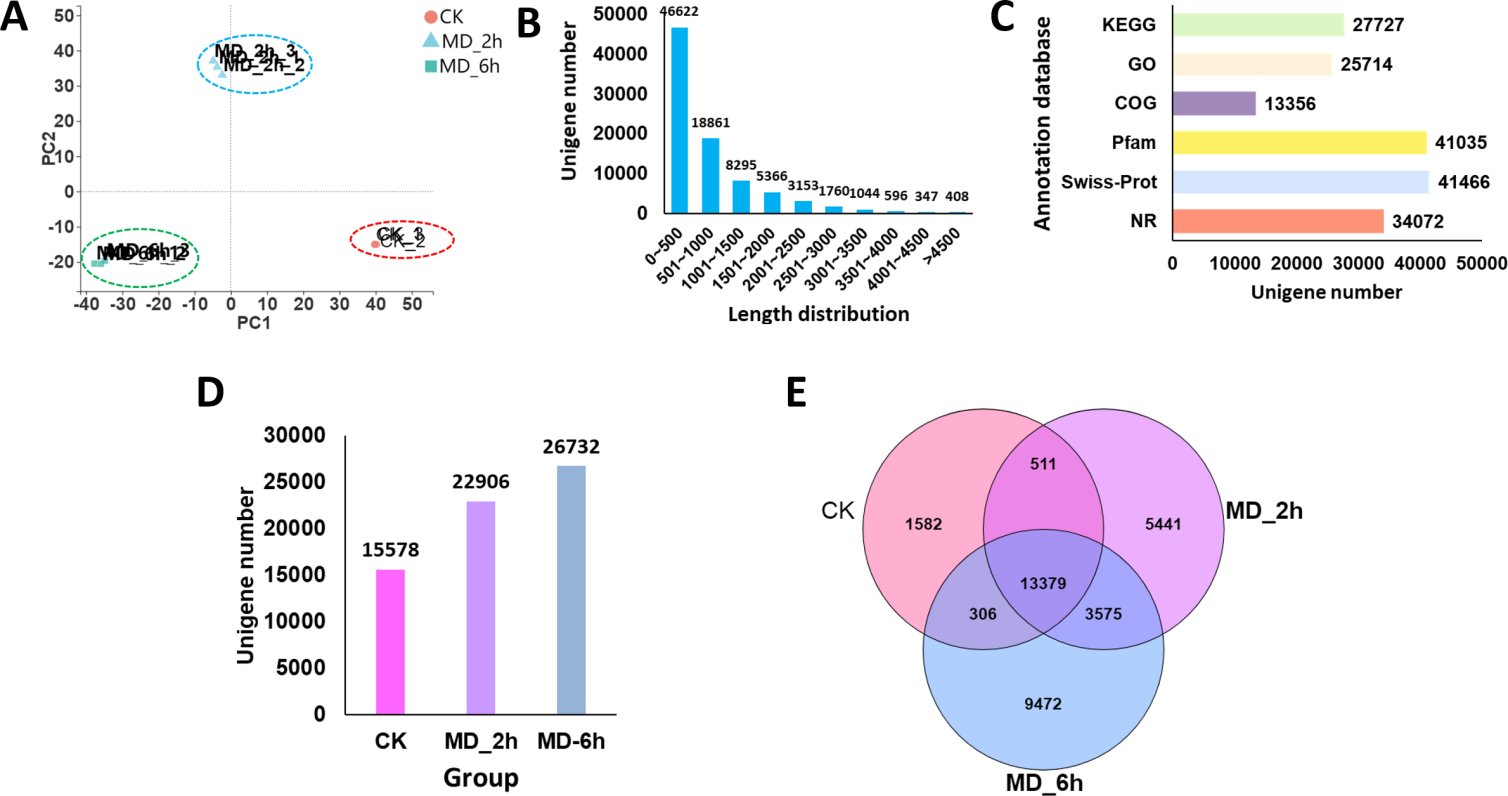
Overview of *BCC* leave transcriptomes exposed to mechanical damage. (A) PCA analysis of the sequencing data from the three replicates ×three different treatment samples; (B) length distribution of the assembled unigenes; (C) functional annotation of the unigenes against six database; (D) Unigenes expressed in different groups; (E) Venn diagram showing the numbers of common and specific expressed unigenes among different groups.

Based on the ability of MD to increase D-borneol and other monoterpenes’ production, deep transcriptome sequencing of the induced *BCC* collected at different hours was performed, as well as the CK group. In total, 67.66 Gb of high-quality base pair data (at least 7.51G per sample and 22.55 G for each group), with a GC content of 48.62%, were obtained (Table 1). Our PCA analysis of the resulting transcription data revealed a clear separation of the MD_6 h group from both MD_2 h and CK groups, suggesting a high repeatability of the sequencing data (Fig. 2A).

**Table 1:** Summary transcritome sequencing statistics of the MD induced BCC.

Sample	Raw reads	Clean reads	Clean bases	Q20 (%)^a^	Q30 (%)^b^	GC content (%)	Mapped ratio
CK_1	49,034,254	48,633,398	7.25G	98.3	94.59	48.97	81.63%
CK_2	50,352,456	49,936,518	7.46G	98.18	94.3	48.78	80.93%
CK_3	46,381,336	45,903,254	6.83G	97.93	93.68	48.57	82.81%
MD_2 h_1	50,057,520	49,718,252	7.41G	98.22	94.37	48.37	81.27%
MD_2 h_2	52,363,340	51,989,792	7.76G	98.2	94.34	48.61	80.91%
MD_2 h_3	61,241,348	60,842,136	9.07G	98.31	94.59	48.28	81.66%
MD_6 h_1	52,773,758	52,351,000	7.79G	98.2	94.34	48.14	81.39%
MD_6 h_2	48,306,288	47,903,474	7.14G	98.16	94.23	48.65	80.03%
MD_6 h_3	47,063,856	46,638,554	6.95G	98.12	94.16	49.17	78.84%
Summary	457,574,156	453,916,378	67.66G			48.62	81.05%

**Notes.**

a Q20 indcates the percentage of bases with a Phred value >20 while.

b Q30 indicates the percentage of bases with a Phred value >30.

A total of 86,452 unigenes were assembled *de novo,* these having a mean length of 837 bp and an N_50_ of 1,300 bp (Table 2). Most of the unigenes (65,483; 75.74%) were shorter than 1,000 bp, while only a few genes (7,308; 8.4%) were longer than 2,000 bp (Fig. 2B). We then evaluated the quality of the assembled sequences by examining the mapped ratio of the clean data to the assembled unigenes of each sample using RSEM. The result showed that 81.05% of the clean reads could be perfectly mapped to the reference unigenes, indicating the throughputs and sequencing quality were sufficient to ensure the reliability of further analyses (Table 1). Moreover, the ‘TransRate’ assembly score, which aims to assess the accuracy and completeness of transcriptome assembly, reached 31.45% for *C. campora*, a value higher than that of many of other assemblies (Smith-Unna et al., 2016). We also assessed the assembly completeness using BUSCO, this lent further support to the high quality of the assemblies, in which 80.70% of single-copy orthologs were successfully captured (Table 2). All RNA-Seq raw data was submitted to CNGBdb (accession number CNP0000956) and NCBI (accession number PRJNA679978). And the the de novo transcript assemblies was submitted to CNGBdb (accession number CNA0020891, https://db.cngb.org/search/project/CNP0000956/).

**Table 2:** Evaluation of assembly results.

Type	Resource
Total unigenes number	86,452
Total sequence base	101,714,598 bp
Largest	15,727 bp
Smallest	201 bp
Average length	837.56 bp
N50	1,300 bp
E90N50	1,987 bp
GC percent	45.70%
TransRate score	31.45%
Complete BUSCO socres	84.20%
Complete—single-copy BUSCO socres	80.70%
Complete—duplicated BUSCO socres	3.50%

Using the BLAST algorithm, sequence annotations were performed against the NR, Swiss-Prot, Pfam, COG, GO, and KEGG databases. From this, 27,727 unigenes (32.07%) were annotated by the KEGG database, 25,714 unigenes (29.74%) were identified in the GO database, 13,356 unigenes (15.45%) were annotated against the COG database, and 34,072 unigenes (39.41%) had significant matches to the NR database. In addition, 41,035 (47.47%) and 41,466 (47.96%) of the unigenes displayed significant similarities to known proteins in the Pfam and Swiss-Prot database, respectively (Fig. 2C). When summed, we found 15,578, 22,906, and 26,732 of the unigenes expressed in the CK, MD_2h, and MD_6h groups, respectively (Fig. 2D). Among those, 1,582 unigenes were expressed only in the CK group, 5,441 specifically in the MD_2h group, and 9,472 unigenes uniquely so in the MD_6h group (Fig. 2E), indicating that the MD treatment was able to significantly induce the expression of genes in *BCC*. These results provided a general overview of the assembled transcriptome that we then used in our further analysis of DEGs.

### Comparing the DEGs induced by MD treatment to the enrichment analysis

To fully explore potential DEGs induced by the MD treatment, we compared three groups of *C. camphora* plants. The DEGs were designated according to an expression level of —log 2 (fold-change) —>1 and FDR <0.05 in each pairwise comparison. This revealed 12,180 (9,643 upregulated, 2,537 downregulated) and 13,466 (9,222 upregulated, 4,244 downregulated) unigenes that were significantly differentially expressed relative to the control samples in the MD-treated samples at 2 h and 6 h post-treatment, respectively. Between MD_2h and MD_6h, we identified 10,477 unigenes significantly expressed (4,570 upregulated, 5,907 downregulated) (Figs. S1A–S1C). Among the three pairwise comparisons, 2,539 genes were commonly regulated. Finally, a total of 19,149 DEGs were screened among the three comparison groups (Fig. S1D).

To exhaustively explore the presumed functions of the above DEGs, we then performed KEGG and GO enrichment analyses to uncover overrepresented pathways and biological functions in the BCC leaves as induced by their mechanical damaging. Based on the GO analysis, within the main molecular function (MF), cellular component (CC), and biological process (BP) categories the most enriched GO terms of the upregulated DEGs in the CK vs. MD_2 h comparison were protein serine/threonine phosphatase activity, recognition of pollen, and intrinsic component of membrane (Table S3); in the CK vs. MD_6 h comparison, the over-presented GO terms contained genes were related to transcription, the DNA-templated extracellular region, and heme binding activity (Table S4). For the MD_2 h vs. MD_6 h comparison, the largest percentages of unigenes identified within the CC, BP, and MF categories were extracellular region, reactive oxygen species metabolic process, and monooxygenase activity (Table S5).

**Figure 3:**
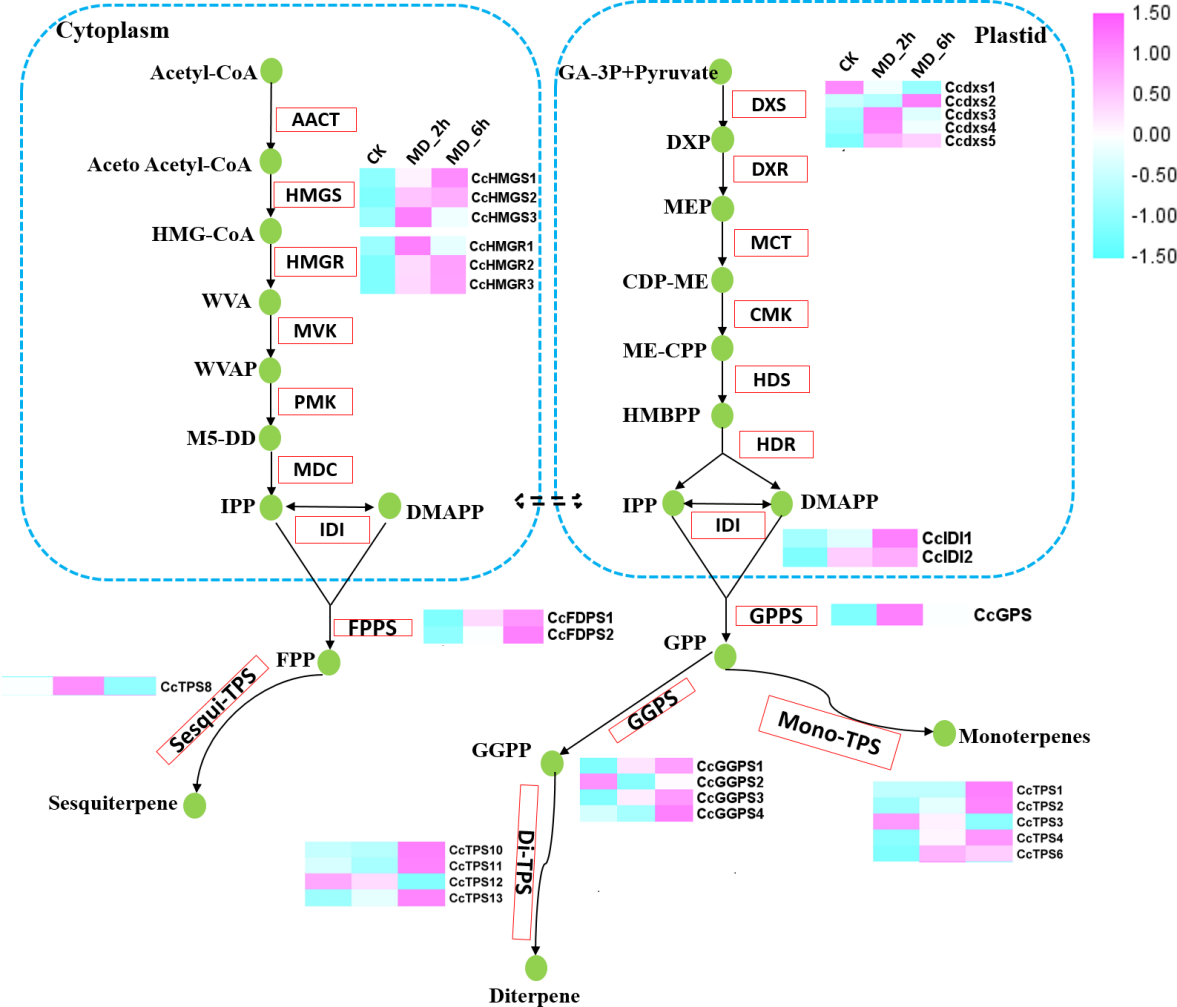
Differential expression of the unigenes related to the terpenoid biosynthesis. Enzymes related to the terpenoid biosynthesis were shown in red frames, and the compounds were shown in green circle. Enzymes with similar functions are listed in brackets. The expression pattern was shown within three columns, with the left column representing the CK group, the middle column representing the MD_2h group and the right representing the MD_6h group. Acetyl-CoA C-acetyltransferase (CCAT); HMGS, Hydroxymethylglutaryl-CoA synthase; HMGR, Hydroxymeth-ylglutaryl-CoA reductase; MVK, Mevalonate kinase; PMK, Phosphomevalonate kinase; MVD, Diphosphomevalonate decarboxylase; DXS,1-deoxy-D-xylulose-5-phosphate synthase; DXR, 1-deoxy-Dxylulose-5-phosphate reductoisomerase; MCT, 2-C-methyl-D-erythritol 4-phosphate cytidylyltransferase; CMK, 4-diphosphocytidyl-2-C-methyl-D-erythritol kinase; MDS, 2-C-methyl-D-erythritol 2,4-cyclodiphosphate synthase; HDS, (E) -4-hydroxy-3-methylbut-2-enyl-diphosphate synthase; HDR, 4-hydroxy-3-methylbut-2-en-1-yl diphosphate reductase; IDI, isopentenyl-diphosphate Delta-isomerase.

Additionally, KEGG pathway enrichment analyses showed that the upregulated DEGs from the CK vs. MD_2h comparison could be assigned to 128 pathways, while the top 3 enriched pathways were “plant-pathogen interaction”, “plant hormone signal transduction”, and “phenylpropanoid biosynthesis” (Fig. S1E, Table S6). In comparing the CK with the MD_6 h group, the most overrepresented pathways for the upregulated DEGs were “phenylpropanoid biosynthesis”, “alpha-linolenic acid metabolism”, “plant-pathogen interaction”, “phenylalanine, tyrosine, and tryptophan biosynthesis”, and “selenocompound metabolism” (Fig. S1F, Table S7). Regarding the MD_2h vs. MD_6h comparison, the KEGG pathways of “ribosome”, “phenylpropanoid biosynthesis”, “plant-pathogen interaction”, “MAPK signaling pathway-plant”, and “flavonoid biosynthesis” were the five most enriched (Fig. 3S1G, Table S8).

### Identifying the DEGs related to terpene biosynthesis induced by MD

To further assess the effect of MD induction on volatile terpene biosynthesis, we next focused on the expression pattern of genes involved in the biosynthesis of terpenes. A total of 20 genes participating in the terpene backbone synthesis pathway had their activities significantly altered by MD. These 20 DEGs consisted of 3 homologues of HMGR, 3 homologues of HMGS, 5 homologues of DXS, 2 homologues of IDI, 1 homologues of GPPS, 2 homologues of FPPSs, and 4 homologues of GGPPSs. The expression of each DEGs was substantially increased in MD_2h or MD_6h when compared with the CK group, except for one putative DXS (CcDXS1) and one GGPPS (CcGGPPS2) (Fig. 3, Table S9).

**Figure 4:**
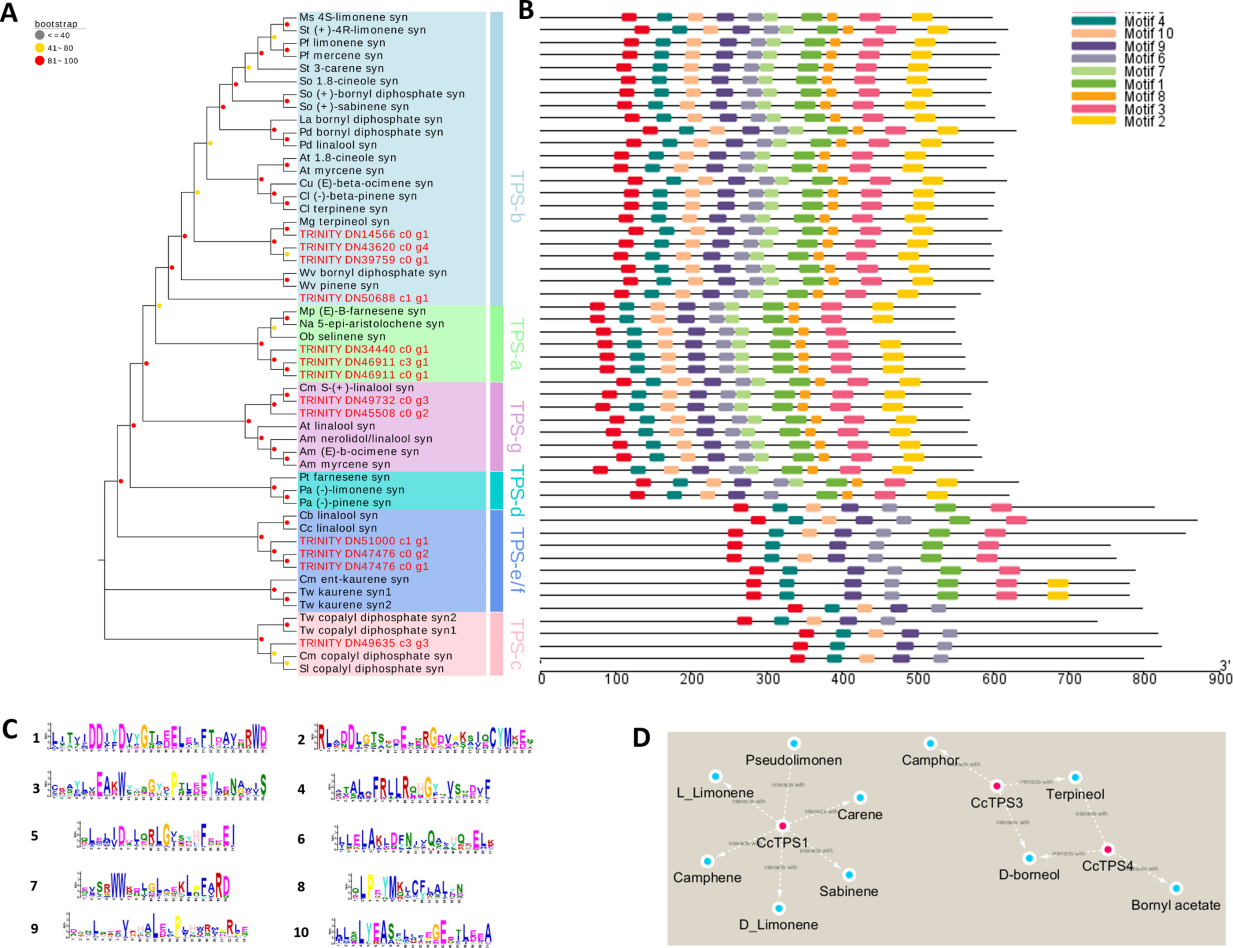
Phylogenetic analysis and correlation analysis of the CcTPS. (A) Phylogenetic analysis of amino acid sequences of CcTPS along with other characterized plant TPS. TPS identified in this study were Highlighted with red dots; (B) comparison of CcTPS motif; (C) the amino acid composition corresponding to the 10 motifs in Figure B; (D) interaction networks between the ten CcTPS and 21 volatile terpenes.

We spotted 13 TPS-encoding unigenes (Table 3). According to our phylogenetic analysis based on the deduced amino acid sequences in combination with well-characterized terpene synthase genes, the 13 *BCC* TPSs could be divided into five subfamilies (Fig. 4A). The *CcTPS1* (TRINITY_DN14566_c0_g1), *CcTPS2* (TRINITY_DN50688_c1_g1), *CcTPS3* (TRINITY_DN43620_c0_g4), and *CcTPS5* (TRINITY_DN39759_c0_g1) all belong to the TPS-b subfamily, which are responsible for the synthesis of cyclic monoterpenes and contain the RR (X) 8W motif in their N-terminal region. Both *CcTPS4* (TRINITY_DN45508_c0_g2) and *CcTPS6* (TRINITY_DN49732_c0_g3) could be assigned to the TPS-g subfamily, which produces acyclic monterpenes and lacks the RRX8W motif in their N-terminal region. Another three, *CcTPS7* (TRINITY_DN34440_c0_g1), *CcTPS8* (TRINITY_DN40799_c0_g1), and *CcTPS9* (TRINITY_DN46911_c0_g1), are members of the TPS-a subfamily clade, which may encode a sesquiterpene synthase. Next, *CcTPS10* (TRINITY_DN47476_c0_g1), *CcTPS11* (TRINITY_DN47476_c0_g2), and *CcTPS13* (TRINITY_DN51000_c1_g1) belong to the TPS-e/f subfamily, while only *CcTPS12* (TRINITY_DN49635_c3_g3) belongs to the TPS-c subfamily that is responsible for the synthesis of diterpenes. Using the MEME program, the conserved motif analysis revealed that those TPS members clustering in the same subfamily showed similar motif characteristics (Figs. 4B–4C). Of these 14 TPSs, 10 were defined as differentially expressed TPS genes, whose expression pattern were shown Fig. 3. We used these 10 putative TPSs, comprising 5 putative monoterpenoid synthase, 1 sesquiterpenoid synthase, and 4 diterpenoid synthase genes, in the further analyses.

**Table 3:** Table of candidate TPS genes of BCC.

Gene ID	Rename	Length/ aa	Pfam ID	Domain	Putative function	BLASTP	*E* value	Identity	Accession number
TRINITY_DN14566_c0_g1	CcTPS1	612	PF01397.21, PF03936.16	Terpene_synth, Terpene_synth_C	Monoterpene synthase	Alpha-terpineol synthase, chloroplastic	0	0.9624	RWR83481.1
TRINITY_DN50688_c1_g1	CcTPS2	583	PF01397.21, PF03936.16	Terpene_synth, Terpene_synth_C	Monoterpene synthase	(-)-alpha-terpineol synthase	0	0.5111	Q6PWU2.1
TRINITY_DN43620_c0_g4	CcTPS3	597	PF01397.21, PF03936.16	Terpene_synth, Terpene_synth_C	Monoterpene synthase	alpha-terpineol synthase	0	0.8342	RWR97839.1
TRINITY_DN45508_c0_g2	CcTPS4	559	PF01397.21, PF03936.16	Terpene_synth, Terpene_synth_C	Monoterpene synthase	S-+-linalool synthase	0	0.9749	RWR97874.1
TRINITY_DN39759_c0_g1	CcTPS5	600	PF01397.21, PF03936.16	Terpene_synth, Terpene_synth_C	Monoterpene synthase	Geraniol synthase	0	0.9349	Q8GUE4.2
TRINITY_DN49732_c0_g3	CcTPS6	571	PF01397.21, PF03936.16	Terpene_synth, Terpene_synth_C	Monoterpene synthase	S-+-linalool synthase	0	0.9439	RWR93550.1
TRINITY_DN34440_c0_g1	CcTPS7	558	PF01397.21, PF03936.16	Terpene_synth, Terpene_synth_C	Sesquiterpene synthase	Beta-cubebene synthase	2E-123	0.57	B3TPQ6.1
TRINITY_DN40799_c0_g1	CcTPS8	568	PF01397.21, PF03936.16	Terpene_synth, Terpene_synth_C	Sesquiterpene synthase	(-)-germacrene D synthase	0	0.4954	Q6Q3H3.1
TRINITY_DN46911_c0_g1	CcTPS9	562	PF01397.21, PF03936.16	Terpene_synth, Terpene_synth_C	Sesquiterpene synthase	terpene synthase 1	0	0.8627	RWR93512.1
TRINITY_DN47476_c0_g1	CcTPS10	755	PF01397.21, PF03936.16	Terpene_synth, Terpene_synth_C	Diterpene synthase	diterpene geranyllinalool synthase	0	0.9734	RWR96469.1
TRINITY_DN47476_c0_g2	CcTPS11	763	PF01397.21, PF03936.16	Terpene_synth, Terpene_synth_C	Diterpene synthase	diterpene geranyllinalool synthase	0	0.9788	RWR96468.1
TRINITY_DN49635_c3_g3	CcTPS12	819	PF01397.21, PF03936.16	Terpene_synth, Terpene_synth_C	Diterpene synthase	ent-copalyl diphosphate synthase, chloroplastic-like protein	0	0.9815	RWR73750.1
TRINITY_DN51000_c1_g1	CcTPS13	855	PF01397.21, PF03936.16	Terpene_synth, Terpene_synth_C	Diterpene synthase	diterpene geranyllinalool synthase	0	0.8224	RWR88024.1

The FPKM values of the 10 candidate TPS genes and the content of terpenes were integrated and a correlation analysis was performed to clarify the relationship between them. According to the ensuing correlation networks (Fig. 4D, Table S10), *CcTPS1* was positively associated with six monoterpenes (carene, pseudolimonene, camphene, sabinene, L-limonene, D-limonene) as was *CcTPS3* and *CcTPS4* with four monoterpenes (borneol, bornyl acetate, camphor, terpineol). Presumably, CcTPS1 might directly influence the biosynthesis of carene, pseudolimonene, camphene, sabinene, L-limonene, and D-limonene in *BCC* leaf tissue, while CcTPS3 may partake in the biosynthesis of borneol, camphor, and terpineol, with CcTPS4 perhaps participating in the biosynthesis of borneol, terpineol, and bornyl acetate.

### Identifying candidate transcription factor in response to MD

**Figure 5:**
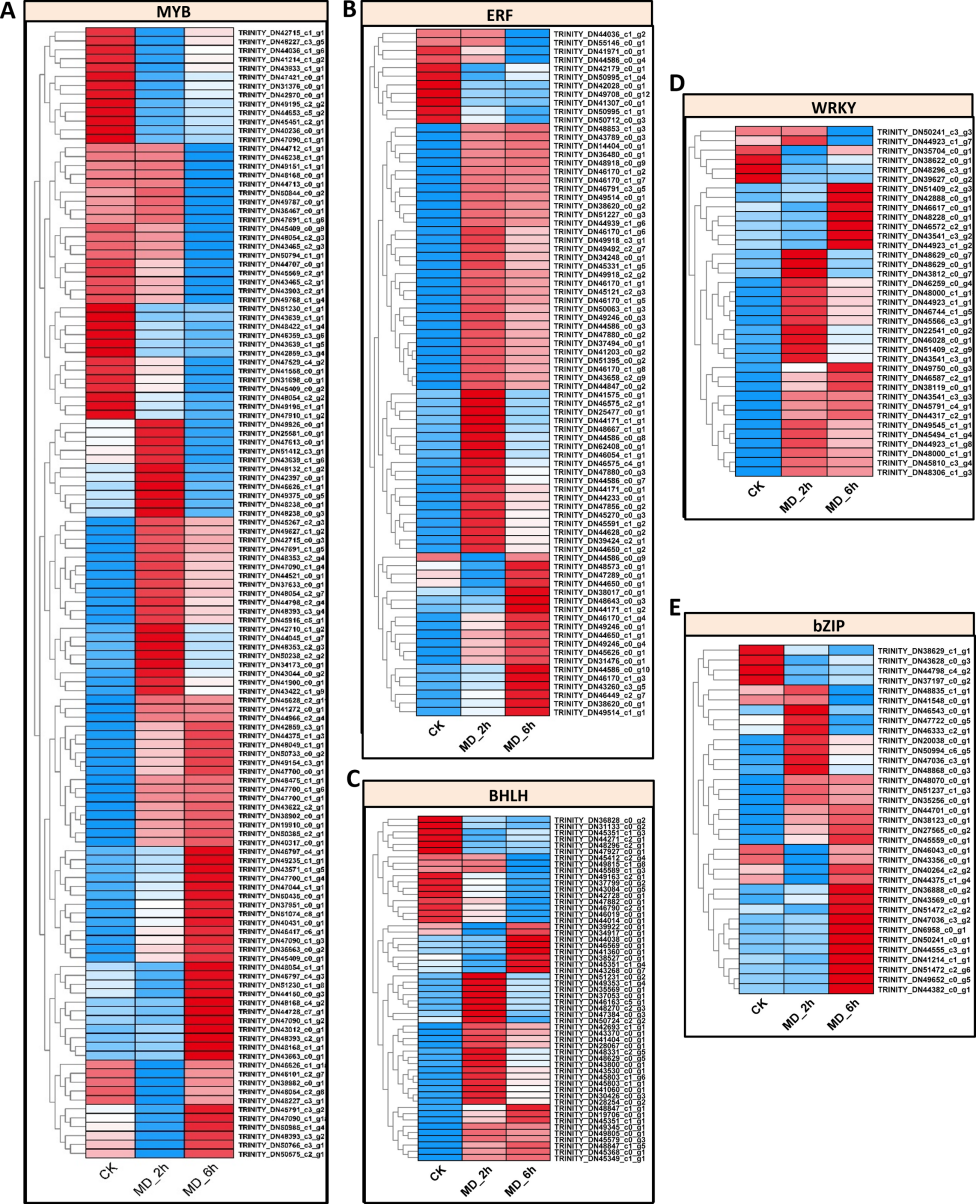
Identification of TFs which were response to MD. (A–E) Expression pattern of the BHLH-, bZIP-, AP2/ERE-, MYB-, WRKY- genes, respectively.

Since transcriptional reprogramming is primarily controlled by TFs, we further identified the differentially expressed TFs among the three treatment comparison groups. In this study, 673 differential expressed putative TFs belonging to 30 families were identified in *BCC* among the three group comparisons. There are about 58 TF families in higher plants, for which the major TF families, such as AP2/ERF, bHLH, MYB, WRKY, and bZIP, are linked with responses to biotic and abiotic stresses (Karanja et al., 2019; Lin et al., 2013; Liu & Liu, 2020; Pandey et al., 2017; Zhao et al., 2011). In line with other published findings, we found many TFs of the MYB family (127 genes), along with those of the AP2/ERF family (81 genes), BHLH family (55 genes), WRKY family (37 genes), and bZIP family (35 genes) (Table S13). The expression patterns of these five TF families were depicted in Figs. 5A–5E and conveyed in Table S14, which indicated that most of the unigenes in these five TF families (82/127 for MYB, 70/81 for AP2/ERF, 38/55 for BHLH, 31/37 for WRKY, and 29/37 for bZIP) were upregulated in the MD-treated samples at either 2 h or 6 h vis-à-vis the control samples. These screened TFs offered a pool of candidates that can be harnessed to potentially enhance plant resistance to MD.

### Mining of candidate transcription factors related to monoterpenoid biosynthesis

**Figure 6:**
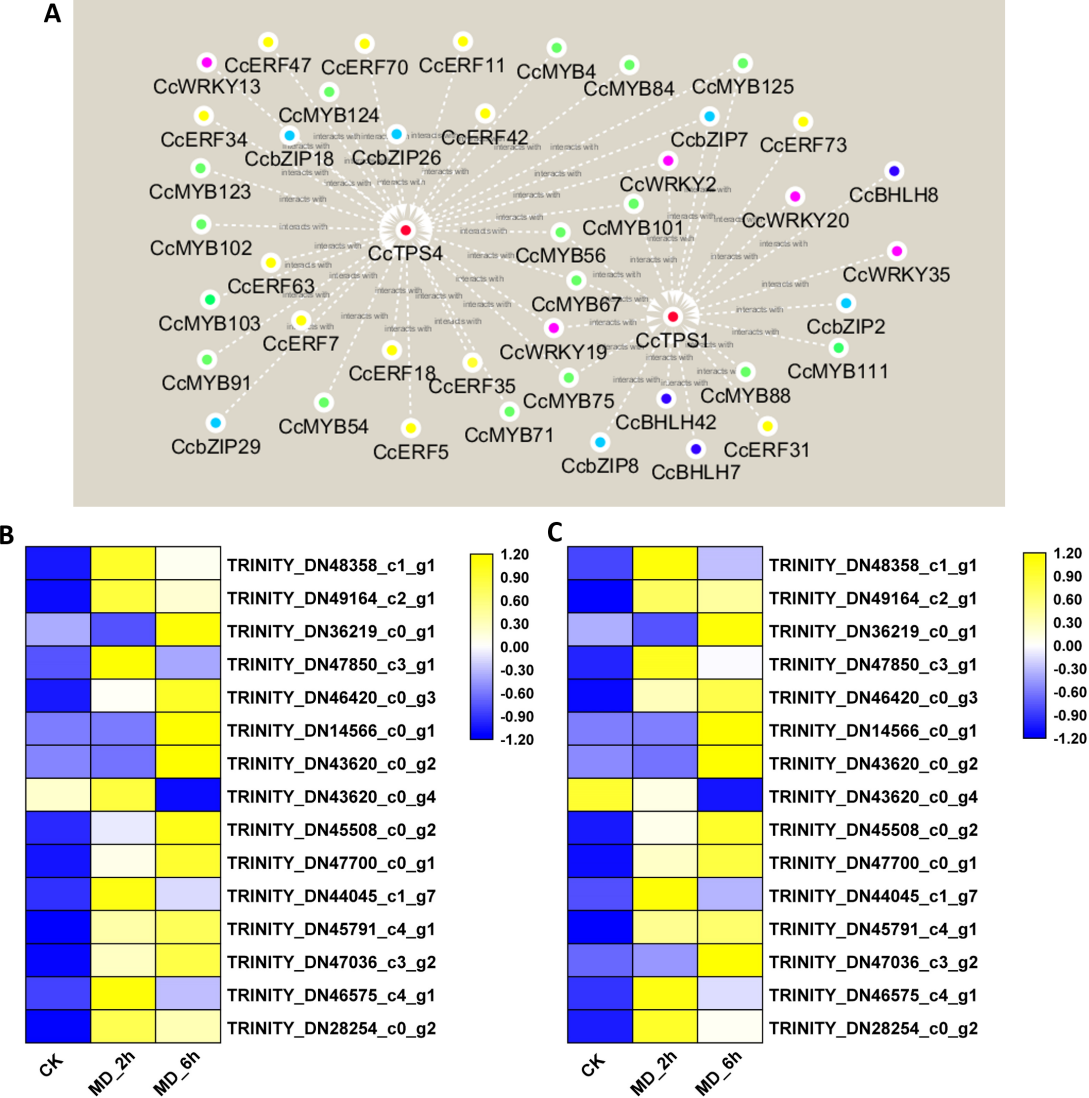
Interaction networks between CcTPS1, CcTPS2 and 42 putative TF unigenes and qRT-PCR veridation. (A) Interaction networks between CcTPS1, CcTPS2 and 42 putative TF unigenes , The red, green, yellow, purple, sky blue and blue color indicated the CcTPS, CcMYB, CcERF, CcWRKY, CcbZIP, CcBHLH genes, respectively. (B) qRT-PCR results of the 15 selected genes; (C) FPKM results of the 15 selected genes.

Recently, the MYB-, bHLH-, ERF-, WRKY-, and bZIP-type TFs were shown capable of regulating the expression of plant monoterpene synthase genes (Sarker, Adal & Mahmoud, 2019). To select candidate TFs able to regulate the expression of monoterpene synthase in *BCC*, the abovementioned 335 DEGs belonging to these five TF families were submitted to a gene co-expression network analysis with the three selected candidate monoterpene synthase genes (*CcTPS1, CcTPS3, CcTPS4*). The results showed that three homologues of the BHLH family, six homologues of the bZIP family, five homologues of the WRKY family, 12 homologues of the ERF family, and 16 homologues of the MYB family all had strong, positive correlation coefficient values (R^2^-values > 0.8) with *CcTPS1* and *CcTPS4*. In stark contrast, no TFs were found positively related to *CcPTS3*. Based on these results, interaction networks between the two monoterpene synthase and 42 transcription factors were organized (Fig. 6A, Table S15). Building on this, we carried out a more in-depth analysis of the sequences of these candidate TFs. First, by using the ORF finder, 37 of the TFs corresponding to 5 WRKY genes, 15 MYB genes, 10 ERF/AP2 genes, 5 bZIP genes, and 2 BHLH genes were discovered to contain a complete ORF. Next, the deduced amino acid sequences of each of these 37 TFs were submitted to the PfamScan, to determine whether a given sequence contained the conserved domains of the corresponding gene family. As a result, the 5 WRKY all contained complete WRKY domains (PF03106.15) and likewise, the 15 MYB genes all harbored a MYB DNA-binding domain with a Pfam ID of PF00249.31. The 10 ERF/AP2 genes, five bZIP genes, and two BHLH genes were confirmed to belong to their corresponding gene families since their respective AP2 (PF00847.20), bZIP_1 (PF00170.21), and HLH (PF00010.26) domains were identified. Taken together, these 37 TFs may play important roles in regulating monoterpene synthase expression to affect monoterpene biosynthesis in *BCC*. Therefore, this promising pool of candidate TFs is the focus of our future research.

### qRT-PCR validation of DEGs from the RNA-Seq analysis

To validate the reliability of the RNA-Seq data, we chose 15 genes (nine monoterpene biosynthesis related genes, six transcription factors) to conduct the qRT-PCR analysis. These results basically agreed with the above transcriptome results; hence, our original analysis of the *C. camphora* transcriptome is robust (Fig. 6B).

## Discussion

In recent years, there has been an enormous number studies studying genes involved in terpene biosynthesis in different plant species. Being a genus rich in terpenoids, the terpenoid biosynthetic pathways in the *Cinnamomum* genus have drawn the attention of many researchers. For example, a geraniol synthase, CtGERS, was cloned from *C. tenuipilum* by using a homology-based cloning strategy and its function verified by *in vitro* enzyme activity (Yang et al., 2005). Later, the linalool synthase (LIS) genes were isolated from different provenances of *C. osmophloeum*: the recombinant proteins of LIS are capable of generating the S-(+)-linalool from GPP and (E)-nerolidol from FPP (Lin et al., 2014). According to a transcriptome analysis on their leaves, the expression levels of three TPS14-like (TPS14-like1-3) genes in borneol chemotypes of *C. camphora* significantly exceed those of other conspecific chemical types (Chen et al., 2018). In an analysis of the full-length transcriptome of the three different chemical types (camphor, linalool, eucalyptol) of *C. porrectum*, five genes (*CpTPS1, CpTPS3, CpTPS4, CpTPS5*, and *CpTPS9*) had specific expression or higher expression in one of the chemotypes and may be involved in monoterpene biosynthesis (Qiu et al., 2019). Our previous transcriptomic study of three different chemotypes of *C. burmannii* indicated that two monoterpene synthase unigenes, designated *CbTPS2* and *CbTPS3*, were upregulated in the high-borneol group when compared with the low-borneol and borneol-free groups, suggesting they might be vital to the biosynthesis of D-borneol in that species (Yang et al., 2020). The above findings contributed to a better understanding of the mechanism of differential accumulation of specific terpenoids in different chemotypes of *Cinnamomum* plants. In this study, a bioinformatics analysis of RNA sequences explored the potential gene-TFs regulatory network in leaves of *C. camphora*.

It is known that genes related to the biosynthetic pathway of secondary metabolites in plants are usually coordinated and regulated, a phenomenon readily inferred and recognized via the joint analysis of RNA-Seq and metabolome data (Nett, Lau & Sattely, 2020). In the context of co-expression analysis, here we used mechanical damage as an induction method to observe the changed content of volatile metabolites and the expression of related genes and TFs, from which candidate genes could be distinguished through statistically significant correlations. To be sure, many transcriptome analyses in response to mechanical damage and herbivory have been reported on many plants, such as *Arabidopsis thaliana* (Cheong et al., 2002; Reymond et al., 2000), *Nicotiana attenuate* (Hui et al., 2003), cotton (Huang et al., 2015), and cucumber (Mercke et al., 2004). These studies showed that plants produce a stress response within minutes and hours after being mechanically damaged (Pandey et al., 2017). According to the literature, the jasmonic acid (JA), which may induce the biosynthesis of volatile terpenes, usually reaches peak values within 1 to 2 h after the wounding of plants (Zhang et al., 2020). Likewise, 2 h after the wounding, green leaf volatiles may be released in large amounts (Ping et al., 2001). Further, some terpene synthases and volatile terpenes quickly respond to wounding injury, within 6 to 8 h (Abbas et al., 2019). Similarly, in our experiment, we did find that the volatile terpenoids had changed 2 to 6 h since imposing the MD treatment to *BCC*. Because these changes arise from differing transcription levels, we selected both times (MD_2h, MD_6h) for conducting the transcriptome analysis.

From our results, we identified three TPS genes (*CcTPS1, CcTPS3, CcTPS4*) and 37 transcription factors (5 WRKY, 15 MYB, 10 ERF/AP2, 5 bZIP, 2 BHLH) genes that might be critically involved in the biosynthesis and regulation of monoterpenes in *BCC*. Based on the alignment of their amino acid sequences, CcTPS1 and CcTPS3 had the highest identity match, at 96.24% and 83.42%, with *α*-terpineol synthase (RWR83481.1) and another *α*-terpineol synthase (RWR97839.1) from *C. micranthum*, respectively. Interestingly, CcTPS4 was also annotated as TPS14—a linalool synthase expressed in *Arabidopsis thaliana* flowers—whose expression level was higher in borneol chemotypes than other chemical types of *C. camphora* in a previous study (Chen et al., 2018). However, predicting the function of the TPS gene based on sequence similarity alone is rather unpromising, since sequence homology between terpene synthase proteins is irrespective of their function (Hosoi et al., 2004; Landmann et al., 2007). Further, most TPSs are multi-product enzymes, which might be able to produce a mixed suite of 2 to 50 compounds (Kollner et al., 2004; Kulheim et al., 2015; Steele et al., 1998). Therefore, we suggested further research should be focused on characterizing the specific functioning of these three genes, as well as their TFs.

The GPPS, which catalyzes the formation of the universal precursor GPP for all monoterpenes, functions at the branch points of isoprenoid metabolism and fulfills a regulatory role in controlling the IPP flux into monoterpenes (Gershenzon & Croteau, 1993; Liang et al., 2002). The DEGs we detected in *C. camphora* only harbored one *GPPS*, while there were four DEGs annotated as GGPPS. This phenomenon was inconsistent with the results of metabolites, since GGPPSs were responsible for providing precursors for diterpene, which were undetected in our study. It is well known that plants’ GPPSs serve as homomeric and heteromeric structures (Burke, Klettke & Croteau, 2004; Rai et al., 2013; Wang & Dixon, 2009; Yin et al., 2017), and monoterpene biosynthesis in gymnosperms is mainly controlled by homomeric GPPS and small subunits (SSUs) of heteromeric GPPS (Schmidt et al., 2010; Tholl et al., 2004). Here, however, both SSUs and large subunits (LSUs) of heteromeric GPPS were frequently annotated as GGPPS in the KEGG database, probably due to their high homology with it. Therefore, unexpected additional GGPPS-related enzymes are often found within plant species, in which some functions as the LSU/SSU of heteromeric GPPS (Tholl et al., 2004; Wang & Dixon, 2009). For example, there are 12 annotated GGPPS paralogs in the *Arabidopsis* genome (Beck et al., 2013; Lange & Ghassemian, 2003). Yet after functional characterization, AtGPPS11 and AtGGPPS12 turn out to be LSU and SSU, respectively, and their interaction is involved in monoterpene biosynthesis, at least in *Arabidopsis* flowers (Chen, Fan & Wang, 2015). Among the tens of thousands of unigenes in *Tripterygium wilfordii*, eight were annotated as putative GGPPS. Nonetheless, a phylogenetic analysis of the known SC-PTSs revealed that GGPPS3 belongs to the GPPS.SSU type II subfamily (IDS-C3), while GGPPS6 was classified within the GPPS.SSU subfamily (IDS-C1), with GGPPS1 and GGPPS7 both assigned to the GGPS.LSU cluster; furthermore, this phylogenetic patterning was verified by functional characterization of those genes (Su et al., 2019; Zhang et al., 2015). Those findings suggested that phylogenetic analysis can be a powerful tool for predicting GPPS/GGPPS genes. Concerning our results, the CcGGPPS2, CcGGPPS3, and CcGGPPS4 clustered closely with the characterized GGPPS, while CcGGPPS1 fell into a GPPS small subunit type I subfamily clade (Fig. S2). CcGGPPS1 might interact with one of the CcGGPPS2, 3 or 4 to regulate monoterpene biosynthesis, offering a possible explanation for the high abundance of monoterpenes in *BCC*.

It is worth noting that the “plant hormone signal transduction” pathways, that are related to the JA signaling pathway as well as the alpha-linolenic acid metabolism pathway (it related to JA’s biosynthesis), were all significantly enriched in the CK vs. MD_2 h comparison or the CK vs. MD_6h comparison. Rapid accumulation of the phytohormone JA is generally considered an early response to MD or herbivory in most plants, which subsequently activates the JA signaling pathway to promote the expression of JA-responsive genes, such as those related to monoterpene biosynthesis (Chen et al., 2019; Menzel et al., 2014; Pratiwi et al., 2017; Schulze et al., 2019; Van Schie, Haring & Schuurink, 2007; Yoshitomi et al., 2016; Zhang et al., 2019). Therefore, by conducting a comprehensive analysis of genes related to JA biosynthesis and those involved in the JA signaling pathway, we identified 41 DEGs, of which 34 were putative JA metabolism pathway-related genes and the other 7 were JA signaling pathway-related unigenes. The expression patterns of these 41 unigenes appear in Figs. S3A–S3B and Table S16, which showed that almost all the unigenes were significantly induced by MD. Importantly, these patterns were also confirmed by qRT-PCR (Figs. S3C–3SD). Consistent with other plants’ response to MD, the JA signaling pathway probably plays a key role in regulating how *BCC* responds to the wounding of its leaves.

## Conclusions

The borneol chemotype of *Cinnamomum camphora*, a tree widely distributed in Southern China and is used in traditional Chinese medicine for thousands of years, is rich in D-borneol and other monoterpenes, such as camphor, eucalyptol, and bornyl acetate, to name a few. Although TFs (transcription factors) significantly contributed to regulating the expression of terpene synthase, their functions in *BCC* were largely unknown till now. We identified three monoterpene synthases (CcTPS1, CcTPS3, CcTPS4) which were likely responsible for the monoterpenoid biosynthesis in *BCC*. Notably, 37 TFs with complete ORFs and intact conserved domains, including five WRKY genes, 15 MYB genes, 10 ERF/AP2 genes, five bZIP genes, and two BHLH genes, were strongly and positively correlated (R^2^-values > 0.8) with two monoterpene synthase genes (*CcTPS1*, *CcTPS4*). Our study’s results provided insight into the genes participating in the biosynthesis and regulation of monoterpene in *BCC*, thereby laying the groundwork for further studies on the functional characterization of its candidate TPSs and TFs.

##  Supplemental Information

10.7717/peerj.11465/supp-1 Supplemental Information 1 Primers for the 23 selected unigenes used for qRT-PCR analysis

10.7717/peerj.11465/supp-2 Supplemental Information 2 Comparisons of the relative abundance of the identified volatile terpene among different treatment groups

10.7717/peerj.11465/supp-3 Supplemental Information 3 GO enrichment of up-regulated DEGs in CK vs MD_2h comparison Overrepresented BPs, MFs and CCs with P-values < 0.05 were identified. GO, Gene ontology. BPs, biological processes. MF, molecular functions. CC, cellular components.

10.7717/peerj.11465/supp-4 Supplemental Information 4 GO enrichment of up-regulated DEGs in CK vs MD_6h comparison Overrepresented BPs, MFs and CCs with P-values < 0.05 were identified. GO, Gene ontology. BPs, biological processes. MF, molecular functions. CC, cellular components.

10.7717/peerj.11465/supp-5 Supplemental Information 5 GO enrichment of up-regulated DEGs in MD_2h vs MD_6h comparison Overrepresented BPs, MFs and CCs with P-values < 0.05 were identified. GO, Gene ontology. BPs, biological processes. MF, molecular functions. CC, cellular components.

10.7717/peerj.11465/supp-6 Supplemental Information 6 KEGG enrichment of up-regulated DEGs in CK vs MD_2h comparison Overrepresented KEGG pathways with P-values < 0.05 were identified.

10.7717/peerj.11465/supp-7 Supplemental Information 7 KEGG enrichment of up-regulated DEGs in CK vs MD_6h comparison Overrepresented KEGG pathways with P-values < 0.05 were identified.

10.7717/peerj.11465/supp-8 Supplemental Information 8 KEGG enrichment of up-regulated DEGs in MD_2h vs MD_6h comparison Overrepresented KEGG pathways with P-values < 0.05 were identified.

10.7717/peerj.11465/supp-9 Supplemental Information 9 The expression data of DEGs related to the terpene biosynthesis

10.7717/peerj.11465/supp-10 Supplemental Information 10 Correlation analysis of terpenoid synthase and terpenoids

10.7717/peerj.11465/supp-11 Supplemental Information 11 The information of genes used for phylogenetic tree analysis

10.7717/peerj.11465/supp-12 Supplemental Information 12 The amino sequence of genes used for phylogenetic tree analysis

10.7717/peerj.11465/supp-13 Supplemental Information 13 Statistics of transcription factor families

10.7717/peerj.11465/supp-14 Supplemental Information 14 Expression data of the MYB, BHLH, WRKY, bZIP, AP2/ERF famlies

10.7717/peerj.11465/supp-15 Supplemental Information 15 The positive correlation of CcTPSs and CcTFs

10.7717/peerj.11465/supp-16 Supplemental Information 16 Expression analysis of the unigenes related to the JA biosynthesis and JA signaling pathway

10.7717/peerj.11465/supp-17 Supplemental Information 17 Raw data of the GC/MS and qRT-PCR

10.7717/peerj.11465/supp-18 Supplemental Information 18 Identification of the DEGs among three different groups A Volcano map showed the numbers of up- and down-regulation of DEGs between CK and MD_2h; B Volcano map showed the numbers of up- and down-regulation of DEGs between CK and MD_6h; C Volcano map showed the numbers of up- and down-regulation of DEGs between MD_2h and MD_6h; D A venn diagram showed the numbers of common and specific DEGs in three comparisons; E KEGG enrichment analysis of DEGs between CK and MD_2h; F KEGG enrichment analysis of DEGs between CK and MD_6h; G KEGG enrichment analysis of DEGs between MD_2h and MD_6h.

10.7717/peerj.11465/supp-19 Supplemental Information 19 Phylogenetic Analysis of Amino Acid Sequences of Homomeric and Heteromeric Plant GPPSs and GGPPSs GPPS/GGPPS identified in this study were shown in red square.

10.7717/peerj.11465/supp-20 Supplemental Information 20 Differential expression of the unigenes related to the JA biosynthesis and JA signaling pathway A Enzymes involved in biosynthesis and catabolism of JA; Enzymes identified in this study were shown in red frames, otherwise black frame. Enzymes with similar functions are listed in brackets. PLA1, phospholipase A1;DAD1, delayed anther dehiscence1; DGL and DALL, DAD1-LIKE lipase; LOX, lipoxygenase; AOS, allene oxide synthase; AOC, allene oxide cyclase; OPR3, OPDA reductase3; JMT, jasmonic acid carboxyl methyl transferase; OPR2, OPDA reductase2; JAR1, jasmonic resistant 1 (a JA amino acid conjugate synthase); cis- (+) -OPDA, cis- (+) -12-oxo-phytodienoic acid; OPC-8, 3-oxo-2- (2-pentenyl) -cyclopentane-1-octanoic acid; JA-Me, JA methyl ester; dnOPDA, dinor-OPDA; tnOPDA, tetranor-OPDA; 4,5-ddh-JA, 4,5-didehydro-JA; COI1: Coronatine-insensitive protein 1; JAZ: Jasmonate ZIM domain-containing protein; MYC2: Myelocytomatosis proteins; B Expression analysis of the unigenes related to the JA biosynthesis and JA signaling pathway; C qRT-PCR results of the 8 selected genes; D FPKM results of the 8 selected genes.

## Additional Information and Declarations

### DNA Deposition

The following information was supplied regarding the deposition of DNA sequences: All RNA-Seq raw data are available at CNGBdb: CNP0000956.

### Data Availability

The following information was supplied regarding data availability: The raw data of GC/MS analysis and qRT-PCR analysis are available in the Supplemental File.
