# Supplementary material for: Metabolism and transcriptome profiling provides insight into the genes and transcription factors involved in monoterpene biosynthesis of borneol chemotype of Cinnamomum camphora induced by mechanical damage
Source: PeerJ. 2021 Jul 1;9:e11465. doi: 10.7717/peerj.11465 (PMC8255067; doi:10.7717/peerj.11465)
Supplement: Supplemental Information 1 [file peerj-09-11465-s001.docx]

| Unigene ID | Short name | Forward primer | Reverse Primer |
| --- | --- | --- | --- |
| TRINITY_DN48358_c1_g1 | Ccdxs3 | ATGAACAATGCAGGGTATCTCG | TCAAAGCTCCAACAGGTGGTAT |
| TRINITY_DN49164_c2_g1 | Ccdxs5 | ATGAACAATGCGGGCTATCTGG | TCCCTCCTTTCTTGGAAATACCTTTA |
| TRINITY_DN36219_c0_g1 | CcGGPS1 | TGCGGACGATCAGAATCTCACG | ACACCAACACGCACCTCCAACT |
| TRINITY_DN47850_c3_g1 | CcGPS | TTCGTCCCACGGTGCTATTGTT | TACCACGTCTTGTATCTGCATCATCT |
| TRINITY_DN46420_c0_g3 | CcHMGR2 | CTCGGCTGTCTTTATTGCCACTG | CCCACCAACTGTACCCACCTCA |
| TRINITY_DN14566_c0_g1 | CcTPS1 | GGTTGCCTGTTCCCTCGACATT | CTCAGAGCCTGGAGCACCGAAT |
| TRINITY_DN43620_c0_g2 | CcTPS2 | GTGAGCCACAACTTAGTTATCC | ATTTCCCATCTGTCAACAGCAT |
| TRINITY_DN43620_c0_g4 | CcTPS3 | TTTGGTTATCCGAGGGTAGAGC | GTCCCATCTGTCAACAGCATCAGT |
| TRINITY_DN45508_c0_g2 | CcTPS4 | TTGGCAGTCCTATCAAATCCTC | TGAGTTCATCAGGTGTCCCATA |
| TRINITY_DN47700_c0_g1 | CcMYB84 | TGCTTCATCAGATTCTGTCGGTAC | AGCCACTCATCTTGCTGGGTTA |
| TRINITY_DN44045_c1_g7 | CcMYB88 | GCACCACCTCTGAGAAGTCCAT | GCCTGCTACTGTTGGCTAAGGA |
| TRINITY_DN45791_c4_g1 | CcWRKY2 | GTCCGTAAGCAGGTGCAAAGAT | TGGTTGAACAAGAAAGGAGCAT |
| TRINITY_DN47036_c3_g2 | CcbZIP18 | CGGCTTGAAGACGCTGTTATTG | GTGGTTCATAGCCATGCCGTAG |
| TRINITY_DN46575_c4_g1 | CcERF35 | TTGTAGGCAATGAGTGGAGTAGTGA | TCTGCGTCGAAAGACGGTAAGC |
| TRINITY_DN28254_c0_g2 | CcBHLH42 | TCATGCGCTTCTCGTCCTCGTC | CGACATCCTCAACAGCTCCGTCA |
| TRINITY_DN51210_c1_g1 | AOC | CCGTACAGGGACCGTATCTTAC | CAGCAACTCGGATGGCAACTCA |
| TRINITY_DN43748_c3_g1 | OPR | GGAGCCAATAGAGTTGGAATAA | CATGGGTAGAAGACTTTGAGGA |
| TRINITY_DN47495_c3_g8 | OPCL1 | GCCACCACATCCACCCTAGATTC | GGAGCCACCTGATAGCCCTTGTA |
| TRINITY_DN49017_c1_g5 | MFP2 | TTCACAGAACCGATAGACTTGG | ACACCAGCATATCCTCCATAAA |
| TRINITY_DN46183_c0_g6 | ACAA1 | TCATGGGCATTGGACCATCTATC | CTCCTCGGACGTTCAGCTTCTC |
| TRINITY_DN46358_c3_g3 | JMT | TTATTTCTCAAGTTTCGCTCAG | AACAGTCTCCACTAGAGGGTTC |
| TRINITY_DN50977_c1_g2 | JAR | TGGTCACCTACGTCGTGCTTCC | CTCTTCGCCAACTTTCACTTCC |
| TRINITY_DN47882_c0_g1 | MYC2 | CTCAACACCAGCAGACACTAACAGA | CGATTTCACTATGCCAGAAGAAGG |
