# Supplementary material for: Metabolism and transcriptome profiling provides insight into the genes and transcription factors involved in monoterpene biosynthesis of borneol chemotype of Cinnamomum camphora induced by mechanical damage
Source: PeerJ. 2021 Jul 1;9:e11465. doi: 10.7717/peerj.11465 (PMC8255067; doi:10.7717/peerj.11465)
Supplement: Supplemental Information 2 [file peerj-09-11465-s002.docx]

| Number | Compound name | RT/min | Formular | Mulecular Mass(g/mol) | Type | Content/% | | |
| --- | --- | --- | --- | --- | --- | --- | --- | --- |
|  |  |  |  |  |  | Ck | MD_2h | MD_6h |
| 1 | Carene | 9.57 | C10H16 | 136.125 | Mono | 0.05±0.01 | 0.04±0.01 | 0.06±0.01 |
| 2 | Pseudolimonen | 10.091 | C10H16 | 136.125 | Mono | 0.03±0.00 | 0.03±0.00 | 0.03±0.00 |
| 3 | Camphene | 10.154 | C10H16 | 136.125 | Mono | 0.02±0.00 | 0.02±0.00 | 0.02±0.00 |
| 4 | Sabinene | 10.457 | C10H16 | 136.125 | Mono | 0.02±0.00 | 0.02±0.00 | 0.02±0.00 |
| 5 | Phellandrene | 10.875 | C10H16 | 136.125 | Mono | 0.01±0.01 | 0.01±0.00 | 0.01±0.01 |
| 6 | L_Limonene | 11.332 | C10H16 | 136.125 | Mono | 0.01±0.00 | 0.01±0.00 | 0.01±0.00 |
| 7 | Eucalyptol | 11.481 | C10H18O | 154.136 | Mono | 0.02±0.00 | 0.02±0.00 | 0.03±0.00 |
| 8 | D_Limonene | 11.538 | C10H16 | 136.125 | Mono | 0.04±0.00 | 0.04±0.01 | 0.05±0.00 |
| 9 | Terpinolene | 12.311 | C10H16 | 136.125 | Mono | 0.01±0.00 | 0.01±0.01 | 0.01±0.01 |
| 10 | Camphor | 14.977 | C10H16O | 152.12 | Mono | 0.01±0.00 | 0.05±0.05 | 0.02±0.00 |
| 11 | Bornyl_acetate | 15.773 | C12H20O2 | 196.146 | Mono | 0.07±0.03 | 0.06±0.04 | 0.11±0.02 |
| 12 | L-Borneol | 16.19 | C10H18O | 154.136 | Mono | 0.01±0.00 | 0.01±0.00 | 0.01±0.00 |
| 13 | D-Borneol | 16.259 | C10H18O | 154.136 | Mono | 0.75±0. 11 | 0.87±0.12 | 1.02±0. 14 |
| 14 | Terpineol | 16.517 | C10H18O | 154.136 | Mono | 0.01±0.00 | 0.02±0.00 | 0.02±0.00 |
| 15 | Elemene | 17.106 | C15H24 | 204.188 | sesqui | 0.01±0.0 | 0.01±0.00 | 0.02±0.00 |
| 16 | germacreneB | 17.581 | C15H24 | 204.188 | sesqui | 0.01±0.00 | 0.02±0.00 | 0.02±0.00 |
| 17 | Caryophyllene | 17.947 | C15H24 | 204.188 | sesqui | 0.02±0.00 | 0.03±0.01 | 0.03±0.00 |
| 18 | Humulene | 18.451 | C15H24 | 204.188 | sesqui | 0.02±0..0 | 0.02±0.00 | 0.02±0.00 |
| 19 | β-Copaene | 18.714 | C15H24 | 204.188 | sesqui | 0.03±0.00 | 0.03±0.01 | 0.04±0.00 |
| 20 | Elemene_isomer | 18.84 | C15H24 | 204.188 | sesqui | 0.01±0.00 | 0.01±0.01 | 0.02±0.01 |
| 21 | β-selinene | 18.931 | C15H24 | 204.188 | sesqui | 0.02±0.00 | 0.03±0.01 | 0.04±0.01 |
