# Supplementary material for: Metabolism and transcriptome profiling provides insight into the genes and transcription factors involved in monoterpene biosynthesis of borneol chemotype of Cinnamomum camphora induced by mechanical damage
Source: PeerJ. 2021 Jul 1;9:e11465. doi: 10.7717/peerj.11465 (PMC8255067; doi:10.7717/peerj.11465)
Supplement: Supplemental Information 3 — Overrepresented BPs, MFs and CCs with P-values < 0.05 were identified. GO, Gene ontology. BPs, biological processes. MF, molecular functions. CC, cellular components. [file peerj-09-11465-s003.docx]

| **Category** | **GO ID** | **Description** | **p-value** | **gene Number** |
| --- | --- | --- | --- | --- |
| BP | GO:0048544 | recognition of pollen | 0.001 | 42 |
| BP | GO:0008037 | cell recognition | 0.001 | 42 |
| BP | GO:0044702 | single organism reproductive process | 0.001 | 45 |
| BP | GO:0022414 | reproductive process | 0.001 | 45 |
| BP | GO:0006351 | transcription, DNA-templated | 0.001 | 118 |
| BP | GO:0097659 | nucleic acid-templated transcription | 0.001 | 118 |
| BP | GO:0032774 | RNA biosynthetic process | 0.001 | 124 |
| BP | GO:0008643 | carbohydrate transport | 0.004 | 8 |
| BP | GO:0015074 | DNA integration | 0.047 | 47 |
| CC | GO:0031224 | intrinsic component of membrane | 0.001 | 1043 |
| CC | GO:0044425 | membrane part | 0.001 | 1095 |
| CC | GO:0016021 | integral component of membrane | 0.001 | 1039 |
| MF | GO:0004722 | protein serine/threonine phosphatase activity | 0.001 | 27 |
| MF | GO:0003700 | transcription factor activity, sequence-specific DNA binding | 0.001 | 99 |
| MF | GO:0001071 | nucleic acid binding transcription factor activity | 0.001 | 99 |
| MF | GO:0043531 | ADP binding | 0.001 | 56 |
| MF | GO:0030246 | carbohydrate binding | 0.001 | 68 |
| MF | GO:0004674 | protein serine/threonine kinase activity | 0.001 | 151 |
| MF | GO:0030594 | neurotransmitter receptor activity | 0.001 | 19 |
| MF | GO:0022824 | transmitter-gated ion channel activity | 0.001 | 19 |
| MF | GO:0022835 | transmitter-gated channel activity | 0.001 | 19 |
| MF | GO:0022834 | ligand-gated channel activity | 0.001 | 19 |
| MF | GO:0004970 | ionotropic glutamate receptor activity | 0.001 | 19 |
| MF | GO:0008066 | glutamate receptor activity | 0.001 | 19 |
| MF | GO:0015276 | ligand-gated ion channel activity | 0.001 | 19 |
| MF | GO:0005230 | extracellular ligand-gated ion channel activity | 0.001 | 19 |
| MF | GO:0016301 | kinase activity | 0.001 | 484 |
| MF | GO:0016773 | phosphotransferase activity, alcohol group as acceptor | 0.001 | 468 |
| MF | GO:0004672 | protein kinase activity | 0.001 | 429 |
| MF | GO:0003677 | DNA binding | 0.001 | 245 |
| MF | GO:0016772 | transferase activity, transferring phosphorus-containing groups | 0.001 | 522 |
| MF | GO:0004888 | transmembrane signaling receptor activity | 0.001 | 21 |
| MF | GO:0099600 | transmembrane receptor activity | 0.001 | 21 |
| MF | GO:0004553 | hydrolase activity, hydrolyzing O-glycosyl compounds | 0.001 | 89 |
| MF | GO:0005524 | ATP binding | 0.001 | 740 |
| MF | GO:0032559 | adenyl ribonucleotide binding | 0.001 | 771 |
| MF | GO:0001883 | purine nucleoside binding | 0.001 | 842 |
| MF | GO:0032555 | purine ribonucleotide binding | 0.001 | 842 |
| MF | GO:0032550 | purine ribonucleoside binding | 0.001 | 842 |
| MF | GO:0030554 | adenyl nucleotide binding | 0.001 | 772 |
| MF | GO:0017076 | purine nucleotide binding | 0.001 | 843 |
| MF | GO:0016740 | transferase activity | 0.001 | 842 |
| MF | GO:0032553 | ribonucleotide binding | 0.001 | 857 |
| MF | GO:0019787 | ubiquitin-like protein transferase activity | 0.001 | 41 |
| MF | GO:0032549 | ribonucleoside binding | 0.001 | 843 |
| MF | GO:0043168 | anion binding | 0.001 | 958 |
| MF | GO:0001882 | nucleoside binding | 0.001 | 844 |
| MF | GO:0097367 | carbohydrate derivative binding | 0.001 | 867 |
| MF | GO:0003824 | catalytic activity | 0.001 | 2140 |
| MF | GO:0035639 | purine ribonucleoside triphosphate binding | 0.001 | 811 |
| MF | GO:0000166 | nucleotide binding | 0.001 | 973 |
| MF | GO:1901265 | nucleoside phosphate binding | 0.001 | 973 |
| MF | GO:0004842 | ubiquitin-protein transferase activity | 0.001 | 39 |
| MF | GO:1901363 | heterocyclic compound binding | 0.001 | 1588 |
| MF | GO:0097159 | organic cyclic compound binding | 0.001 | 1588 |
| MF | GO:0036094 | small molecule binding | 0.001 | 982 |
| MF | GO:0016798 | hydrolase activity, acting on glycosyl bonds | 0.001 | 89 |
| MF | GO:0016705 | oxidoreductase activity, acting on paired donors, with incorporation or reduction of molecular oxygen | 0.002 | 86 |
| MF | GO:0043167 | ion binding | 0.004 | 1449 |
| MF | GO:0005506 | iron ion binding | 0.005 | 94 |
| MF | GO:0004872 | receptor activity | 0.005 | 32 |
| MF | GO:0060089 | molecular transducer activity | 0.005 | 32 |
| MF | GO:0038023 | signaling receptor activity | 0.005 | 32 |
| MF | GO:0004497 | monooxygenase activity | 0.005 | 69 |
| MF | GO:0020037 | heme binding | 0.010 | 106 |
| MF | GO:0033926 | glycopeptide alpha-N-acetylgalactosaminidase activity | 0.014 | 7 |
| MF | GO:0042578 | phosphoric ester hydrolase activity | 0.016 | 67 |
| MF | GO:0016757 | transferase activity, transferring glycosyl groups | 0.018 | 107 |
| MF | GO:0008081 | phosphoric diester hydrolase activity | 0.020 | 15 |
| MF | GO:0004012 | phospholipid-translocating ATPase activity | 0.023 | 9 |
| MF | GO:0005548 | phospholipid transporter activity | 0.023 | 9 |
| MF | GO:0005319 | lipid transporter activity | 0.034 | 9 |
| MF | GO:0016762 | xyloglucan:xyloglucosyl transferase activity | 0.035 | 10 |
| MF | GO:0004871 | signal transducer activity | 0.043 | 44 |
| MF | GO:0004721 | phosphoprotein phosphatase activity | 0.048 | 38 |
