# Supplementary material for: Metabolism and transcriptome profiling provides insight into the genes and transcription factors involved in monoterpene biosynthesis of borneol chemotype of Cinnamomum camphora induced by mechanical damage
Source: PeerJ. 2021 Jul 1;9:e11465. doi: 10.7717/peerj.11465 (PMC8255067; doi:10.7717/peerj.11465)
Supplement: Supplemental Information 4 — Overrepresented BPs, MFs and CCs with P-values < 0.05 were identified. GO, Gene ontology. BPs, biological processes. MF, molecular functions. CC, cellular components. [file peerj-09-11465-s004.docx]

| **Category** | **GO ID** | **Description** | **p-value** | **gene Number** |
| --- | --- | --- | --- | --- |
| BP | GO:0006351 | transcription, DNA-templated | 0.007 | 125 |
| BP | GO:0097659 | nucleic acid-templated transcription | 0.007 | 125 |
| BP | GO:0048544 | recognition of pollen | 0.007 | 34 |
| BP | GO:0008037 | cell recognition | 0.007 | 34 |
| BP | GO:0022414 | reproductive process | 0.019 | 36 |
| BP | GO:0044702 | single organism reproductive process | 0.019 | 35 |
| BP | GO:0042744 | hydrogen peroxide catabolic process | 0.027 | 24 |
| BP | GO:0042743 | hydrogen peroxide metabolic process | 0.027 | 24 |
| CC | GO:0005576 | extracellular region | 0.000 | 78 |
| CC | GO:0031224 | intrinsic component of membrane | 0.003 | 1056 |
| CC | GO:0016021 | integral component of membrane | 0.003 | 1053 |
| CC | GO:0044425 | membrane part | 0.016 | 1135 |
| MF | GO:0020037 | heme binding | 0.000 | 145 |
| MF | GO:0003824 | catalytic activity | 0.000 | 2443 |
| MF | GO:0016705 | oxidoreductase activity, acting on paired donors, with incorporation or reduction of molecular oxygen | 0.000 | 112 |
| MF | GO:0003700 | transcription factor activity, sequence-specific DNA binding | 0.000 | 98 |
| MF | GO:0001071 | nucleic acid binding transcription factor activity | 0.000 | 98 |
| MF | GO:0004497 | monooxygenase activity | 0.000 | 90 |
| MF | GO:0046906 | tetrapyrrole binding | 0.000 | 146 |
| MF | GO:0005506 | iron ion binding | 0.001 | 115 |
| MF | GO:0016491 | oxidoreductase activity | 0.002 | 704 |
| MF | GO:0043531 | ADP binding | 0.003 | 60 |
| MF | GO:0004553 | hydrolase activity, hydrolyzing O-glycosyl compounds | 0.004 | 99 |
| MF | GO:0016798 | hydrolase activity, acting on glycosyl bonds | 0.008 | 100 |
| MF | GO:0016773 | phosphotransferase activity, alcohol group as acceptor | 0.033 | 367 |
