# Supplementary material for: Metabolism and transcriptome profiling provides insight into the genes and transcription factors involved in monoterpene biosynthesis of borneol chemotype of Cinnamomum camphora induced by mechanical damage
Source: PeerJ. 2021 Jul 1;9:e11465. doi: 10.7717/peerj.11465 (PMC8255067; doi:10.7717/peerj.11465)
Supplement: Supplemental Information 5 — Overrepresented BPs, MFs and CCs with P-values < 0.05 were identified. GO, Gene ontology. BPs, biological processes. MF, molecular functions. CC, cellular components. [file peerj-09-11465-s005.docx]

| **Category** | **GO ID** | **Description** | **p-value** | **gene Number** |
| --- | --- | --- | --- | --- |
| BP | GO:0072593 | reactive oxygen species metabolic process | 0.001 | 26 |
| BP | GO:0042744 | hydrogen peroxide catabolic process | 0.001 | 24 |
| BP | GO:0042743 | hydrogen peroxide metabolic process | 0.001 | 24 |
| BP | GO:0006518 | peptide metabolic process | 0.001 | 301 |
| BP | GO:0006412 | translation | 0.001 | 298 |
| BP | GO:0043604 | amide biosynthetic process | 0.001 | 301 |
| BP | GO:0034645 | cellular macromolecule biosynthetic process | 0.001 | 385 |
| BP | GO:0043603 | cellular amide metabolic process | 0.001 | 308 |
| BP | GO:0043043 | peptide biosynthetic process | 0.001 | 298 |
| BP | GO:0009059 | macromolecule biosynthetic process | 0.001 | 389 |
| BP | GO:1901566 | organonitrogen compound biosynthetic process | 0.001 | 396 |
| BP | GO:0044271 | cellular nitrogen compound biosynthetic process | 0.002 | 421 |
| BP | GO:1901564 | organonitrogen compound metabolic process | 0.003 | 484 |
| BP | GO:1901576 | organic substance biosynthetic process | 0.004 | 548 |
| CC | GO:0005576 | extracellular region | 0.001 | 57 |
| CC | GO:0030529 | intracellular ribonucleoprotein complex | 0.001 | 339 |
| CC | GO:1990904 | ribonucleoprotein complex | 0.001 | 339 |
| CC | GO:0005840 | ribosome | 0.001 | 236 |
| CC | GO:0043232 | intracellular non-membrane-bounded organelle | 0.002 | 250 |
| CC | GO:0043228 | non-membrane-bounded organelle | 0.002 | 250 |
| MF | GO:0004497 | monooxygenase activity | 0.001 | 60 |
| MF | GO:0046906 | tetrapyrrole binding | 0.001 | 103 |
| MF | GO:0016705 | oxidoreductase activity, acting on paired donors, with incorporation or reduction of molecular oxygen | 0.001 | 72 |
| MF | GO:0005506 | iron ion binding | 0.001 | 76 |
| MF | GO:0020037 | heme binding | 0.001 | 103 |
| MF | GO:0005198 | structural molecule activity | 0.001 | 320 |
| MF | GO:0003735 | structural constituent of ribosome | 0.001 | 310 |
| MF | GO:0003746 | translation elongation factor activity | 0.001 | 43 |
| MF | GO:0016684 | oxidoreductase activity, acting on peroxide as acceptor | 0.001 | 41 |
| MF | GO:0004601 | peroxidase activity | 0.001 | 36 |
| MF | GO:0016491 | oxidoreductase activity | 0.001 | 422 |
| MF | GO:0004568 | chitinase activity | 0.009 | 8 |
| MF | GO:0004822 | isoleucine-tRNA ligase activity | 0.014 | 15 |
| MF | GO:0016209 | antioxidant activity | 0.017 | 52 |
| MF | GO:0016210 | naringenin-chalcone synthase activity | 0.022 | 4 |
| MF | GO:0010333 | terpene synthase activity | 0.033 | 8 |
| MF | GO:0008061 | chitin binding | 0.045 | 10 |
