# Supplementary material for: Metabolism and transcriptome profiling provides insight into the genes and transcription factors involved in monoterpene biosynthesis of borneol chemotype of Cinnamomum camphora induced by mechanical damage
Source: PeerJ. 2021 Jul 1;9:e11465. doi: 10.7717/peerj.11465 (PMC8255067; doi:10.7717/peerj.11465)
Supplement: Supplemental Information 6 — Overrepresented KEGG pathways with P-values < 0.05 were identified. [file peerj-09-11465-s006.docx]

| **First Category** | **Second Category** | **Pathway id** | **Description** | **P_value** | **Num** |
| --- | --- | --- | --- | --- | --- |
| Organismal Systems | Environmental adaptation | map04626 | Plant-pathogen interaction | 0.000 | 91 |
| Environmental Information Processing | Signal transduction | map04075 | Plant hormone signal transduction | 0.000 | 53 |
| Metabolism | Biosynthesis of other secondary metabolites | map00940 | Phenylpropanoid biosynthesis | 0.000 | 47 |
| Metabolism | Lipid metabolism | map00592 | alpha-Linolenic acid metabolism | 0.000 | 25 |
| Cellular Processes | Transport and catabolism | map04144 | Endocytosis | 0.001 | 103 |
| Environmental Information Processing | Signal transduction | map04016 | MAPK signaling pathway - plant | 0.001 | 52 |
| Metabolism | Lipid metabolism | map00564 | Glycerophospholipid metabolism | 0.005 | 46 |
| Metabolism | Carbohydrate metabolism | map00520 | Amino sugar and nucleotide sugar metabolism | 0.010 | 67 |
| Metabolism | Biosynthesis of other secondary metabolites | map00232 | Caffeine metabolism | 0.014 | 4 |
| Metabolism | Carbohydrate metabolism | map00052 | Galactose metabolism | 0.013 | 36 |
| Metabolism | Metabolism of other amino acids | map00440 | Phosphonate and phosphinate metabolism | 0.020 | 7 |
| Environmental Information Processing | Membrane transport | map02010 | ABC transporters | 0.030 | 16 |
| Environmental Information Processing | Signal transduction | map04070 | Phosphatidylinositol signaling system | 0.043 | 22 |
