# Supplementary material for: Metabolism and transcriptome profiling provides insight into the genes and transcription factors involved in monoterpene biosynthesis of borneol chemotype of Cinnamomum camphora induced by mechanical damage
Source: PeerJ. 2021 Jul 1;9:e11465. doi: 10.7717/peerj.11465 (PMC8255067; doi:10.7717/peerj.11465)
Supplement: Supplemental Information 7 — Overrepresented KEGG pathways with P-values < 0.05 were identified. [file peerj-09-11465-s007.docx]

| **First Category** | **Second Category** | **Pathway id** | **Description** | **P_value** | **Num** |
| --- | --- | --- | --- | --- | --- |
| Metabolism | Biosynthesis of other secondary metabolites | map00940 | Phenylpropanoid biosynthesis | 0.000 | 68 |
| Metabolism | Lipid metabolism | map00592 | alpha-Linolenic acid metabolism | 0.000 | 31 |
| Organismal Systems | Environmental adaptation | map04626 | Plant-pathogen interaction | 0.000 | 97 |
| Metabolism | Amino acid metabolism | map00400 | Phenylalanine, tyrosine and tryptophan biosynthesis | 0.013 | 32 |
| Metabolism | Metabolism of other amino acids | map00450 | Selenocompound metabolism | 0.015 | 23 |
| Metabolism | Amino acid metabolism | map00270 | Cysteine and methionine metabolism | 0.012 | 75 |
| Metabolism | Amino acid metabolism | map00350 | Tyrosine metabolism | 0.007 | 57 |
| Environmental Information Processing | Signal transduction | map04016 | MAPK signaling pathway - plant | 0.011 | 57 |
| Metabolism | Amino acid metabolism | map00360 | Phenylalanine metabolism | 0.008 | 37 |
| Metabolism | Biosynthesis of other secondary metabolites | map00945 | Stilbenoid, diarylheptanoid and gingerol biosynthesis | 0.010 | 11 |
| Metabolism | Metabolism of terpenoids and polyketides | map00902 | Monoterpenoid biosynthesis | 0.020 | 6 |
| Cellular Processes | Transport and catabolism | map04144 | Endocytosis | 0.007 | 118 |
| Metabolism | Lipid metabolism | map00071 | Fatty acid degradation | 0.032 | 70 |
| Metabolism | Metabolism of other amino acids | map00410 | beta-Alanine metabolism | 0.042 | 49 |
