# Supplementary material for: Metabolism and transcriptome profiling provides insight into the genes and transcription factors involved in monoterpene biosynthesis of borneol chemotype of Cinnamomum camphora induced by mechanical damage
Source: PeerJ. 2021 Jul 1;9:e11465. doi: 10.7717/peerj.11465 (PMC8255067; doi:10.7717/peerj.11465)
Supplement: Supplemental Information 8 — Overrepresented KEGG pathways with P-values < 0.05 were identified. [file peerj-09-11465-s008.docx]

| **First Category** | **Second Category** | **Pathway id** | **Description** | **P_value** | **Num** |
| --- | --- | --- | --- | --- | --- |
| Genetic Information Processing | Translation | map03010 | Ribosome | 0.000 | 363 |
| Metabolism | Biosynthesis of other secondary metabolites | map00940 | Phenylpropanoid biosynthesis | 0.000 | 42 |
| Organismal Systems | Environmental adaptation | map04626 | Plant-pathogen interaction | 0.000 | 61 |
| Environmental Information Processing | Signal transduction | map04016 | MAPK signaling pathway - plant | 0.000 | 43 |
| Metabolism | Biosynthesis of other secondary metabolites | map00941 | Flavonoid biosynthesis | 0.000 | 12 |
| Environmental Information Processing | Signal transduction | map04075 | Plant hormone signal transduction | 0.000 | 36 |
| Metabolism | Metabolism of other amino acids | map00450 | Selenocompound metabolism | 0.001 | 17 |
| Metabolism | Biosynthesis of other secondary metabolites | map00945 | Stilbenoid, diarylheptanoid and gingerol biosynthesis | 0.001 | 9 |
| Metabolism | Carbohydrate metabolism | map00010 | Glycolysis / Gluconeogenesis | 0.002 | 92 |
| Metabolism | Amino acid metabolism | map00380 | Tryptophan metabolism | 0.003 | 45 |
| Metabolism | Lipid metabolism | map00592 | alpha-Linolenic acid metabolism | 0.005 | 15 |
| Metabolism | Amino acid metabolism | map00350 | Tyrosine metabolism | 0.004 | 34 |
| Metabolism | Lipid metabolism | map00073 | Cutin, suberine and wax biosynthesis | 0.005 | 6 |
| Metabolism | Metabolism of terpenoids and polyketides | map00902 | Monoterpenoid biosynthesis | 0.005 | 5 |
| Metabolism | Lipid metabolism | map00071 | Fatty acid degradation | 0.006 | 43 |
| Metabolism | Amino acid metabolism | map00360 | Phenylalanine metabolism | 0.009 | 22 |
| Metabolism | Amino acid metabolism | map00270 | Cysteine and methionine metabolism | 0.010 | 43 |
| Metabolism | Metabolism of terpenoids and polyketides | map00904 | Diterpenoid biosynthesis | 0.008 | 6 |
| Metabolism | Metabolism of terpenoids and polyketides | map00903 | Limonene and pinene degradation | 0.010 | 18 |
| Metabolism | Carbohydrate metabolism | map00620 | Pyruvate metabolism | 0.014 | 61 |
| Organismal Systems | Environmental adaptation | map04712 | Circadian rhythm - plant | 0.015 | 9 |
| Metabolism | Carbohydrate metabolism | map00053 | Ascorbate and aldarate metabolism | 0.018 | 22 |
| Metabolism | Lipid metabolism | map00561 | Glycerolipid metabolism | 0.023 | 38 |
| Genetic Information Processing | Translation | map03013 | RNA transport | 0.030 | 71 |
| Metabolism | Amino acid metabolism | map00340 | Histidine metabolism | 0.032 | 21 |
| Metabolism | Biosynthesis of other secondary metabolites | map00943 | Isoflavonoid biosynthesis | 0.030 | 2 |
