# Supplementary material for: Metabolism and transcriptome profiling provides insight into the genes and transcription factors involved in monoterpene biosynthesis of borneol chemotype of Cinnamomum camphora induced by mechanical damage
Source: PeerJ. 2021 Jul 1;9:e11465. doi: 10.7717/peerj.11465 (PMC8255067; doi:10.7717/peerj.11465)
Supplement: Supplemental Information 9 [file peerj-09-11465-s009.docx]

| **Gene_id** | **Rename** | **Expression** | | | **Description** | **E_value** | **Identity** | **Accession number** |
| --- | --- | --- | --- | --- | --- | --- | --- | --- |
|  |  | **CK** | **MD_2h** | **MD_6h** |  |  |  |  |
| TRINITY_DN44951_c0_g1 | Ccdxs1 | 1.30 | 0.49 | 0.10 | 1-deoxy-D-xylulose 5-phosphate synthase 1 [Cinnamomum micranthum f. kanehirae] | 0.0 | 99.05% | RWR77329.1 |
| TRINITY_DN44951_c0_g2 | Ccdxs2 | 0.59 | 0.25 | 8.82 | 1-deoxy-D-xylulose 5-phosphate synthase 1 [Cinnamomum micranthum f. kanehirae] | 0.0 | 96.92% | RWR83151.1 |
| TRINITY_DN48358_c1_g1 | Ccdxs3 | 40.20 | 85.87 | 49.94 | putative 1-deoxy-D-xylulose-5-phosphate synthase, chloroplastic [Cinnamomum micranthum f. kanehirae] | 0.0 | 99.83% | RWR93643.1 |
| TRINITY_DN49164_c0_g1 | Ccdxs4 | 0.25 | 1.14 | 0.56 | 1-deoxy-D-xylulose 5-phosphate synthase 2 [Cinnamomum micranthum f. kanehirae] | 0.0 | 99.18% | RWR84357.1 |
| TRINITY_DN49164_c2_g1 | Ccdxs5 | 9.67 | 83.20 | 60.65 | 1-deoxy-D-xylulose 5-phosphate synthase 2 [Cinnamomum micranthum f. kanehirae] | 0.0 | 98.82% | RWR75348.1 |
| TRINITY_DN39516_c1_g1 | CcFDPS1 | 0.02 | 1.09 | 1.83 | ERG20 farnesyl diphosphate synthase [Rhizodiscina lignyota] | 0.0 | 83.57% | KAF2094352.1 |
| TRINITY_DN45005_c1_g3 | CcFDPS2 | 13.56 | 25.07 | 51.15 | fanesyl diphosphate synthase 1 [Cinnamomum micranthum f. kanehirae] | 0.0 | 98.70% | RWR95015.1 |
| TRINITY_DN36219_c0_g1 | CcGGPS1 | 32.03 | 23.77 | 90.69 | terpenoid synthase [Rhizodiscina lignyota] | 0.0 | 80.08% | KAF2104017.1 |
| TRINITY_DN44912_c1_g3 | CcGGPS2 | 27.11 | 13.41 | 19.63 | geranylgeranyl pyrophosphate synthase 1 [Cinnamomum micranthum f. kanehirae] | 0.0 | 98.45% | RWR74263.1 |
| TRINITY_DN48302_c1_g7 | CcGGPS3 | 3.53 | 25.98 | 75.88 | geranylgeranyl diphosphate synthase 3 [Cinnamomum micranthum f. kanehirae] | 0.0 | 98.43% | RWR95691.1 |
| TRINITY_DN48513_c1_g3 | CcGGPS4 | 0.00 | 0.44 | 0.69 | geranylgeranyl pyrophosphate synthase 1 [Cinnamomum camphora] | 0.0 | 100.00% | ATT59265.1 |
| TRINITY_DN47850_c3_g1 | CcGPS | 10.16 | 24.10 | 15.54 | solanesyl-diphosphate synthase 1, mitochondrial isoform X1 [Cinnamomum micranthum f. kanehirae] | 0.0 | 93.93% | RWR85838.1 |
| TRINITY_DN46420_c0_g2 | CcHMGR1 | 2.76 | 56.95 | 8.27 | 3-hydroxy-methylglutaryl coenzyme A reductase [Cinnamomum micranthum f. kanehirae] | 0.0 | 98.54% | RWR72749.1 |
| TRINITY_DN46420_c0_g3 | CcHMGR2 | 36.58 | 110.55 | 163.41 | 3-hydroxy-methylglutaryl coenzyme A reductase [Cinnamomum micranthum f. kanehirae] | 0.0 | 99.36% | RWR78307.1 |
| TRINITY_DN38569_c0_g2 | CcHMGR3 | 0.02 | 0.55 | 0.77 | 3-hydroxy-3-methylglutaryl-coenzyme A reductase [Rhizodiscina lignyota] | 0.0 | 84.64% | KAF2093630.1 |
| TRINITY_DN40020_c0_g3 | CcHMGS1 | 0.05 | 0.57 | 1.18 | hydroxymethylglutaryl-CoA synthase [Rhizodiscina lignyota] | 0.0 | 80.98% | KAF2097464.1 |
| TRINITY_DN40020_c0_g4 | CcHMGS2 | 0.00 | 0.82 | 0.95 | Hydroxymethylglutaryl-CoA synthase [Alternaria arborescens] | 0.0 | 92.31% | XP_028504853.1 |
| TRINITY_DN45989_c1_g3 | CcHMGS3 | 13.33 | 61.94 | 23.94 | hydroxymethylglutaryl-CoA synthase-like protein isoform X1 [Cinnamomum micranthum f. kanehirae] | 0.0 | 92.79% | RWR80718.1 |
| TRINITY_DN37108_c0_g1 | CcIDI1 | 0.00 | 0.24 | 1.17 | hypothetical protein M409DRAFT_24925 [Zasmidium cellare ATCC 36951] | 0.0 | 84.51% | KAF2165025.1 |
| TRINITY_DN38184_c0_g2 | CcIDI2 | 0.00 | 1.05 | 1.35 | isopentenyl-diphosphate delta isomeras-like protein 1 [Rhizodiscina lignyota] | 0.0 | 81.89% | KAF2103112.1 |
| TRINITY_DN14566_c0_g1 | CcTPS1 | 0.17 | 0.18 | 32.34 | Alpha-terpineol synthase, chloroplastic [Cinnamomum micranthum f. kanehirae] | 0.0 | 96.24% | RWR83481.1 |
| TRINITY_DN43620_c0_g2 | CcTPS2 | 0.68 | 0.42 | 21.35 | terpene synthase 10-like protein isoform X1 [Cinnamomum micranthum f. kanehirae] | 0.0 | 98.66% | RWR88217.1 |
| TRINITY_DN43620_c0_g4 | CcTPS3 | 2.21 | 1.19 | 0.24 | alpha-terpineol synthase [Cinnamomum micranthum f. kanehirae] | 0.0 | 83.42% | RWR97839.1 |
| TRINITY_DN45508_c0_g2 | CcTPS4 | 0.77 | 12.26 | 62.09 | S-+-linalool synthase [Cinnamomum micranthum f. kanehirae] | 0.0 | 97.49% | RWR97874.1 |
| TRINITY_DN47188_c1_g1 | CcTPS5 | 2.87 | 9.17 | 40.31 | Short-chain dehydrogenase/reductase SDR [Cinnamomum micranthum f. kanehirae] | 0.0 | 100.00% | RWR72159.1 |
| TRINITY_DN49732_c0_g3 | CcTPS6 | 60.57 | 234.16 | 196.62 | S-+-linalool synthase [Cinnamomum micranthum f. kanehirae] | 0.0 | 94.39% | RWR93550.1 |
| TRINITY_DN50510_c2_g4 | CcTPS7 | 18.05 | 17.58 | 60.87 | Short-chain dehydrogenase/reductase SDR [Cinnamomum micranthum f. kanehirae] | 0.0 | 95.83% | RWR72160.1 |
| TRINITY_DN40799_c0_g1 | CcTPS8 | 2.20 | 1.33 | 0.04 | terpene synthase 1 [Cinnamomum micranthum f. kanehirae] | 0.0 | 98.20% | RWR93513.1 |
| TRINITY_DN46911_c0_g1 | CcTPS9 | 1.99 | 6.42 | 0.29 | terpene synthase 1 [Cinnamomum micranthum f. kanehirae] | 0.0 | 86.27% | RWR93512.1 |
| TRINITY_DN46911_c3_g2 | CcTPS10 | 1.75 | 1.65 | 0.31 | terpene synthase 1 [Cinnamomum micranthum f. kanehirae] | 0.0 | 98.34% | RWR88240.1 |
| TRINITY_DN47476_c0_g1 | CcTPS11 | 0.81 | 0.57 | 8.52 | diterpene geranyllinalool synthase [Cinnamomum micranthum f. kanehirae] | 0.0 | 97.34% | RWR96469.1 |
| TRINITY_DN47476_c0_g2 | CcTPS12 | 1.73 | 0.63 | 15.55 | diterpene geranyllinalool synthase [Cinnamomum micranthum f. kanehirae] | 0.0 | 97.88% | RWR96468.1 |
| TRINITY_DN49635_c3_g3 | CcTPS13 | 1.73 | 1.19 | 0.13 | ent-copalyl diphosphate synthase, chloroplastic-like protein [Cinnamomum micranthum f. kanehirae] | 0.0 | 98.15% | RWR73750.1 |
| TRINITY_DN51000_c1_g1 | CcTPS14 | 12.14 | 15.69 | 26.44 | diterpene geranyllinalool synthase [Cinnamomum micranthum f. kanehirae] | 0.0 | 82.24% | RWR88024.1 |
