# Supplementary material for: Metabolism and transcriptome profiling provides insight into the genes and transcription factors involved in monoterpene biosynthesis of borneol chemotype of Cinnamomum camphora induced by mechanical damage
Source: PeerJ. 2021 Jul 1;9:e11465. doi: 10.7717/peerj.11465 (PMC8255067; doi:10.7717/peerj.11465)
Supplement: Supplemental Information 10 [file peerj-09-11465-s010.docx]

| **TPS** | **Terpenes** | **Correlation coefficient** | **P_value** |
| --- | --- | --- | --- |
| CcTPS1 | Carene | 0.803 | 0.009 |
| CcTPS1 | Pseudolimonen | 0.795 | 0.01 |
| CcTPS1 | Camphene | 0.837 | 0.005 |
| CcTPS1 | Sabinene | 0.787 | 0.012 |
| CcTPS1 | L_Limonene | 0.778 | 0.014 |
| CcTPS1 | D_Limonene | 0.77 | 0.015 |
| CcTPS3 | Camphor | 0.667 | 0.05 |
| CcTPS3 | D-borneol | 0.767 | 0.016 |
| CcTPS3 | Terpineol | 0.733 | 0.025 |
| CcTPS4 | Bornyl acetate | 0.683 | 0.042 |
| CcTPS4 | D-borneol | 0.767 | 0.016 |
| CcTPS4 | Terpineol | 0.7 | 0.036 |
