# Supplementary material for: Metabolism and transcriptome profiling provides insight into the genes and transcription factors involved in monoterpene biosynthesis of borneol chemotype of Cinnamomum camphora induced by mechanical damage
Source: PeerJ. 2021 Jul 1;9:e11465. doi: 10.7717/peerj.11465 (PMC8255067; doi:10.7717/peerj.11465)
Supplement: Supplemental Information 11 [file peerj-09-11465-s011.docx]

| **Subfamlily** | **TPS name** | **Origin** | **Function** | **Accession number** |
| --- | --- | --- | --- | --- |
|  |  |  |  |  |
| TPSb | Ms 4S-limonene syn | Mentha spicata | 4S-limonene synthase | AAC37366.1 |
|  | St (+)-4R-limonene syn | Schizonepeta tenuifolia | (+)-4R-limonene synthasethase | AAG01140.2 |
|  | Pf limonene syn | Perilla frutescens var. acuta | limonene synthase | BAA08367.1 |
|  | Pf mercene syn | Perilla frutescens var. acuta | mercene synthase | AAF76186.1 |
|  | St 3-carene syn | Salvia stenophylla | 3-carene synthase | AAM89254.1 |
|  | So 1.8-cineole syn | Salvia officinalis | 1.8-cineole synthase | AAC26016.1 |
|  | So (+)-bornyl diphosphate syn | Salvia officinalis | (+)-bornyl diphosphate synthase | (AAC26017.1 |
|  | So (+)-sabinene syn | Salvia officinalis | (+)-sabinene synthase | AAC26018.1 |
|  | La bornyl diphosphate syn | Lavandula angustifolia subsp. angustifolia | bornyl diphosphate synthase | AJW68082.1 |
|  | Pd bornyl diphosphate syn | Phyla dulcis | bornyl diphosphate synthase | ATY48638.1 |
|  | Pd linalool syn | Phyla dulcis | linalool synthase | ATY48639.1 |
|  | At 1.8-cineole syn | Arabidopsis thaliana | 1.8-cineole synthase | NP_189212.1 |
|  | At myrcene syn | Arabidopsis thaliana | myrcene synthase | NP_179998.1 |
|  | CcTPS1 | Borneol Chemotype of *Cinnamomum camphora* | CcTPS1 | / |
|  | CcTPS3 | Borneol Chemotype of *Cinnamomum camphora* | CcTPS3 | / |
|  | Mg terpineol syn | Magnolia grandiflora | terpineol synthase | B3TPQ7.1 |
|  | Cu (E)-beta-ocimene syn | Citrus unshiu | (E)-beta-ocimene synthase | BAD91046.1 |
|  | Cl (-)-beta-pinene syn | Citrus limon | (-)-beta-pinene synthase | AAM53945.1 |
|  | Cl terpinene syn | Citrus limon | terpinene synthase | AAM53943.1 |
|  | Wv bornyl diphosphate syn | Wurfbainia villosa | bornyl diphosphate synthase | AWW87313.1 |
|  | Wv pinene syn | Wurfbainia villosa | pinene synthase | AWW87312.1 |
| TPSa | Ob selinene syn | Ocimum basilicum | selinene synthase | AAV63785.1 |
|  | CcTPS8 | Borneol Chemotype of *Cinnamomum camphora* | CcTPS8 | / |
|  | CcTPS9 | Borneol Chemotype of *Cinnamomum camphora* | CcTPS9 | / |
|  | Mp (E)-B-farnesene syn | Mentha x piperita | (E)-B-farnesene synthase | AAB95209.1 |
|  | Na 5-epi-aristolochene syn | Nicotiana attenuata | 5-epi-aristolochene synthase | AAO85555.1 |
| TPSd | Pt farnesene syn | Pinus taeda | farnesene synthase | AAO61226.1 |
|  | Pa_(-)-limonene_syn | Picea abies | (-)-limonene_synthase | AAS47694.1 |
|  | Pa_(-)-pinene_syn | Picea abies | (-)-pinene_synthase | AAS47692.1 |
| TPSg | CbTPS3 | Cinnamomum burmanni | CbTPS3 | / |
|  | Cm_S-(+)-linalool_syn | Cinnamomum micranthum f. kanehirae | S-(+)-linaloolsynthase | AFK09262.1 |
|  | CcTPS4 | Borneol Chemotype of *Cinnamomum camphora* | CcTPS4 |  |
|  | CcTPS6 | Borneol Chemotype of *Cinnamomum camphora* | CcTPS6 |  |
|  | Am_(E)-b-ocimene_syn | Antirrhinum majus | (E)-b-ocimene synthase | AAO42614.1 |
|  | At_linalool_syn | Arabidopsis thaliana | linalool synthase | AAO85533.1 |
|  | Am_myrcene_syn | Antirrhinum majus | myrcene_synthase | AAO41727.1 |
|  | Am_nerolidol/linalool_syn | Antirrhinum majus | nerolidol/linalool_synthase | ABR24417.1 |
| TPSe | Cm_ent-kaurene_syn | Cucurbita maxima | ent-kaurene_synthase | AAB39482.1 |
|  | Tw_kaurene_syn | Tripterygium wilfordii | kaurene_synthase | KX931055 |
|  | Tw_kaurene_syn | Tripterygium wilfordii | kaurene_synthase | AKM28415 |
| TPSf | Cb_linalool_syn | Clarkia breweri | linalool_synthase | AAD19840.1 |
|  | Cc_linalool_syn | Clarkia concinna | linalool_synthase | AAD19839.1 |
|  | CcTPS12 | Borneol Chemotype of *Cinnamomum camphora* | CcTPS12 | / |
|  | CcTPS13 | Borneol Chemotype of *Cinnamomum camphora* | CcTPS13 | / |
|  | CcTPS14 | Borneol Chemotype of *Cinnamomum camphora* | CcTPS14 | / |
| TPSc | Cm_copalyl_diphosphate_syn | Cucurbita maxima | copalyl_diphosphate_synthase | AAD04292.1 |
|  | Sl_copalyl_diphosphate_syn | Solanum lycopersicum | copalyl_diphosphate_synthase | BAA84918.1 |
|  | Tw_copalyl_diphosphate_syn1 | Tripterygium wilfordii | copalyl_diphosphate_synthase | AKM28412 |
|  | Tw_copalyl_diphosphate_syn2 | Tripterygium wilfordii | copalyl_diphosphate_synthase | AKM28413 |
|  | CcTPS11 | Borneol Chemotype of *Cinnamomum camphora* | CcTPS11 | / |
