# Supplementary material for: Metabolism and transcriptome profiling provides insight into the genes and transcription factors involved in monoterpene biosynthesis of borneol chemotype of Cinnamomum camphora induced by mechanical damage
Source: PeerJ. 2021 Jul 1;9:e11465. doi: 10.7717/peerj.11465 (PMC8255067; doi:10.7717/peerj.11465)
Supplement: Supplemental Information 12 [file peerj-09-11465-s012.docx]

>CcTPS1

MSLNLVSPSFPCSLVRLFSLVSDHAPSLSYLKIEHVPLNPKARSKRNAAPPRKCALRAST

LETDVARRSANYSPTVWDFDFIQSLTSAYKDGAYTRRVEELKNYVRSLLLDSSAPLARVE

LINHLQRLGVGYLFGEEIKTVLDTIGKGKDFGMEKDLNATALQFRILRQNGYYASKEVFN

SFIDEMGSFKACLCEDTKGLLSLYEASYLAFPGETIMDEAKAFARRHLKNLKGEIDPRLE

EQVAHALELPTHYRMLRLEARWYIDMYEKEESMDSLILELAKLDYNILQASYQKDVQNGY

RWWRQLGLTEKLPFTRDRWLECYLFSLSITFEPQYGYGREVLNKVNQMITTIDDIYDVYG

TVEELELFTDAVARWDTSVIQQLPEYMKTCFLALLNFGNDLAYDTLKEQGYDIIPYLRKL

WADLCKAYLVEARWYHNGYAPTLEEYLRNAWISISGPVVLVHGYFSMRLKITKEVLQGIE

NYADLIRFSSMILRLCDDMGTSTHELERGDVLKSIQCYMHEANVSEAIAREHIRSLADET

WKKMNKEYVTGCLFPRHFADAAIGLIRRAESVYHKGDGFGAPGSEIDGQVTSLVVEPIVI

NNNGINMGSVI

>CcTPS3

MALSTVSTFPCLKHPSFFLNVFHSSTKNFLSATKVVRPVNCIPNSVIYEPEVSRRKANYK

PNIWDHDLLQSLRSDYQDEAYVKRAEKLKEEINCLLQEAVNSLAQLEMIDAIERLGLGHI

FDKKIKEVLNTMWVSHNNNENKGGTENKDLYATSLLFRLLRKHGYSVSQDVFNKFMDEKG

GLNARVCEDIKGILSLYEASHFAHQGETLLSECRTFTWMYLKAFKGSGDIILANKVEHAL

ELPMHWRIGRMEALWHLNMYESVEHMNPTLLELAKLDFNMVQATHQRDLRKASRWWRSIG

LGGKLSFSRDRMMECFFVALGVMSEPQFGYPRVELAKVCQLITTIDDIYDVFGSLDELEC

FTDAVDRWDIKSIDLLPEYMKICFLALYNTTNEMGYEILKDQGINIIPYLQKGWTDFCKA

MLVESKWYYSGYKPSLEEYLNNGWVSSSGPVILVHAFLLSKQPISTQVLDGLDKNPGLIR

WPSMIFRLCNDLATYKDEKVRGDAPSSIDCYMKEANVSEMDARNHVEDLIFNSWKKLNEE

VRTVSPYPPHFINCALNLARVVHCIYQHGDGHTVQDRNTKDRLTSLLVRPIPLIVK

>CcTPS4

MAFCSRLMAFSSFPINPSKVQSFGRSRITVPQVASGHWQRSTQDPDMDTLSIKCREKINQ

VRHILQNLEDPSGRLVMIDHLQRLGIDHHFPEEIESFISSEYNNLHSGNNGGNGSSIYDV

SLTFRLLREHGYYVSSDIFNNFKDEEGSFKPSLGKDIKGMVGLYEASEVAIQGEDILDEA

NDFAKKSLFASLTSVESSQTSIVRHALENPFHMSMPRFSTKHHLKNLKGNDVNTKSLQEL

AKLDFNIVQLMHQDELKQVSKWWRDLGLSQELKFARDQPLKWFMWPLAVLSNPQFSKYRV

ELTKPISFIYIIDDIFDVYGTPDELILFTEAVNRWDPSDIDQLPRYMKICFMALYNLTNE

IAFMVLKEHGWNPIDSLRKSWTDLCNAFLVESKWFASGHLPKADEYLRNGVTSSGVHIVL

VHIFFLVGHGITRNGIDSVDNIPGLISFPATILRLWDDLGSAKDENQEGYDGSYVDCYMK

EHGTSLESARQHVRQMISNAWKGLNKECLSPNPFSQSFVNASLNTARMVQVMYSYDKDQR

LPVLEQFISTLLKESIPL

>CcTPS6

MQSSVASTVLSLPKCPSSMKEDQNHEKAENSVLQVATFCCQSSTQDQLSTSSSLSSDGLS

IEHVEKVNQVTAIVQNLKEPSERLVMIDDLRRLGIEYHFQEEIESLLCGLCENYGALSSL

YDVALSFRLLREHGHNVSQDVFKRFMDEEGRFKLQLSTDIKGMMSLYEASKLAVKGEDII

EEANDFATKNLIASVKFMEPCLARVVRHSLENPFHMSLPRFNTKNHLNNLRETDRNTEAI

QELAIFEFNIVQSMHQSELKEVTQWWRDLGLSQELRFARDQPLKWYMWSLAVLPNPKFSK

YRIELTKPIALVYIIDDIYDVYGKPDELVLFTEAINRWDPSDISQLPRYMKICFMALHSV

TNELAYMVLKEHGWNPINSFRQKWRDLCNAFLVEAKWFAEGDLPKADEYLRNGVTSSGVA

AVLVHLFFLVGNGITRESVDLVDSIPKLISCPAMILRLWDDLGSAKDENQDGYDGSYLEC

YMKENAASLESTQRHVRHLISNAWKELNKESLSPYPFSQTFVQASLNTARMVQVMYSYNK

DQRLPMLEQHIISLLNERIPLDEIPKKAQR

>CcTPS8

MALVSGSGHSDGPNAYSSQSSGKTNPVRKSANYHPNIWGDLFLQSSPQDYMLDEEIKIRA

EGLKDQVRRMLQNASDPLVEMNLIDSIQRLGVAYHFETEIEEKLKRWYNAWADGLNDGED

LHAVALQFRLLRQHGYNVSPDVFCKFKDNTGNFKENLARDIRSLLSLYEASFLGTHEEDI

LDEANKFSRDHLQLAVHHLSSPLSTLVKLSLELPLQKRVQRLQARCYISIYEEERYDALL

EFAKLDFNMLQSLHKRELRNISLWWKKIDFSRKLPFIRDRLVECYFWILEVYYQPQYSRG

RMMATKIIYLTSVMDDIFDVYATPEELGPFTDAILRWDRGAADKLPDYMKVHFLEVLDCV

DEFEKELAEEGQSYRIYYLKEAFKEVSKAYNKEAQWFHSGYSPSYSEYMRLALVTSGYPL

VSVVSMVGMGEIVTKEALEWAMSTPQLIKACSAIARIKDDIQSNESEQERGHVASSVQLY

MKENECTYDEACVMLQGKVERAWKDINKECLKPTPVPMPFLVRVVNLARVIEVLYQNRDG

YTDSTHETKERILSV

>CcTPS9

MSVVLTSSLSEAPNKHHLEGNKTSEEVRKSVNYVPEIWGDRFVASSPENLKPDAQTQQRA

NELKEEVRRMLRNVDDHLQELNLIDAVQRLGVAYHFEEEIAQALLRMYNSGRDYGDDLHA

VALQFRLLRQEGYNVSPDVFMKFKDEEGRFKRTLAGDTRSLLSLYEAAYMGIHGENILDE

AIAFTREHLKLALPCLNPPFSTMVDLALELPLRKRLERLQTSYYISIYQEDKDRSNILLE

FAKRDFNLLQLLHKQELREVSMWWKSWDFGAKLPFIRDRIVECYFWILGVYFEPQHSRAR

KMMTKIISLTSIMDDIYDVHGTLEELEPYTDAIQRWDRSIIDQFPDYLKLHFSALLDTVE

KFEEELALEGKSYRIPYLKQAFKGLSKGYLTEVQWSNSGHVPTLEEYMTNAVMSSGYPML

SVASYVGMGDVATKEAFDWGVSVPKLIKVAAAISRLENDITSYQLEQERDHVATSIQIYM

NENGGTYEEACERFRRMAADAWKDVNKECLKPPPAPMPILMRILNLTRASEMIYQHRDGY

TNPTYETKEGVLSVLVNPIPV

>CcTPS11

MEECIQGLLQKIKEELFSTVDICTYVSASAYDTAWLAMIPHAEDPSRPMFPQCLEWILHN

QKQVGFWGDELTIDFIPSTLACMVALKTWDVGLDNIHKGLDFIHGHMEKLLLEVQGNTPR

WFAVVFPGMLEHAWSKNLELFPQGLPHVLKSTFIERQRILETKEVTIHAYRPPLIYYYEA

LPSTYKMESFMEVVRHLSADGSLFQSPSATASAFMDTKDKRCQDYLESMVQRCGFSVPPT

YPIDEELIKLCAVDRLERLGLDEHFQDEIDKVLHDVYRNWTKRTAAPIGVYISPLQFYKD

CMAYRLLAMHGYSVSPSSFPTLLDHENLVSHVKHCHDNYFLSNLLNVYRAWELLFAGDGH

QQNIKARTRILLEKGISHENTSVATNLKKEIEYELNLPWLARMDHLEHIECIERGEASDM

WTGKAWFRISCLNSKTLLTLAKENIAHRQSIYRSELKELQGWARDNGISSIGFGRERTVY

CYFAMASTAYHPCLRAVRMTSAKNGILLTIIDDFFDIKGSMDELTCFTDAVERWDGKGIH

GVGKGLFNALNDLINDTATQAFPIQGRDVTEHLRDMWCQVIRSWLQEAEWSKKDYTPSID

EYLRVAGISTAVQTIVLPSLYFVGPIISEEMIHHPDYIKIAELVMISARLLNDLETYQKE

SKEGKPNLVLLFMQGRPERKIAHAKADIAEILDRTKKELLEHVLINDKNCVPKSCRKIHL

IALKIFQMFYYSTNRYDSPTAMIQDINKAIYEPL

>CcTPS12

MEKGIQGLLQKIKEELFSSVDVCTHVSTSAYDTAWMAMIPHAEDPSRPMFPQCLEWMLNN

QKELGFWGDELTIDCIPSTLACMIALKTWDMGHYNIEKGLDFIRGHMEKLLLELQDNYPR

WFAVVFPGMLERAWSKGLELFPQGSPQVVKSTFIERQRILETKEVPIHGYGPPLIYYYEA

LPPTYIDCAEVVRHLNADGSLFQSPSATASAFMDTKDKRCQHYLESMVQRRGCSGVPPVH

PIDDELVKLCVVDRLERLGLDEHFHEEIGMVLHDVYRNRIECNAEPIEVYISPLQIYKDC

MAYRLLTMHGYRVSQSTFSTLLDHENLESHLKKNHGYFLSCLLNIYRASELLFLGKREPQ

NLQSHSRILLEKGMSDEKINLATNLRKEIEHELSIPWLARVDHLDHRECIERGEASDIWT

GKAWFKISCLNNNILLVLAKENYALRQSIYRSELEELKGWTRDNGISSIGFGREKTVYCY

FAMATTAYRPHLRAVRMTSAKNAILFTIIDDFFDVQGSMDELTRFADAVERWEGEGIHGV

GKVLFNALNDLINDTAMQAFPIQGRDITGHLRDIWCQVIRSWVQEAEWGRKDYTPSIDEY

LKVATVSVASQTAVIPSLYLVGPSISEEMIRHPDYKKIVELLMISTRLLNDLQSYQKERK

EGKNNLVLLFMERHPDSKIEHAYTYITETLDRTKKDLLEHVLIDDKNCVPKSCRKIHLII

LKIFQMFYNSSNRYDSPTAMIQDINKAFYEPLCMESDCDNKL

>CcTPS13

MASPTAFLLLRPSLSPIHHASFANPAIAYRRPAPEKLSDFECLRSGWKRADLAPSGSRCS

TISKLHVMRDVFRDVVSVAGSSETLWRVDDDENKALVQVSVPDEISRRVNGIRAMLRSMD

DGEISVSAYDTAWVALVEDINGNGSPQFPSSLQWIVDNQLSDGSWGDYKIFSAHDRVLNT

LACVVALKSWNIYPERCKRGISFLGENMGRLEEEDAEHMPIGFEIAFPSLIEIARNLGLE

VNFDSPVLKEIHAKRNLKLSRIPKEMMHQVPTTLLHSLEGMPDLDWEKLLKLQSPDGSFL

FSPSSTAFALKQTKDEKCLKYLKKAVDKFKGGVPNVYPVDLFEHLWAVDRLERLGISRYF

EPEIKDCLDYVYRYWTEDGICWARNSKVRDVDDTSMAFRLLRLHGYDVSADAFRHFEKGG

EFFCFAGQSGQAVTGIYNLNRASQVSFPGEKILEDARTFSYRFLREKQASKQLLDKWIIT

KDLPGEVGYALDFPWYASLPRIETRFYLEQYGGEDDVWIGKTLYRMPYVNNNEYLELAKL

EFNNCQALHQSEWLDMQRWYEEVKLREYGVSRRAVLRAYFVAASSIFEPERANERLAWTR

TAVLMEAFASYLSNNATTVQDKRAFFEDFIRSSSKRTDKGLVGACLDTLRLLSLETFVAH

RSNVHFHLERAWEDWLMTWRDREHSAPQGATELLVRTINLCAGRSVSKELLSHPHYVRLA

TRTHTLCQRLSRLPRLIKKAEQTTDGNGTNPSSGVDSQQPMETEMQELVQYVLQTSDGID

LATKQTFLTVAKSFYYTAHCSPTNFNLHMEKVLFERVV

>CcTPS14

MKTIMDLVSEFKEEMFSSAIDLCSLVSPSAYETAWVAMIPDPDRPDQPLFRQCLEWIIEG

QREEGFWGERGTLECLCSSLACMAALKTWDVGPCNIDRGLAFVHAKTEKLLGEEGGDFPR

WFYIVFPAMVELAQTKGLNVFPDGMKDVVKSIYRQRQGILEKEASLGDAYHPPLLSYLEA

LPTPRGVIHHKTILQNQSKDGSLFQSPSATARAFMITGNKGFRLYLESMVQRCRHGGVPH

LYPIDEELIKLSMVDQLGRLGLSEHFSEEIEDVLRRVYKNWMGQEAEEEDKRESNRLVPL

RLYKDSLAFRLLRMHGYRVSPRRLCWFLQHEDIASYMEQNYDSLLSTLLNVYRATEVIFP

GENELEEARSFSRTLLERGLNIKNTKDSVVTFNIQREIEHELRLPWLARSEHLEHRVGIE

GSESDDYLWMGKASYYRISCLNSNNLLRLAKENYTLRQFIFKNELEQVTRWGRDSGLRDI

GFGREKTTYCYYSVASGAYLPSLSDVRMLVCKSAILVTVADDFFDMEGSMDELNTLTEAV

RRWDGKELSGHGKVIFDALSDLVDDTIKMFYDRHQVDVTKSVRDLWCETFMSWLKEADWS

RRRYAPSIDEYIQTGTTSIAAHTMILPACYLVSPGLSMHQMTHANVDVITKLLMVSARLL

NDMQSYEKEKEDGKLNLVLLYLKENLELDIENATDCIGKILDKQKKEFLKMTLEDGITGA

PKQWRQLHLCGLKAFQMFYNSNNGFDSPTALINHINKAFYEPLVTDTWKTTPQQTLQPHP

GSKNCNSLVRAHIGKSFQDHGRRRLLYYSNIETSGMRVLKDRVFPLRFPIVRHSHKFYTR

VLMPTSVTKPLLWR

>Tw_kaurene_syn1

MMSLSHPNWTRHSSLPLSAALPKSKSEVLTETNATILYFQETKERIKKMFDKTQLSVSAYDTAWVAMVSS

PNSRQAPCFPECVNWLLDNQLSDGSWGLPPHHPSLVKDALSSTLACLLALKRWGLGEQQMTKGLQFIESN

FTSINNEEQHTPIGFNIIFPGMIETAIDMNLNLPLRSEDINVMLHNRDLELRRNKLEGREAYLAYVSEGM

GKLQDWEMVMKYQRKNGSLFNSPSTTAAALSHLGNAGCFHYINSLVAKFGNAVPTVYPSDKYALLCMIES

LERLGIDRHFSKEIRDVLEETYRCWLQGDEEIFSDADTCAMAFRILRVHGYEVSSDPLAQYAEDHFSHSF

GGHLKDFSTALELFKASQFVIFPEESGLEKQMSWTNQFLKQEFSNGTTRADRFSKHFSIEVHDTLKFPFH

ANVERLAHRRNIEHHHVDNTRIFKTSYCFSNISNADFLQLAVEDFNSCQSIHREELKHLERWVVESKLDR

LKFARQKMAYCYFSAAGTSFSPELSDARISWAKNTVLTTVVDDFFDIGGSEEELANLVHLLEKWDANGSP

HYCSEQVEIIFSALRNTICEIRDKALAWQGRSVTHHVIEIWLDLLKSMLREAEWARNKVVPTLDEYVENG

YVSMALGPTVLSTVYLIGPKVSEEVVRSPEFHNLFKLMSTCGRLINDTRTFKREYEDGKLNSVLLHMIHS

GSGATEEEAIEKIRGIIADGRRELLGLVLQEKDSVVPRACKDLFWKMVQVLHLFYMDGDGYTSADMMLNA

VNALIREPISL

>Tw_copalyl_diphosphate_syn2

MDAFGDVVPETLENSIEAEAVKIKEQVDNIKSFLGSIEDGDMSSSAYDIAWVAMIEDVNQKGVPQFPSCL

LWIVENQLDDGSWGYSGLFSAYDRTLNTLACVIALKSWNIHPEKCEKGLSFLRENISLLEKEKAERMLGG

FELVFPPLIEMARRLDIEVPDCSSTLPDICAKRNLKLTRIPKDRMHSVPTTILYSLEGIQDLDWEKILKL

QCHDGSFLTSPSSTAFAYMKTKDENCFTYLKQVTKRFNGAVPSMHPIDLYERLWAVDRLRRLGISRFFEG

EIKEYMSFVQRYWTKDGMSWTRNSQLRDTDCTAMGFRLLRLHGYQVFPDVFQNSKKGNEFICYEGQWNEA

ISVMSNLYRASQLMFPNEKILEEAKQFTSKFLREKQASNQLIDKWILTTDLSGEVAFALDVPWYACFPRL

EARFYIEQYGAEDVVWIAKTLYSVPYIDNNMYLELAKLDYNNCQALHRVEWISIQMWYEGFEFGGSGVNR

RSLLCAYFVAAASIFEQERSMERLAWVKTAILIETVASYFINSTKQRRKSFVNEFRKCVRNERMMETNTP

TTEKLIQAILETLNQFCQDIMVTEDIVTRHLHGLWEKWLLTWQEEGNRLTCEAELLAQTINFMAGYKVFK

EFSPLYVQLINITNRICSQLGRHQERKNKNGQDSCNDNPHRITTPQIERDMQELVQLVLINNSSDGDGMD

VNMKQTFPTIAKIFYYIAFMDPETVNSHIAKVLFERVD

>Tw_copalyl_diphosphate_syn1

MYSSQTTHVFSSPLHCTIPKSSSFFLDAPVVRLHCLSGHGAKKKRLHFDIQQGRNAISKTHTPEDLYAKQ

EYSVPEIVKDDDKEEEVVKIKEHVDIIKSMLSSMEDGEISISAYDTAWVALIQDIHNNGAPQFPSSLLWI

AENQLPDGSWGDSRVFLAFDRIINTLACVVALKSWNVHPDKCERGISFLKENISMLEKDDSEHMLVGFEF

GFPVLLDMARRLGIDVPDDSPFLQAIYVQRDLKLKRIPKDILHNVPTTLLHSLEAIPDLDWTKLLKLQCQ

DGSLLFSPSSTAMAFINTKDENCLRYLNYVVQRFNGGAPTVYPYDLFEHNWAVDRLQRLGISRFFQPEIR

ECMSYVYRYWTKDGIFCTRNSRVHDVDDTAMGFRLLRLHGYEVHPDAFRQFKKGCEFICYEGQSHPTVTV

MYNLYRASQLMFPEEKILDEAKQFTEKFLGEKRSANKLLDKWIITKDLPGEVGFALDVPWYVSLPRVEAR

FFIQHYGGEDDVWLDKALYRMPYVNNNVYLELAKLDYNYCQALHGTEWGRIQKWYEECKPRDFGISRECL

LRAYFMAAASIFEPERSMERLAWAKTAILLEIIVSYFNEVGNSTEQRIAFTTEFSIRASPMGGYINGRKL

DKIGTTQELIQMLLATIDQFSQDAFAAYDHDITRHLHNSWKMWLLKWQEEGDRWLGEAELVIQTINLMAD

HKIAEKLFMGHTNYEQLFSLTNKVCYSLGHHELQNNRELEHDMQRLVQLVLTNSSDGIDSDIKKTFLAVA

KRFYYTAFVDPETVNVHIAKVLFERVD

>Tw_kaurene_syn2

MMSLSHPNCIRHSSLPISATLPKSKSEVLTETDATILYFQETKERIKKMFDKAELSVSAYDTAWVAMVSS

PNSHQAPCFPECVNWLLDNQLSDGSWGLPPHHPSLVKDALSSTLACLLALKRWGLGEQQMTKGLQFIESN

FTSINDEEQHTPIGFNIIFPGMIETAIDMNLNLPLRSEDINVMLHNRDLELRRNKFEGREAYLAYVSEGM

GKLQDWEMVMKYQRKNGSLFNSPSTTAAALSQLGNAGCFHYINSLIAKFGNAVPTVYPSDKYALLCMIES

LERLGIDSHFSQEIRDVLEETYRCWLQGDEEIFSDADTCAMAFRILRVHGYEVSSDPLTQYAEHHFSRSF

GGHLKDFSTALELFKASQFVIFPEESGLEKQMSWTNQFLKQEFSNGTTRADRFRKYFSIEVHDTLKFPFH

ANVERLAHRRNIEHHHVDNTRILKTSYCFSNISNADFLQLAVEDFNRCQSIHREELKHLERWVVETKLDR

LKFARQKMAYCYFSAAGTCFSPELSDARISWAKNSVLTTVADDFFDIVGSEEELANLVHLLENWDANGSP

HYCSEPVEIIFSALRSTICEIGDKALAWQGRSVTHHVIEMWLDLLKSALREAEWARNKVVPTFDEYVENG

YVSMALGPIVLPAVYLIGPKVSEEVVRSPEFHNLFKLMSICGRLINDTRTFKRESEAGKLNSVLLHMIHS

GSGTTEEEAVEKIRGLIADGQRELLRLVLQEKDSIVPRACKDLFWKMVQVLHLFYMDGDGFSSPDMMLNA

VNALIREPISL

>Am_(E)-b-ocimene_syn

MAFCISYLGAVLPFSLSPRTKFAIFHNTSKHAAYKTCRWNIPRDVGSTPPPSKLHQALCL

NAHSTSCMAELPMDYEGKIQGTRHLLHLKDENDPIESLIFVDATQRLGVNHHFQKEIEEI

LRKSYATMKSPSICKYHTLHDVSLFFCLMRQHGRYVSADVFNNFKGESGRFKEELKRDTR

GLVELYEAAQLSFEGERILDEAENFSRQILHGNLASMEDNLRRSVGNKLRYPFHKSIARF

TGINYDDDLGGMYEWGKTLRELALMDLQVERSVYQEELLQVSKWWNELGLYKKLTLARNR

PFEFYMWSMVILTDYINLSEQRVELTKSVAFIYLIDDIFDVYGTLDELIIFTEAVNKWDY

SATDTLPDNMKMCYMTLLDTINGTSQKIYEKYGHNPIDSLKTTWKSLCSAFLVEAKWSAS

GSLPSANEYLENEKVSSGVYVVLIHLFFLMGLGGTNRGSIELNDTRELMSSIAIIVRIWN

DLGCAKNEHQNGKDGSYLDCYKKEHINLTAAQVHEHALELVAIEWKRLNKESFNLNHDSV

SSFKQAALNFARMVPLMYSYDNNRRGPVLEEYVKFMLSD

>Am_myrcene_syn

MIYIWICFYLQTTLLPCSLSTRTKFAICHNTSKLHRAAYKTSRWNIPGDVGSTPPPSKLH

QALCLNEHSLSCMAELPMDYEGKIKETRHLLHLKGENDPIESLIFVDATLRLGVNHHFQK

EIEEILRKSYATMKSPIICEYHTLHEVSLFFRLMRQHGRYVSADVFNNFKGESGRFKEEL

KRDTRGLVELYEAAQLSFEGERILDEAENFSRQILHGNLAGMEDNLRRSVGNKLRYPFHT

SIARFTGRNYDDDLGGMYEWGKTLRELALMDLQVERSVYQEELLQVSKWWNELGLYKKLN

LARNRPFEFYTWSMVILADYINLSEQRVELTKSVAFIYLIDDIFDVYGTLDELIIFTEAV

NKWDYSATDTLPENMKMCCMTLLDTINGTSQKIYEKHGYNPIDSLKTTWKSLCSAFLVEA

KWSASGSLPSANEYLENEKVSSGVYVVLVHLFCLMGLGGTSRGSIELNDTQELMSSIAII

FRLWNDLGSAKNEHQNGKDGSYLNCYKKEHINLTAAQAHEHALELVAIEWKRLNKESFNL

NHDSVSSFKQAALNLARMVPLMYSYDHNQRGPVLEEYVKFMLSD

>Am_nerolidol/linalool_syn

MSNLHVKKTDRIASGFDVAYLSAANKTHQWSIADDIASTPGSPRLHYPMDFNGQSTRTPD

NFSMEYDKKIEEIKNLLRSKREEEPIDRLMFVDAIQRLGVNHNFEELIETILRNYYESTS

ANICGFHTLHDVSLFFRLMRQHGYDISSDVFNKFKGDDGRFRGELQRDTRGLMELYEASQ

LRFEGEYTLDEAESFSSQNLNKYLADMDCSSCRLVTNKLQHPYRKSIGRLTTRYDFRGKN

QWGKTLHELAAMDLRMRKSEYQKELFQVSEWWKELRIAENLSLARNQPLKWYTCSMAILI

DDITLSEQRIELTKSITFIYLIDDIFDVYGSPEELVIFAEAVSKWDYAAVETLPDYMKLC

YKSLLDTTNEIGYKIYEKYGYNPIDSLKTTWASLCNAFLEEAKWFASGNLPNATKYLENG

KVSSGVYVVMVHLFFLLGLGGTCGSAIHLNDTSKLMSSVATILRLWDDLGSAKDEHQDGK

DGSYIECYMKEHISLSTEQAQQHAIDLISSEWKLLNKECFNLNHVSTSSIKKAALNTARM

VPLMYSYDENQGLPILEEYVKIMLFD

>At_1.8-cineole_syn

MATLRISSALIYQNTLTHHFRLRRPHRFVCKSMTKTTPDTTLVELSRRSGNYQPSPWNHC

YLLSIENKYASETEVITRDVLKKKVKSMLDDEKKSRLEQLELIDDLQKLGVSYHFEIEIN

DTLTDLHLKMGRNCWKCDKEEDLHATSLEFRLLRQHGFDVSENIFDVIIDQIESNTFKTN

NINGIISLYEASYLSTKSDTKLHKVIRPFATEQIRKFVDDEDTKNIEVREKAYHALEMPY

HWRMRRLDTRWYIDAYEKKHDMNLVLIEFAKIDFNIVQAAHQEDLKYVSRWWKDTCLTNQ

LPFVRDRIVENYFWTVGLIYEPQFGYIRRIMTIVNALVTTIDDIYDIYGTLEELELFTSM

VENWDVNRLGELPEYMRLCFLILYNEINGIGCDILKYKKIDVIPYLKKSWADLCRTYLVE

AKWYKRGYKPSLEEYMQNAWISISAPTILIHFYCVFSDQISVQNLETLSQHRQHIVRCSA

TVLRLANDLGTSPTELARGDVLKSVQCYMHETGASEERARDHVHQMISDMWDDMNSETKT

ACNSSSRSRGFKEAAMNLARMSQCMYQYGDGHGCPEKAKTIDRVQSLLVDPIPLDVNRLG

>At_linalool_syn

MALIATKISSRSCFVSAYPNNSPTFLISKFPNTVDSLSPANTAKRSILRNVHASVSNPSK

QFHNKTSLEYSHELNIKKIKNILSANVDVPSENLEMIDVIQSLGTDLHFRQGIEQTLHMI

YKEGLQFNGDLHEIALRFRLLRQEGHYVQESIFKNILDKKGGFKDVVKNDVKGLTELFEA

SELRVEGEETLDGAREFTYSRLNELCSGRESHQKQEIMKSLAQPRHKTVRGLTSKRFTSM

IKIAGQEDPEWLQSLLRVAEIDSIRLKSLTQGEMSQTFKWWTELGLEKDVEKARSQPLKW

HTWSMKILQDPTLTEQRLDLTKPISLVYVIDDIFDVYGELEELTIFTRVVERWDHKGLKT

LPKYMRVCFEALDMITTEISMKIYKSHGWNPTYALRQSWASLCKAFLVEAKWFNSGYLPT

TEEYMKNGVVSSGVHLVMLHAYILLGEELTKEKVELIESNPGIVSSAATILRLWDDLGSA

KDENQDGTDGSYVECYLNEYKGSTVDEARTHVAQKISRAWKRLNRECLNPCPFSRSFSKA

CLNIARTVPLMYSYDDDQRLPDEYLKSLM

>At_myrcene_syn

MATLLQIGSGVIYSNALRKTLRRPQSSTCIIVTETTPCNKSPTVQRRSANYQPSRWDHHH

LLSVENKFAKDKRVRERDLLKEKVRKMLNDEQKTYLDQLEFIDDLQKLGVSYHFEAEIDN

ILTSSYKKDRTNIQESDLHATALEFRLFRQHGFNVSEDVFDVFMENCGKFDRDDIYGLIS

LYEASYLSTKLDKNLQIFIRPFATQQLRDFVDTHSNEDFGSCDMVEIVVQALDMPYYWQM

RRLSTRWYIDVYGKRQNYKNLVVVEFAKIDFNIVQAIHQEELKNVSSWWMETGLGKQLYF

ARDRIVENYFWTIGQIQEPQYGYVRQTMTKINALLTTIDDIYDIYGTLEELQLFTVAFEN

WDINRLDELPEYMRLCFLVIYNEVNSIACEILRTKNINVIPFLKKSWTDVSKAYLVEAKW

YKSGHKPNLEEYMQNARISISSPTIFVHFYCVFSDQLSIQVLETLSQHQQNVVRCSSSVF

RLANDLVTSPDELARGDVCKSIQCYMSETGASEDKARSHVRQMINDLWDEMNYEKMAHSS

SILHHDFMETVINLARMSQCMYQYGDGHGSPEKAKIVDRVMSLLFNPIPLD

>Cb_linalool_syn

MLSIQSHVDEIKRGSLWNLRLSPSAYDTAWLALIPDPDDPTRPMFAKCMHWLVQNQSMEG

FWAADDDIDTEPVALDCLPATLACLIALKRWGAAPNNINKGLGFFERNVEELLLRKGKLS

DVPRWFTVTFLAMLELAIASGLKVAFPDNLIKVLDELFENRNTILLREELSDKTQYAPLL

MFLEALPPSYVKLDDLNQYLERNLGNDGSLYQSPSATARAYMATGNTKCLSYLKSLTNTY

LDGGVPSLYCMDEELQQLVMVNQLVRPGLTEYFVPEIEQILLQVEQNYKCKRSPPPRNAL

HNVVAELYKDSLAFWLLRINGHSVSPSMFCWFLHNNEIRHHIEANYMYFDNVLLNVYRAT

NLMFLGEAEAEEARSFSIKYLNKITQQKVQTPITTNIHISSSLQRMIEYELKLPWTARMD

HLEHLMWIEEAASDALWMGKSSHHRLSRLHNLDLQQLKLKNYTLRQSVYRNEHEEVKRWS

KERGLCDMGFGREKTTYCYYARAASTSLPCSSSVRHLLAKAAIVVTVADDFFDEKGSMDD

LENLTDAVRRWEVEGLSRHSRIIFEALDDVVNEIRLKCFQKHGKDIKDNLHHLWYETFNS

WLMEAKWGKGNIKPSLDVYLQNAMISVAVHTMLLPVSCLLSPVFPVHQWSARHHQDDDDM

TSLLLFTVRLLNDTQSYLKEEEGKINYVWLYMNEKEKVKLEDSIQHVQSLINLKKQQFVQ

HVLTNSHLPKPYKQLHLSCLKIFNMFFNSSNLYDSHDDTHLFHDIQKAFIIPPQVHKFKP

RYAKNPQQEATTSAAATSSAAPTTSDQYASQGL

>Cc_linalool_syn

MQPITKSSSTSSELEFLVDKVKRESLSSSSSDTQNLFLSASPYDTAWLALIPHPHHHHHH

GPPMFEKCLQWILHNQTPQGFWTAAAGDNISDTDDDVTLDCLLSTLACLVALKRWQLAPD

MIHKGLEFVHRNTERLVMKQKPSDVPRWFTIMFPAMLELAGASGLRVDFSENLNRILVEL

TQNRDDILTREEVGEKKQYSPLLLFLEALPAQSYDSDVLKQIIDKKLSSDGSLFQSPSAT

ARAYMITGNTRCLSYLHSLTNSCSNGGVPSFYPVDDDLHDLVMVNQLTRSGLTEHLIPEI

DHLLLKVQKNYKYKKASPKSLYGIAAELYKDSLAFWLLRVNSHWVTPSIFCWFLNDEEIR

DHIETNYKEFAAVLLNVYRATDLMFSGEVQLVEARSFATKYLEKILATGNIQKTNADISS

SLHKMIEHELRVPWTARMDHVENRIWIEEIASGALWFGKSSYLRLSCLHKINLQQLAVKN

YTLRQSVYRDELAEVERWSKERGLCDMGFGREKTGYCYYAFAASTCLPWSSDVRLVLTKA

AVVITVADDFFDVEGSMDDLEKLTDAVRRWDAEGLGSHSKTIFEALDDLVNEVRLKCFQQ

NGQDIKNNLQQLWYETFHSWLMEAKWGKGLTSKPSVDVYLGNAMTSIAAHTMVLTASCLL

GPGFPVHQLWSERHHQDITSLLMVLTRLLNDIQSYLKEEDEGKINYVWMYMIENNQVSID

DSVRHVQTIINVKKQEFIQRVLSDKHCNLPKSFKQLHFSCLKVFNMFFNSSNIFDTDTDL

LLDIHKAFVSPPQVPKFKPHIKPPHQLPATLQPPHQPRQIMVNKKKVEMVYKSYHHPFKV

FNLPKKQSSGHGTMNPRASILAGPNIKLCFS

>Cl_(-)-beta-pinene_syn

MALNLLSSIPAACNFTRLSLPLSSKVNGFVPPITRVQYHVAASTTPIKPVDQTIIRRSAD

YGPTIWSFDYIQSLDSKYKGESYARQLEKLKEQVSAMLQQDNKVVDLDTLHQLELIDNLH

RLGVSYHFEDEIKRTLDRIHNKNTNKSLYATALKFRILRQYGYNTPVKETFSRFMDEKGS

FKSSSHSDDCKGMLALYEAAYLLVEEESSIFRDAKSFTTAYLKEWVIEHDNNKHDDEHLC

TLVNHALELPLHWRMPRLEARWFIDVYENGPHMNPILLELAKVDFNIVQAVHQENLKYAS

RWWKKTGLGENLNFVRDRIVENFMWTVGEKFEPQFGYFRRMSTMVNALITAVDDVYDVYG

TLEELEIFTDAVERWDATAVEQLPHYMKLCFHALRNSINEMTFDALRDQGVDIVISYLTK

AWADICKAYLVEAKWYNSGYIPPLQEYMENAWISIGATVILVHANTFTANPITKEGLEFV

KDYPNIIRWSSMILRFADDLGTSSDELKRGDVHKSIQCYMHEAGVSEGEAREHINDLIAQ

TWMKMNRDRFGNPHFVSDVFVGIAMNLARMSQCMYQFGDGHGCGAQEITKARVLSLFFDP

IA

>Cl_terpinene_syn

MALNLLSSLPAACNFTRLSLPLSSKVNGFVPPITQVQYPMAASTSSIKPVDQTIIRRSAD

YGPTIWSFDYIQSLDSKYKGESYARQLEKLKEQVSAMLQQDNKVVDLDPLHQLELIDNLH

RLGVSYHFEDEIKRTLDRIHNKNTNKSLYARALKFRILRQYGYKTPVKETFSRFMDEKGS

FKLSSHSDECKGMLALYEAAYLLVEEESSIFRDAIRFTTAYLKEWVAKHDIDKNDNEYLC

TLVKHALELPLHWRMRRLEARWFIDVYESGPDMNPILLELAKVDYNIVQAVHQEDLKYVS

RWWKKTGLGEKLNFARDRVVENFFWTVGDIFEPQFGYCRRMSAMVNCLLTSIDDVYDVYG

TLDELELFTDAVERWDATTTEQLPYYMKLCFHALYNSVNEMGFIALRDQEVGMIIPYLKK

AWADQCKSYLVEAKWYNSGYIPTLQEYMENAWISVTAPVMLLHAYAFTANPITKEALEFL

QDSPDIIRISSMIVRLEDDLGTSSDELKRGDVPKSIQCYMHETGVSEDEAREHIRDLIAE

TWMKMNSARFGNPPYLPDVFIGIAMNLVRMSQCMYLYGDGHGVQENTKDRVLSLFIDPIP

>Cm_copalyl_diphosphate_syn

MKALSLSRPFPCSSDATKLSSRPPPPPPVGSCSFKVESIRSSRIIKCNAISKPPTQEYSD

VLQSGVPVIKWQQFVEDDIESETTAHVLISKEIEERVNRIKSMLSSMDDGDISISAYDTA

WVALIPRVLDGVKTPLFPSSLEWIAQNQLPDGSWGDSGIFSAHDRILSTLACVLALNSWK

LHPDKSEKGMVFLNKNISKLEDENAEHMLIGFEVAFPSLMEFAKRLNLQVPTDSPVLQEI

NHRRSIKLTRIPKEIMHKVPTTLLHSLEGMEGMEGLDWGMLLKLQAPDGSFLKSPASTAF

AFMKTNNSNCFKYLESVVSRFNGGVPNVYPVDLFEHIWAVDRLQRLGVSRFFHPEIVESV

DYLRRHWTDKGICWARDVEFYDIDDTAMGFKLLRLFGHEVSAEVFKNFEKDGEFVCIAGQ

STQAVTGMFNLYRASDQVMFPGEKILEDAKQFSYKFLREKQAADELLDKWIITKDLPGEV

GYALDVPWFASLPRVETRYFIEQYGGENDIWIGKTLYRMFKVNNDTYLELAKLDYNKCQL

LHQNEWVDIQKWYTENNLRDYGMRRTSLLFSYFGAACSIFEPERAKERLAWTKTAALVGA

IESHFKDANADQRRAFIQQFINFDAIDQAYDTNAWRAGNVQQKGGGQGLVGILLRTLTSI

SLDILVSHGFDITHHLHQAWEKWLFKWQEDGDVHKEEAELLVGTIILNSGCSTLEDLLSN

PQYQKLSYLTNKVCHQLGHFKKHKVTNGGIYKEKTENKMPPEIEEDMRKLLQMVIQNSSD

GNDIDSPIKNTFLTVAKSSYYAAYFDPWTINYHIAKVLFERVF

>Cm_ent-kaurene_syn

MYLSRPTGVARFAASSSSSSSASLFPGVDVDTTTKTGALHFEETKERIKKLFDKVELSVS

AYDTAWVAMVPSPNSLNQPLFPECINWVLDSQHADGSWGLLHNDQLLMKANLLSTLACVL

TLKRWNIGHDHMSKALDFIKSNIASATDENQRSPVGFDIIFPGMIEYAKDLNLNLPLAPT

NVDALVRKKELELRSCRSNSEGGKAYLAYVSEGIGKLQDWDMVMQYQRKNGSLFNSPSTT

AAAFMHRNDDGCFDYLRSLLQKFDGSVPTIYPLDIYARLHMVDSLQKFGIARHFKEEIRS

VLDETYRCWMQGEENIFLDASTCAMAFRMLRVEGYDVSSDQLTQFSEDIFPNCLGGYLKD

FGASLELYKASQIITHPDESVLENINSWTSRFLKHGLSSDSVWSDRTDSVVKQEAVNALE

FPYNATLERLISKRAMESYSGDIVRISKSPYACLNFGHQDFLELAVEDFNTLQRIHLKEL

EELQRWVVENKLDELKFFRLHLGYCYFAAAATLTDPELHDARIAWAQNGVLTTVVDDFYD

GGGSEEELDNLIELVEKWDPDGEVGYCSKDVEIVFLALHSTVCEIGRRALVWQGRSVMRN

VIDGWLALLKVMRKEAEWSTNKVVPSMGEYMEQAHVSFALGPIILPMLFFVGPKLSEEMI

GSCEYQKLYKLMSTAGRLKNDIRSYDRECKEGKLNILSLWMIDGGGNVTKEEAIEAIKGD

FERAIRELLGLVLQENTTIPRACKDLFWKLMSIVNLFYMEDDGYTSNRLMNTVKAMFEQP

MDLDALLNK

>Cm_S-(+)-linalool_syn

MLSSAASSSLAPLNSDPFIPMKNHNHETPNAPFDPSNDQNHETPNSLFNPIPQLVTDHFQ

SITHHQFCTSSTIILDGLIIKQREKVNQVREIVQNLKDPAEKMVMIDNLQRLGIDYHFRE

EIESILCSLSENDDAVSSIHDVALRFRLLREHGYHASLDVFNSFKDKEGRFKLQLTADIK

GLMSLYESSKLSTEGEDILDEVNDFASKNLIASMEFIEPDLEREVRHVLEHPFHMSLPRF

NIKKHLKDLQGKDGKTDAPIQELAILDFNILQSMHQSELKEVAKWWGDLGLSQELRFARD

QPLKWYMWPLAVLPNPKFSRYRIELTKPIALVYIIDDIYDVYGTLDELVVFTEAVNRWDP

SDINQLPRNMKLCFMALHNITNEIAYMVLKEHGWNPINSLKKTWRDLCNAFLVEAKWFAE

GDSPKADEYLRNGVTSSGVATVLVHLFFLVGNGITRESVDLVDSIPKLISRPAVILRLWD

DLGSAKDENQKGYDGSYVECYLKENATSSLESARRHVRHMISNAWKELNKECLSPYPFSP

TFIEASLNTTRMVQVMYSYDNDQRLPALEQHITSLLKESIPDKEIERPLASRT

>Cu_(E)-beta-ocimene_syn

MAHQLMTSVPALTRLQEPRSFISSLGSPSISKSNSNGFCASPIQCMSATKVRDKAINDNR

RSANYQPSMWSYDYLQSLSNGYVGESCAQRIEKLKGEVRLMLDNYKEVDDYVDALHCLEI

VDNLQRLGVSYHFEGEIKRFLNSIYNKRNSRRSSTYHAKENQESLLYAASLEFRLLRQHG

YDIHAHGTLSSFMDEKGKFKSCLGDDIKGILALYEAAYLLGEEESTIFHEAINFTTTHLE

EYVKKHNDDDGYFSALVNHALELPLHWRMVRLEARWFIDVYERGTDMNPVLVELAKLDFN

SVQAAHQDELKYVSWWWRKTGLGELHFARDRILENFFWALGEIWEPQFGYCRRMSTKVNA

LITTIDDVYDVYGTLDELEQFTNAVERWDVNAMDQLPYYMKLCFHVLHSSTNEMAFDTLK

DQGVHVVPYLKKAWADMCKSFLLEAKWYSSGYIPTLDEYIENAWVSVSGPVILLHAYTLI

ANPAKEEALQFLQEYPHIIRWPSMIFRLANDLATSSDEVKRGDVPKAIQCYMHETGASES

DARLHIRDLITAAWMKMNNKREGDENPDHLLLPNNFVQFAMNLARMAQCTYQNGDRHTVQ

DNSKNRVLPLLIHPIKS

>La_bornyl_diphosphate_syn

MTAARSFMAINSNMTTDHLHKFGKKNLEFGQGYSCNNASPMRLRPCCSLKLSTNAESQLD

STRRSGMYKPTLWDFDRIQSLNSVYTEEKYSTRACDLIQQVKKLLEESDWFRQLQLIDDL

QRLGLSYRFDDEINLILNTIYFEKKFCEKEMDLYSTSLAFRLLRQHGLKVSQEVFDCFKN

EEGDFEARLGDETNGILEMCEASFLATEGEETLELARLFTTNILQKKLDDERNELLIMDD

YLRTLIRHSLDLPLYWRVQRPSARWFIEAYATRSDMNPIMLELAKLDFNIVQATHQEELK

QVSRWWKESRLAEKLPFARDRVVENYLWNRGMLFPPQYGYPRIMNAKLFVLITVLDDIYD

VYGTLEETQLFTNLITRWDVEAIGQLPEYMRICYMAIDNNINELAYEVLKQHGLLIIQDL

RKFWADLCVAYGKEAEWYYTGYKPTLEEYLEVAWVSISAHLILGYMFFLTSNPIEKEASQ

SLSNYHNIIRNSAMVLRLADDLGTSPYEMQRGDVPKAVECYMNENGASTEEAREYVKHLL

REVWKETNGERFKESPFTPSFMRICADLGRMAQFMYQHGDGHGIRNLQMEDRIQSLIFEP

IV

>Mg_terpineol_syn

MALKLLFQCSPCSPSSLAPLQPVLVLVRPPSGAKARRNLRCCASTQVTELMTARRSANYH

PNIWDYDSVQSLTSDYKAYTYLERVEKLKEDVRRTLQEAVGLLDQLELVDCIHRLGVGYH

FDKEIKEILKTISTEPNNMGLIDGDLYAMALYFRLLRQHGYEVPQGVFNRFMDDSSSFKA

SLCNDVKGMLSLYEASYLALEGETTLDEAKAFTYRHLRGLKGNIDSNLKGLVEHALELPL

HWRVLRLEARWYIDTYERMEDMNPLLLELAKLDFNIVQNVYQGQVRKMSGWWKDLGLGQK

LGFARDRLMEGFLWTIGVKFEPQFAQCREVLTKINQLITTIDDVYDVYGSLEELELFTKA

VDRWDTNAMEELPEYMKICFLALYNTVNEIAYDTLKEQGVDVIPYLQKSWADLCKAYLVE

ARWYYSGYTPTLDEYLNNAWISIAGPVILVHAYVSMIQMITKEALLDCVGSYESIMQWSS

MILRLADDLATSTDELERGDVPKSIQCYMHENTASEVVAREQMRARISDIWKKMNKDVAL

SPLPQPFKAAAVNLARMAQCMYQHGDGHGNPHRESKDHILSLVVEPIQLMES

>Mp_(E)-B-farnesene_syn

MATNGVVISCLREVRPPMTKHAPSMWTDTFSNFSLDDKEQQKCSETIEALKQEARGMLMA

ATTPLQQMTLIDTLERLGLSFHFETEIEYKIELINAAEDDGFDLFATALRFRLLRQHQRH

VSCDVFDKFIDKDGKFEESLSNNVEGLLSLYEAAHVGFREERILQEAVNFTRHHLEGAEL

DQSPLLIREKVKRALEHPLHRDFPIVYARLFISIYEKDDSRDELLLKLSKVNFKFMQNLY

KEELSQLSRWWNTWNLKSKLPYARDRVVEAYVWGVGYHYEPQYSYVRMGLAKGVLICGIM

DDTYDNYATLNEAQLFTQVLDKWDRDEAERLPEYMKIVYRFILSIYENYERDAAKLGKSF

AAPYFKETVKQLARAFNEEQKWVMERQLPSFQDYVKNSEKTSCIYTMFASIIPGLKSVTQ

ETIDWIKSEPTLATSTAMIGRYWNDTSSQLRESKGGEMLTALDFHMKEYGLTKEEAASKF

EGLVEETWKDINKEFIATTNYNVGREIAITFLNYARICEASYSKTDGDAYSDPNVAKANV

VALFVDAIVF

>Ms_4S-limonene_syn

MALKVLSVATQMAIPSNLTTCLQPSHFKSSPKLLSSTNSSSRSRLRVYCSSSQLTTERRS

GNYNPSRWDVNFIQSLLSDYKEDKHVIRASELVTLVKMELEKETDQIRQLELIDDLQRMG

LSDHFQNEFKEILSSIYLDHHYYKNPFPKEERDLYSTSLAFRLLREHGFQVAQEVFDSFK

NEEGEFKESLSDDTRGLLQLYEASFLLTEGETTLESAREFATKFLEEKVNEGGVDGDLLT

RIAYSLDIPLHWRIKRPNAPVWIEWYRKRPDMNPVVLELAILDLNIVQAQFQEELKESFR

WWRNTGFVEKLPFARDRLVECYFWNTGIIEPRQHASARIMMGKVNALITVIDDIYDVYGT

LEELEQFTDLIRRWDINSIDQLPDYMQLCFLALNNFVDDTSYDVMKEKGVNVIPYLRQSW

VDLADKYMVEARWFYGGHKPSLEEYLENSWQSISGPCMLTHIFFRVTDSFTKETVDSLYK

YHDLVRWSSFVLRLADDLGTSVEEVSRGDVPKSLQCYMSDYNASEAEARKHVKWLIAEVW

KKMNAERVSKDSPFGKDFIGCAVDLGRMAQLMYHNGDGHGTQHPIIHQQMTRTLFEPFA

>Na_5-epi-aristolochene_syn

MASAAVGNYEEEIVRPVADFSPSLWGDHFLSFSIDNHVAQKYAQEIEPLKEQTRSMLVAT

GRKLVDTLNLIDTIERLGISYHFEKEIDEILDQIYNQNSNSSDLFTSALLFRLLRQHGFN

ISPEIFSKFQDENGKFKESLASDVVGLLNLYEASHVRTHADDILEAALAFSTIHLESAAP

HLKSPLREQVAHALEQCLHKGVPRVETRFFISSIYEKEQSKNNVLLRFAILDFNLLQMLH

KQELAEVSRWWKDLDFVTTLPYARDRVVECYFWALGVYFEPQYSQARVMLVKTISMISIV

DDTFDAYGTVKELETYTDAIQRWDINEIDRLPDYMKISYKAILDLYKDYEKELSSAGRSH

IVCHAIERMKEVVRNYNVESTWFIEGYMPPVSEYLSNALATTTYYYLATTSYLGMKSATE

QDFEWLSKNPKILEASVIICRVIDDTATYEVEKSRGQIATGIECCMRDYGVSTKEAMDKF

QQMAETAWKDLNEGLLRPTPVSAELLTPILNLARIVEVTYIHNLDGYTHPEKVLKPHIIG

LLVDSIDI

>Ob_selinene_syn

MSANCVSAAPTSPKNSDVEEIRKSATYHSSVWGNHFLSYTSDVTEITAAEKEQLEKLKEK

VKNLLAQTPDESTGKMELIDAIQRLGVGYHFTTEIQESLRQIHEGQIRNDDDDVRVVALR

FRLLRQGGYRAPCDVFEKFMDDGGNFKESLKKDVEGMLSLYEASYYGIDGEEIMDKALEF

SSSHLESMLHNISTKTNKSLLRRLQEALDTPISKAAIRLGATKFISTYREDESHNEDILN

FAKLDFNILQKMHQEEANYLTRWWEDLDLASKLDFARDRMVESYFWSLGVYFQPQYRTSR

IYLTKIISIVAVIDDIYDVYGSFDDLRSFTDVIQSWKISNADELPPYMRICFEALLGIYE

DMGDRIGAPYAIDTMKELVDTYMQEAEWCYTEYVPTVDEYMKVALVTGGYLMVATTFLTG

INNITKKDFDWIRNRPRLLQVAEVLTRLMDDIAGHGTEKKTTAVSCYMKEYECSEMEASR

ELSKQVKKAWKDLNDEWMEPRSSSAEIIGCIVNMSRVLHIMYSTGDDGFSDSSTRTTQAV

KTLLVDHPMN

>Pa_(-)-limonene_syn

MSPVSVIPLAYKLCLPRSLMSSSREVKPLHITIPNLGMCRRGKSMAPASTSMILTAAVSD

DDRVQRRRGNYHSNLWDDDFIQSLSTPYGEPSYRERAERLKGEIKKMFRSMSKDDGELIT

PLNDLIQRLWMVDSVQRLGIDRHFKNEIKSALDYVYSYWNEKGIGCGRDSVVADLNSTAL

GFRTLRLHGYNVSSEVLKVFEDQNGQFACSPSKTEGEIRSALNLYRASLIAFPGEKVMDD

AEIFSSRYLKEAVQEIPDCSLSQEIAYALEYGWHTNMPRLEARNYMDVFGHPSSPWLKKN

KTQYMDGEKLLELAKLEFNIFHSLQQEELQYISRWWKDSGLPKLAFSRHRHVEYYTLGSC

IATDPKHRAFRLGFVKTCHLNTVLDDIYDTFGTMDEIELFTEAVRRWDPSETESLPDYMK

GVYMVLYEALTEMAQEAQKTQGRDTLNYARKAWEIYLDSYIQEAKWIASGYLPTFQEYFE

NGKISSAYRAAALTPILTLDVPLPEYILKGIDFPSRFNDLASSFLRLRGDTRCYKADRAR

GEEASCISCYMKDNPGSTEEDALNHINSMINEIIKELNWELLRPDSNIPMPARKHAFDIT

RALHHLYKYRDGFSVATKETKSLVSRMVLEPVPL

>Pa_(-)-pinene_syn

PRAAGKSCLHKSLSSSAHELKTICRTIPTLGMSRRGKSATPSMSMSLTTTVSDDGVQRRM

GDFHSNLWNDDFIQSLSTSYGEPSYRERAERLIGEVKKMFNSMSSEDGELISPHNDLIQR

VWMVDSVERLGIERHFKNEIKSALDYVYSYWSEKGIGCGRESVVADLNSTALGLRTLRLH

GYAVSADVLNLFKDQNGQFACSPSQTEEEIRSVLNLYRASLIAFPGEKVMEEAEIFSAKY

LEEALQKISVSSLSQEIRDVLEYGWHTYLPRMEARNHIDVFGQDTQNSKSCINTDKLLEL

AKLEFNIFHSLQKRELEYLVRWWKDSGSPQMTFGRHRHIEYYTLASCIAFEPQHSGFRLG

FAKTCHIITILDDMYDTFGTVDELELFTAAMKRWDPSAADCLPEYMKVMYMIVYDTVNEM

CQEAEKAQGRDTLDYARQAWEDYLDSYMQEAKWIATGYLPTFEEYYENGKVSSGHRVAAL

QPILTMDIPFPPHILKEVDFPSKLSDLACAILRLRGDTRCYKADRARGEEASSISCYMKD

NPGATEEDALDHINAMISDVIRGLNWELLKPNSSVPISSKKHVFDISRAFHYGYKYRDGY

SVANIETKSLVKRTVIDPVTL

>Pd_bornyl_diphosphate_syn

MAAFNIIMRFALPTNYKPKYSLCGFVPNKTTKVTCSSVHSNAKTSDEVIKPGDEVSRGRR

SGNYEPPIWNFNYVQSSSSQYTAGRRSGNYEANMWDFDYIQSSSSQFTEDRYLERASELV

VQVKKLLEEELTEPIQQLELIDDLQNMGVSYHFEDEIKQILKSMYDDRVKKYNSKDSKNV

RDLYSTALEFRLSRQHGFTISQEVFDCFKNNKGGFEASLAEDTRGLLQLYEASFMLMEGE

ETLEQAKEFATSFLLKKLEDDTKHGILVDENLSLSVFHALELPIHWRTQRHNARWFIDAY

EKRSNRNSVVLELAKVDFNIVQATYQQEIKHISRWWEQTRLAEKLPFARDRLVENFLWTV

GWLREPQYGYARIMCTKLFIFITYVDDIFDVYGTLEELQLFRDVIRRWDIEAMGQLPNYM

QMCFLAIDNFINEMAYDVLKEQEFVIIPHLRKMWADLCTSYCQEAEWYYNKYMPTMDEYI

NNACISISTPLILSNTYFVVTNPIEEEVVQNFYKNPDVVRYSAMILRLADDLGTSEFEAE

RGDVPKAIECYMNESGASREEAREHVKFMIWEAWKKINKELLSNASFPQFFLRNAADLGR

AGQFMYQHGDGFGVNPHHHKEDVSTLFFEPL

>Pd_linalool_syn

MATINFIMAHAAIVRKRPNKSICHFVRKRVTCSSTPLSGAETATGANRPDSTIKIGDEIS

AGRRSGNYEPPTWDFDYVQSLSNHYTEEKYSRRAKELVAQVKKLLQEELKEPIQQLELID

DLQRMGISYHFKDKIKEILNSIYDDNHHRQDCKNSEIEADLYSTALKFRLLRQHGFTSSE

EVFDCFKNDNGDFKASLADDTRGLLQLYEASFLLMEGEETLEKAKEFATKFLQKNLYDEG

KQGMLIDDNLLSLVHRALEIPIHHRTQRLNARWSINAYGNRSDKNPVVHELAKLDFNLLQ

ATFQQELKHVSRWWKQTGLAEKLPFARDRLVECYFWTIGWLYEPQYVFARSAATKFVALI

NVIDDIYDIYGTLEELHLFQDAIQRWDIEAIGQLPQYMQLCFLALDNFINEIGYHVLKEQ

GFLAIPYLRKSWQDLCTSYFQEAKWYYAGYVPTLEEYIGNARISISSFLALSHTYFLVAN

PIEKEVLESLYNDPDLSRYSSVIFRLEDDLGTSLDELKRGDVPKAIECYMNESGANRDEA

QKHIEFLIWDTWKKLNKEQVANSLFPQFLCRNAVDLCRTAQLMYQHGDGHRISTLLFEPI

L

>Pf_limonene_syn

MYTGAIMHMAIPIKPAHYLHNSGRSYASQLCGFSSTSTRAAIARLPLCLRFRCSLQASDQ

RRSGNYSPSFWNADYILSLNSHYKDKSHMKRAGELIVQVKMVMGKETDPVVQLELIDDLQ

KLALSHHVEKEIKEILFKISTYDHKIMVERDLYSTALAFRLLRQYGFKVPQEVFDCFKND

NGEFKRSLSSDTKGLLQLYEASFLLTEGEMTLELAREFATKSLQEKLNEKTIDDDDDADT

NLISCVRHSLDIPIHWRIQRPNASWWIDAYKRRSHMNPLVLELAKLDLNIFQAQFQQELK

QDLGWWKNTCLAEKLPFVRDRLVECYFWCTGIIQPLQHENARVTLAKVNALITTLDDIYD

VYGTLEELELFTEAIRRWDVSSIDHLPNYMQLCFLALNNFVDDTAYDVMKEKDINIIPYL

RKSWLDLAETYLVEAKWFYSGHKPNLEEYLNNAWISISGPVMLWHVFFRVTDSITRETVE

SLFKYHDLIRYSSTILRLADDLGTSLEEVSRGDVPKSIQCYMNDNNASEEEARRHIRWLI

AETWKKINEEVWSVDSPFCKDFIACAADMGRMAQFMYHNGDGHGIQNPQIHQQMTDILFE

QWL

>Pf_mercene_syn

MYTGVMNMAFPMKPANYLHNSGSSNSSKLCGVSSTSTRAATARLRLRLRCSMQLSDQRRS

GNYSPSFWNTDYILSLNCDYEDERRMRGAAGELVEQVKMLMEKETDPIVQLELIDVLQKL

ALSHHFEKEFEGILFNISTIYDDKNRERDLYSTTLAFRLLRQHGYQVPQELFECFKNDKG

EFKESLSNDTKGLLQLYEASFLLTEGETTLELAREFATKFLQEKEKLNIDDDDDTNLISC

VRHSLDMPIYWRIQRPNARWWIHAYNRRTHINPLVLELSKLDFNIIQAQYQQELKQDLRW

WRNTCIAEKLPFARDRLVESYFWSTGIIQPRQHENARIMMAKALALITTLDDVYDVYGTL

EELELFIEAIRRWEISSIDQLPNYMQLCFLTINNFVDDTAYDVMKEKDINIIPYLRKSWV

DLAEAYLVEAKWFYGGYKPNLEEYLNNGWISVSGPAILCHVFFGVTDSITMETVESLFKY

HDLIRCSSTLVRLADDLATSLDEVSRGDVPKSIQCYMNDNNASEEEARLHVRWLIAETWK

EMNVEMVSADSPFCKDFIACAADMGRMAQYMYHNGDGHGMQNSQIHQQMTDFLFQKLAVR

DRASTARN

>Pt_farnesene_syn

MSSLAVDDAERRVGDYHPNLWDDALIQSLSTPYGASPYRDVAEKLIGEIKEMFASISIED

GDDEICYFLQRLWMIDNVERLGISRHFENEIKAAMEDVYSRHWSDKGIACGRHSVVADLN

STALAFRTLRLHGYSVCSDVFKIFQDQKGEFACSADQTEGEIKGILNLLRASLIAFPGER

ILQEAEIFATTYLKEALPKIQGSRLSQEIEYVLEYGWLTDLPRLETRNYIEVLAEEITPY

FKKPCMAVEKLLKLAKIEFNLFHSLQQTELKHLSRWWKDSGFAQLTFTRHRHVEFYTLAS

CIAMEPKHSAFRLGFAKLCYLGIVLDDIYDTYGKMEELELFTAAIKRWDTSTTECLPEYM

KGVYMAFYDCVNEMARQAEKTQGWDTLDYARKTWEALIDAFMEEAKWISSGYVPTFQKYL

DNGKVSFGYRAATLQPILTLDIPLPLHILQEIDFPSSFNDLASSILRLRGDICGYQAERS

RGEQASSISCYMKDNPGSTEEDALSHVNAMIGDKIPEFNWEFMKPSKAPISSKKYAFDIL

RAFYHLYKYRDGFSIAKIETKKLVMRTVLDPVPM

>Sl_copalyl_diphosphate_syn

MSISASFLRFSLTAHYQPSPSSSPPNQPFKFLKSNREHVEFNRILQCHAVSRRRTKDYKE

VQSGSLPVIKWDDIAEEVDEETHTLEVYDPSSNEDHIDAIRSMLGSMGDGEISVSAYDTA

WVAMVKDVKGTETPQFPSSLEWIANNQLADGSWGDNSIFLVYDRVINTLACVIALKSWNL

HPDKILLGMSFMRENLSRIGDENAEHMPIGFEVAFPSLIEIAKKLGLDFPYDSPVLQDIY

ASRQLKLTRIPKDIMHKVPTTLLHSLEGMTDLDWQKLLQFQCTDGSFLFSPSSTAYALMQ

TQDHNCLNYLKNAVHKFNGGVPNVYPVDLFEHIWTVDRLQRLGISRYFELKIKKCIDYFS

KYWTNKGICWARNSPVQDIDDTAMAFRLLRLHGYAVSADVFKHFESKGEFFCFVGQSNQA

VTGMYNLYRASHVMFSGEKILENAKISTSNYLREKRAQNQLLDKWIITKDLPGEVGYALD

VPWYASLPRLETRFFLEHYGGEDDVWIGKTLYRMPLVNNSLYLELAKSDYNNCQALHQFE

WRRIRKWYYECGLREFGLSEKRLLVTYYLGSASIFEAQRSTERMAWVKTAALMDCVRSCF

GSPQVSAAAFLCEFAHYSSTALNSRYNTEDRLVGVILGTLNHLSLSALLTHGRDIHHYLR

HAWENWLLTVGEGEGEGEGGAELIIRTLNLCSVHWISEEILLSHPTYQKLLEITNRVSHR

LRLYKGHSEKQVGMLTFSEEIEGDMQQLAELVLSHSDASELDANIKDTFLTVAKSFYYSA

YCDDRTINFHIAKVLFERVV

>So_(+)-bornyl_diphosphate_syn

MSIISMNVSILSKPLNCLHNLERRPSKALLVPCTAPTARLRASCSSKLQEAHQIRRSGNY

QPALWDSNYIQSLNTPYTEERHLDRKAELIVQVRILLKEKMEPVQQLELIHDLKYLGLSD

FFQDEIKEILGVIYNEHKCFHNNEVEKMDLYFTALGFRLLRQHGFNISQDVFNCFKNEKG

IDFKASLAQDTKGMLQLYEASFLLRKGEDTLELAREFATKCLQKKLDEGGNEIDENLLLW

IRHSLDLPLHWRIQSVEARWFIDAYARRPDMNPLIFELAKLNFNIIQATHQQELKDLSRW

WSRLCFPEKLPFVRDRLVESFFWAVGMFEPHQHGYQRKMAATIIVLATVIDDIYDVYGTL

DELELFTDTFKRWDTESITRLPYYMQLCYWGVHNYISDAAYDILKEHGFFCLQYLRKSVV

DLVEAYFHEAKWYHSGYTPSLDEYLNIAKISVASPAIISPTYFTFANASHDTAVIDSLYQ

YHDILCLAGIILRLPDDLGTSYFELARGDVPKTIQCYMKETNASEEEAVEHVKFLIREAW

KDMNTAIAAGYPFPDGMVAGAANIGRVAQFIYLHGDGFGVQHSKTYEHIAGLLFEPYA

>So_(+)-sabinene_syn

MSSISINIAMPLNSLHNFERKPSKAWSTSCTAPAARLRASSSLQQEKPHQIRRSGDYQPS

LWDFNYIQSLNTPYKEQRHFNRQAELIMQVRMLLKVKMEAIQQLELIDDLQYLGLSYFFQ

DEIKQILSSIHNEPRYFHNNDLYFTALGFRILRQHGFNVSEDVFDCFKIEKCSDFNANLA

QDTKGMLQLYEASFLLREGEDTLELARRFSTRSLREKFDEGGDEIDEDLSSWIRHSLDLP

LHWRVQGLEARWFLDAYARRPDMNPLIFKLAKLNFNIVQATYQEELKDISRWWNSSCLAE

KLPFVRDRIVECFFWAIAAFEPHQYSYQRKMAAVIITFITIIDDVYDVYGTIEELELLTD

MIRRWDNKSISQLPYYMQVCYLALYNFVSERAYDILKDQHFNSIPYLQRSWVSLVEGYLK

EAYWYYNGYKPSLEEYLNNAKISISAPTIISQLYFTLANSIDETAIESLYQYHNILYLSG

TILRLADDLGTSQHELERGDVPKAIQCYMNDTNASEREAVEHVKFLIREAWKEMNTVTTA

SDCPFTDDLVAAAANLARAAQFIYLDGDGHGVQHSEIHQQMGGLLFQPYV

>So_1.8-cineole_syn

MSSLIMQVVIPKPAKIFHNNLFSVISKRHRFSTTITTRGGRWAHCSLQMGNEIQTGRRTG

GYQPTLWDFSTIQLFDSEYKEEKHLMRAAGMIAQVNMLLQEEVDSIQRLELIDDLRRLGI

SCHFDREIVEILNSKYYTNNEIDESDLYSTALRFKLLRQYDFSVSQEVFDCFKNDKGTDF

KPSLVDDTRGLLQLYEASFLSAQGEETLHLARDFATKFLHKRVLVDKDINLLSSIERALE

LPTHWRVQMPNARSFIDAYKRRPDMNPTVLELAKLDFNMVQAQFQQELKEASRWWNSTGL

VHELPFVRDRIVECYYWTTGVVERREHGYERIMLTKINALVTTIDDVFDIYGTLEELQLF

TTAIQRWDIESMKQLPPYMQICYLALFNFVNEMAYDTLRDKGFNSTPYLRKAWVDLVESY

LIEAKWYYMGHKPSLEEYMKNSWISIGGIPILSHLFFRLTDSIEEEDAESMHKYHDIVRA

SCTILRLADDMGTSLDEVERGDVPKSVQCYMNEKNASEEEAREHVRSLIDQTWKMMNKEM

MTSSFSKYFVQVSANLARMAQWIYQHESDGFGMQHSLVNKMLRGLLFDRYE

>St_(+)-4R-limonene_syn

MALKMTSAVMQMAIPTKLANFVNNSDTHKQSLKLLRNVSTISTSAAAATPRHRLPVCCSA

SSSSSSQLPTIERRSGNYKPSRWDVDFMQSLNSDYQEERHRTKASELITQVKNLLEKETS

DDPIRQLELIDDLQRLGLSDHFEHEFKEVLNSIYLDNKYYNINIMKETTSSRDLYSTALA

FRLLREHGFQVAQEVFDCFKNEEGEFKASLSDDPRGLLQLYEASFLFKEGENTLEIAREF

ATKLLQEKVNSSDEIDDNLLSSIRYSLEIPTYWSVIRPNVSVWIDAYRKRPDMNPVVLEL

AILDANIMQAQLQQELKEALGWWRNTWFVEKLPFARDRLVESYFWSTGMVPRRQHKTARQ

LMAKVIALITVMDDIYDVYGTLEELELFTDAFRRWDVSSIDHLPTYMQLCFLSINNFVVD

TAYNILKETGVNVTTYLEKSWVDQAENYLMESKWFYSGHKPSLDEYLENSWISVSGPCVL

THEFFGVTDSLAKDTLDSLYEYHDIVRWSSYLLRLADDLGTSVEEVSRGDVPKSIQCYMN

DNNASEEEAREHVKGLIRVMWKKMNAERVSEDSPFCKDFIRCCEDLGRMAQFMYHYGDGH

GTQHAKIHQQITDCLFQPFA

>St_3-carene_syn

MAFPRNPTKLLHKPHNKSSKLISNSRISSYGHLPLRCSSQQLPTDEFQVERRSGNYSPSK

WDVDYIQSLHSDYKEERHTRRASELIMEVKKLLEKEPNPTRQLELIDDLQKLGLSDHFNN

EFKEILNSVYLDNKYYRNGAMKEVERDLYSTALAFRLLRQHGFQVAQDVLECFKNTKGEF

EPSLSDDTRGLLQLYEASFLLTEGENTLELARDFTTKILEEKLRNDEIDDINLVTWIRHS

LEIPIHWRIDRVNTSVWIDVYKRRPDMNPIVLELAVLDSNIVQAQYQEELKLDLQWWRNT

CLAEKLPFARDRLVESYFWGVGVVQPRQHGIARMAVDRSIALITVIDDVYDVYGTLEELE

QFTEAIRRWDISSIDQLPSYMQLCFLALDNFINDIAYDVLKEQGFNIIPYLRKSWTDMIE

GFLLEAKWYHNGHKPKLEEYLENGWRSIGSTVVLTHAFFGVTHSLTKENIDQFFGYHEIV

RLSSMLLRLADDLGTSTDEVSRGDVPKAIQCYMNDNIGASEAEAREHVKWCIWETWKKMN

KVRVARDTPFSQDFIVCAMGMGRMGQYMYHYGDGHGIQHSIIHQQMSTCLFHPSSSN

>Wv_bornyl_diphosphate_syn

MATRQVTSIYAFPMISVLPRRPMIVTAVEHRGRQTFRRTLQVRSCIATSNVAPLRRSGNY

PQNIWTDERVQSLTSTSTEQREEKRERRNVLKEQTRNLILEQQQVAEQLRLIDHLQQLGV

AYHFKDEISDVLSRLHASLDGVSSQLEDDLHATALLFRLLRANGFSVSQDLFETFRDEKG

NFEVRCEDQIRGLLSLYEASYLEKEGEILLKEAMDFATDKLKGFMEEGSGSLGLREQVAH

ALQLPLNWRMERVQHRWFIEACNGADDAINPLLLEFAKLDYNLVQDMYKSELRELSSWWS

GLGLLEKLPFFRDRLAENYLWAAGFAYEPDSWRCRMIQTKIICLVTMIDDIYDVYGTLDE

LQLFTDVVDRWDLTAMDKLPEYMKLCFFALFNMVHEEGYRVMKEKGLDIVPDLKRIWGNQ

CKSYLKEAKWFHHGQIPTLEEYLENGYVSVTTPMVLLHALCAGQDLTGEALKSFSSYYAI

TRSTGMLFRLYDDMGTSTDEIERGDVAKCIQCYMHEKGVTEEAARKEMTGLMRKYWRESN

GYLSWNSPVEEYLKNVAINIPRTAQFFYLYGDGYGMVVDRETKSQIISLFLEPIQI

>Wv_pinene_syn

MSVSLSSAASATFGSRAGIGGSGSRSAAAIKRRRRLPRIQCHAAEESQSLSSTTSRRSGN

YQPSIWTHDRIQSLTLSHAAADEEDHAEEIKLLKYQTSKLMEEKKGRVEEQLQLIDHLQQ

LGVAYHFKDEIKDTLRGFHASFEDVSLQLRDNLHASALLFRLLRENGFSVSEDIFKKFKD

EKAGQFEDRLQSQAEGLLSLYEASYLEKDGEELLHEAREFTTKHLKNLLEEEGSLKPGLI

REQVAHALELPLNWRFQRLHTKWFIGAWQRDPAMDPALLGLAKLDFNALQNIYKRELKEA

SRWWTDLGLPQKLPFFRDRLTENYLWTVGWAFEPDSWAFRELQTKINCFITLIDDVYDVY

GTLDELELFTDIMERWDVNSIDKLPEYMKICFLAVFNTVNDAGYEVIRDKGVDIIPYLKR

AWAELCKMYMREARWYHAGYTPTLDEYLDGAWISISGALILSTAYCMGNDLTKDDLDKFS

TYPAVMHPSCMLLRLHDDFGTSTDELARGDVQKAVQCCMHERKVPEAVAREHIMQVMEAK

WRLLNGNRVATSSFEEYFLNVAINIPRSAQFFYGQGDGYGKSDGETKKQVISLLIEPVQF
