# Supplementary material for: Metabolism and transcriptome profiling provides insight into the genes and transcription factors involved in monoterpene biosynthesis of borneol chemotype of Cinnamomum camphora induced by mechanical damage
Source: PeerJ. 2021 Jul 1;9:e11465. doi: 10.7717/peerj.11465 (PMC8255067; doi:10.7717/peerj.11465)
Supplement: Supplemental Information 13 [file peerj-09-11465-s013.docx]

| **TF family** | **Number** |
| --- | --- |
| MYB_superfamily | 127 |
| AP2/ERF | 81 |
| bHLH | 55 |
| C2C2 | 53 |
| C2H2 | 48 |
| NAC | 42 |
| WRKY | 37 |
| bZIP | 35 |
| LBD (AS2/LOB) | 35 |
| GRAS | 23 |
| TCP | 19 |
| B3_superfamily | 18 |
| HSF | 15 |
| C3H | 13 |
| MADS | 13 |
| LOB | 11 |
| NF-Y | 7 |
| SBP | 6 |
| FAR1 | 5 |
| Nin-like | 5 |
| ZF-HD | 5 |
| BES1 | 4 |
| EIL | 4 |
| E2F/DP | 3 |
| BBR-BPC | 2 |
| CPP | 2 |
| GeBP | 2 |
| CAMTA | 1 |
| S1Fa-like | 1 |
| SRS | 1 |
