# Supplementary material for: Metabolism and transcriptome profiling provides insight into the genes and transcription factors involved in monoterpene biosynthesis of borneol chemotype of Cinnamomum camphora induced by mechanical damage
Source: PeerJ. 2021 Jul 1;9:e11465. doi: 10.7717/peerj.11465 (PMC8255067; doi:10.7717/peerj.11465)
Supplement: Supplemental Information 14 [file peerj-09-11465-s014.docx]

| **Gene_id** | **Rename** | **Description** | **FPKM** | | |
| --- | --- | --- | --- | --- | --- |
|  |  |  | **CK** | **MD_2h** | **MD_6h** |
| TRINITY_DN45628_c2_g11 | CcMYB1 | Myb-like DNA-binding domain | 0.15 | 0.71 | 0.65 |
| TRINITY_DN44798_c2_g4 | CcMYB2 | Myb-like DNA-binding domain | 0.00 | 25.39 | 6.22 |
| TRINITY_DN49151_c1_g1 | CcMYB3 | Myb-like DNA-binding domain | 17.21 | 18.20 | 3.16 |
| TRINITY_DN50766_c3_g1 | CcMYB4 | Myb-like DNA-binding domain | 1.73 | 0.22 | 2.77 |
| TRINITY_DN31698_c0_g1 | CcMYB5 | Myb-like DNA-binding domain | 29.63 | 11.38 | 3.28 |
| TRINITY_DN40236_c0_g1 | CcMYB6 | Myb-like DNA-binding domain | 42.64 | 6.38 | 9.81 |
| TRINITY_DN50575_c2_g1 | CcMYB7 | Myb-like DNA-binding domain | 5.60 | 1.30 | 8.34 |
| TRINITY_DN42715_c0_g3 | CcMYB8 | Myb-like DNA-binding domain | 7.91 | 138.32 | 79.28 |
| TRINITY_DN42715_c1_g1 | CcMYB9 | Myb-like DNA-binding domain | 7.10 | 3.65 | 5.53 |
| TRINITY_DN49154_c3_g1 | CcMYB10 | Myb-like DNA-binding domain | 0.00 | 0.66 | 1.09 |
| TRINITY_DN49195_c1_g1 | CcMYB11 | Myb-like DNA-binding domain | 11.30 | 6.51 | 5.06 |
| TRINITY_DN49195_c2_g2 | CcMYB12 | Myb-like DNA-binding domain | 18.24 | 7.99 | 9.97 |
| TRINITY_DN47421_c0_g1 | CcMYB13 | Myb-like DNA-binding domain | 6.70 | 2.08 | 3.24 |
| TRINITY_DN48475_c1_g1 | CcMYB14 | Myb-like DNA-binding domain | 0.03 | 0.68 | 0.78 |
| TRINITY_DN50385_c2_g1 | CcMYB15 | Myb-like DNA-binding domain | 0.05 | 0.75 | 1.00 |
| TRINITY_DN36467_c0_g1 | CcMYB16 | Myb-like DNA-binding domain | 3.26 | 3.44 | 1.88 |
| TRINITY_DN45791_c3_g2 | CcMYB17 | Myb-like DNA-binding domain | 40.35 | 26.96 | 65.27 |
| TRINITY_DN46797_c4_g1 | CcMYB18 | Myb-like DNA-binding domain | 2.11 | 2.74 | 6.03 |
| TRINITY_DN46797_c4_g3 | CcMYB19 | Myb-like DNA-binding domain | 2.66 | 1.59 | 6.88 |
| TRINITY_DN51230_c1_g1 | CcMYB20 | Myb-like DNA-binding domain | 31.43 | 12.94 | 12.89 |
| TRINITY_DN51230_c1_g8 | CcMYB21 | Myb-like DNA-binding domain | 0.45 | 0.09 | 1.42 |
| TRINITY_DN48168_c0_g1 | CcMYB22 | Myb-like DNA-binding domain | 6.66 | 6.72 | 3.68 |
| TRINITY_DN48168_c1_g1 | CcMYB23 | Myb-like DNA-binding domain | 6.38 | 7.01 | 27.57 |
| TRINITY_DN48168_c4_g2 | CcMYB24 | Myb-like DNA-binding domain | 6.34 | 5.66 | 95.65 |
| TRINITY_DN43012_c0_g1 | CcMYB25 | Myb-like DNA-binding domain | 0.05 | 0.19 | 1.83 |
| TRINITY_DN45267_c2_g3 | CcMYB26 | Myb-like DNA-binding domain | 0.69 | 25.60 | 12.74 |
| TRINITY_DN51412_c3_g1 | CcMYB27 | Myb-like DNA-binding domain | 15.25 | 30.64 | 6.46 |
| TRINITY_DN43639_c1_g1 | CcMYB28 | Myb-like DNA-binding domain | 39.32 | 20.74 | 20.89 |
| TRINITY_DN43639_c1_g6 | CcMYB29 | Myb-like DNA-binding domain | 3.06 | 6.96 | 0.67 |
| TRINITY_DN43639_c1_g5 | CcMYB30 | Myb-like DNA-binding domain | 0.71 | 0.25 | 0.18 |
| TRINITY_DN41214_c1_g2 | CcMYB31 | Myb-like DNA-binding domain | 5.85 | 2.06 | 3.72 |
| TRINITY_DN41272_c0_g1 | CcMYB32 | Myb-like DNA-binding domain | 1.42 | 41.37 | 37.35 |
| TRINITY_DN25581_c0_g1 | CcMYB33 | Myb-like DNA-binding domain | 1.31 | 2.18 | 0.71 |
| TRINITY_DN43044_c0_g2 | CcMYB34 | Myb-like DNA-binding domain | 0.35 | 2.27 | 0.93 |
| TRINITY_DN44712_c1_g1 | CcMYB35 | Myb-like DNA-binding domain | 2.09 | 2.00 | 0.31 |
| TRINITY_DN44713_c0_g1 | CcMYB36 | Myb-like DNA-binding domain | 268.83 | 269.04 | 93.44 |
| TRINITY_DN44707_c0_g1 | CcMYB37 | Myb-like DNA-binding domain | 39.39 | 32.08 | 21.13 |
| TRINITY_DN44728_c7_g1 | CcMYB38 | Myb-like DNA-binding domain | 0.20 | 0.12 | 1.00 |
| TRINITY_DN47529_c4_g2 | CcMYB39 | Myb-like DNA-binding domain | 75.67 | 51.44 | 29.41 |
| TRINITY_DN49235_c1_g1 | CcMYB40 | Myb-like DNA-binding domain | 0.36 | 1.11 | 7.46 |
| TRINITY_DN49627_c1_g2 | CcMYB41 | Myb-like DNA-binding domain | 3.07 | 18.72 | 12.32 |
| TRINITY_DN50844_c0_g2 | CcMYB42 | Myb-like DNA-binding domain | 46.35 | 51.74 | 25.52 |
| TRINITY_DN46101_c2_g7 | CcMYB43 | Myb-like DNA-binding domain | 40.50 | 6.68 | 34.04 |
| TRINITY_DN41900_c0_g1 | CcMYB44 | Myb-like DNA-binding domain | 2.13 | 5.56 | 3.68 |
| TRINITY_DN48422_c1_g4 | CcMYB45 | Myb-like DNA-binding domain | 7.39 | 4.52 | 3.87 |
| TRINITY_DN50238_c2_g2 | CcMYB46 | Myb-like DNA-binding domain | 11.53 | 19.77 | 13.40 |
| TRINITY_DN46238_c1_g1 | CcMYB47 | Myb-like DNA-binding domain | 26.64 | 26.01 | 8.72 |
| TRINITY_DN39982_c0_g1 | CcMYB48 | Myb-like DNA-binding domain | 3.54 | 2.01 | 3.38 |
| TRINITY_DN43622_c2_g1 | CcMYB49 | Myb-like DNA-binding domain | 0.00 | 0.57 | 0.70 |
| TRINITY_DN43663_c0_g1 | CcMYB50 | Myb-like DNA-binding domain | 13.64 | 14.80 | 74.34 |
| TRINITY_DN43571_c1_g5 | CcMYB51 | Myb-like DNA-binding domain | 1.09 | 1.62 | 4.53 |
| TRINITY_DN37951_c0_g1 | CcMYB52 | Myb-like DNA-binding domain | 0.17 | 1.96 | 6.48 |
| TRINITY_DN47044_c1_g1 | CcMYB53 | Myb-like DNA-binding domain | 0.05 | 0.38 | 1.27 |
| TRINITY_DN50733_c0_g2 | CcMYB54 | Myb-like DNA-binding domain | 0.89 | 12.46 | 30.56 |
| TRINITY_DN43903_c2_g1 | CcMYB55 | Myb-like DNA-binding domain | 111.86 | 76.58 | 24.47 |
| TRINITY_DN46417_c6_g1 | CcMYB56 | Myb-like DNA-binding domain | 2.40 | 7.66 | 16.43 |
| TRINITY_DN44150_c0_g3 | CcMYB57 | Myb-like DNA-binding domain | 3.32 | 2.46 | 5.50 |
| TRINITY_DN47613_c0_g1 | CcMYB58 | Myb-like DNA-binding domain | 1.52 | 2.35 | 0.95 |
| TRINITY_DN46626_c1_g1 | CcMYB59 | Myb-like DNA-binding domain | 3.70 | 7.79 | 3.08 |
| TRINITY_DN46626_c1_g1 | CcMYB60 | Myb-like DNA-binding domain | 19.34 | 5.38 | 19.16 |
| TRINITY_DN44653_c5_g2 | CcMYB61 | Myb-like DNA-binding domain | 75.09 | 15.81 | 24.62 |
| TRINITY_DN42859_c3_g4 | CcMYB62 | Myb-like DNA-binding domain | 345.38 | 109.39 | 94.71 |
| TRINITY_DN42859_c3_g1 | CcMYB63 | Myb-like DNA-binding domain | 15.37 | 62.49 | 97.72 |
| TRINITY_DN48132_c1_g2 | CcMYB64 | Myb-like DNA-binding domain | 2.51 | 4.74 | 1.78 |
| TRINITY_DN47090_c1_g1 | CcMYB65 | Myb-like DNA-binding domain | 61.63 | 20.26 | 24.29 |
| TRINITY_DN47090_c1_g1 | CcMYB66 | Myb-like DNA-binding domain | 8.76 | 2.81 | 23.78 |
| TRINITY_DN47090_c1_g2 | CcMYB67 | Myb-like DNA-binding domain | 0.33 | 0.21 | 2.05 |
| TRINITY_DN47090_c1_g4 | CcMYB68 | Myb-like DNA-binding domain | 0.10 | 108.87 | 24.82 |
| TRINITY_DN47090_c1_g3 | CcMYB69 | Myb-like DNA-binding domain | 3.55 | 7.72 | 13.65 |
| TRINITY_DN31376_c0_g1 | CcMYB70 | Myb-like DNA-binding domain | 46.77 | 21.56 | 29.95 |
| TRINITY_DN49768_c1_g4 | CcMYB71 | Myb-like DNA-binding domain | 14.69 | 12.93 | 8.83 |
| TRINITY_DN49787_c0_g1 | CcMYB72 | Myb-like DNA-binding domain | 12.16 | 13.62 | 5.09 |
| TRINITY_DN43465_c2_g3 | CcMYB73 | Myb-like DNA-binding domain | 7.85 | 6.24 | 0.81 |
| TRINITY_DN43465_c2_g1 | CcMYB74 | Myb-like DNA-binding domain | 9.34 | 6.79 | 2.19 |
| TRINITY_DN47910_c1_g2 | CcMYB75 | Myb-like DNA-binding domain | 192.87 | 67.31 | 43.87 |
| TRINITY_DN46359_c3_g6 | CcMYB76 | Myb-like DNA-binding domain | 24.03 | 5.50 | 3.53 |
| TRINITY_DN40317_c0_g1 | CcMYB77 | Myb-like DNA-binding domain | 0.05 | 1.83 | 2.52 |
| TRINITY_DN43933_c1_g1 | CcMYB78 | Myb-like DNA-binding domain | 120.92 | 22.97 | 56.86 |
| TRINITY_DN44036_c1_g6 | CcMYB79 | Myb-like DNA-binding domain | 10.53 | 3.71 | 6.34 |
| TRINITY_DN49926_c0_g1 | CcMYB80 | Myb-like DNA-binding domain | 9.58 | 19.42 | 5.05 |
| TRINITY_DN36663_c0_g2 | CcMYB81 | Myb-like DNA-binding domain | 0.35 | 12.31 | 47.34 |
| TRINITY_DN42710_c1_g2 | CcMYB82 | Myb-like DNA-binding domain | 10.13 | 44.97 | 16.44 |
| TRINITY_DN43422_c1_g9 | CcMYB83 | Myb-like DNA-binding domain | 0.77 | 15.86 | 4.26 |
| TRINITY_DN47700_c0_g1 | CcMYB84 | Myb-like DNA-binding domain | 0.61 | 24.82 | 88.55 |
| TRINITY_DN47700_c1_g4 | CcMYB85 | Myb-like DNA-binding domain | 0.55 | 3.00 | 30.16 |
| TRINITY_DN47700_c1_g6 | CcMYB86 | Myb-like DNA-binding domain | 0.38 | 4.94 | 5.47 |
| TRINITY_DN47700_c1_g1 | CcMYB87 | Myb-like DNA-binding domain | 0.72 | 2.35 | 2.51 |
| TRINITY_DN44045_c1_g7 | CcMYB88 | Myb-like DNA-binding domain | 24.62 | 105.46 | 34.87 |
| TRINITY_DN48393_c2_g1 | CcMYB89 | Myb-like DNA-binding domain | 2.23 | 2.58 | 9.89 |
| TRINITY_DN48393_c3_g4 | CcMYB90 | Myb-like DNA-binding domain | 1.01 | 3.22 | 2.25 |
| TRINITY_DN48393_c3_g2 | CcMYB91 | Myb-like DNA-binding domain | 3.80 | 0.60 | 7.82 |
| TRINITY_DN50435_c0_g1 | CcMYB92 | Myb-like DNA-binding domain | 0.83 | 3.53 | 19.99 |
| TRINITY_DN44375_c1_g3 | CcMYB93 | Myb-like DNA-binding domain | 0.34 | 1.95 | 3.12 |
| TRINITY_DN37633_c0_g1 | CcMYB94 | Myb-like DNA-binding domain | 0.88 | 129.47 | 35.34 |
| TRINITY_DN48353_c2_g3 | CcMYB95 | Myb-like DNA-binding domain | 0.05 | 5.03 | 0.57 |
| TRINITY_DN48353_c2_g4 | CcMYB96 | Myb-like DNA-binding domain | 3.40 | 45.36 | 22.60 |
| TRINITY_DN45916_c5_g1 | CcMYB97 | Myb-like DNA-binding domain | 0.36 | 5.38 | 2.74 |
| TRINITY_DN42397_c0_g1 | CcMYB98 | Myb-like DNA-binding domain | 94.90 | 145.53 | 75.03 |
| TRINITY_DN48049_c1_g1 | CcMYB99 | Myb-like DNA-binding domain | 10.96 | 22.75 | 30.62 |
| TRINITY_DN48054_c1_g1 | CcMYB100 | Myb-like DNA-binding domain | 0.59 | 0.35 | 1.86 |
| TRINITY_DN48054_c2_g2 | CcMYB101 | Myb-like DNA-binding domain | 12.79 | 6.28 | 4.05 |
| TRINITY_DN48054_c2_g3 | CcMYB102 | Myb-like DNA-binding domain | 5.66 | 5.12 | 1.90 |
| TRINITY_DN48054_c2_g7 | CcMYB103 | Myb-like DNA-binding domain | 0.36 | 55.92 | 17.08 |
| TRINITY_DN48054_c2_g8 | CcMYB104 | Myb-like DNA-binding domain | 16.79 | 6.35 | 14.42 |
| TRINITY_DN47691_c1_g6 | CcMYB105 | Myb-like DNA-binding domain | 18.18 | 19.49 | 8.81 |
| TRINITY_DN47691_c1_g5 | CcMYB106 | Myb-like DNA-binding domain | 2.83 | 6.97 | 5.77 |
| TRINITY_DN50985_c1_g4 | CcMYB107 | Myb-like DNA-binding domain | 37.60 | 21.92 | 58.73 |
| TRINITY_DN49375_c0_g5 | CcMYB108 | Myb-like DNA-binding domain | 0.48 | 1.53 | 0.21 |
| TRINITY_DN41558_c0_g1 | CcMYB109 | Myb-like DNA-binding domain | 3.74 | 2.49 | 1.18 |
| TRINITY_DN45409_c0_g1 | CcMYB110 | Myb-like DNA-binding domain | 50.45 | 145.89 | 292.68 |
| TRINITY_DN45409_c0_g2 | CcMYB111 | Myb-like DNA-binding domain | 4.14 | 3.40 | 2.61 |
| TRINITY_DN45409_c0_g9 | CcMYB112 | Myb-like DNA-binding domain | 6.09 | 4.37 | 0.50 |
| TRINITY_DN45451_c2_g1 | CcMYB113 | Myb-like DNA-binding domain | 71.44 | 21.51 | 30.21 |
| TRINITY_DN50794_c1_g1 | CcMYB114 | Myb-like DNA-binding domain | 304.71 | 243.80 | 45.60 |
| TRINITY_DN34173_c0_g1 | CcMYB115 | Myb-like DNA-binding domain | 9.41 | 23.36 | 12.09 |
| TRINITY_DN44521_c0_g1 | CcMYB116 | Myb-like DNA-binding domain | 35.19 | 80.23 | 61.72 |
| TRINITY_DN38902_c0_g1 | CcMYB117 | Myb-like DNA-binding domain | 6.25 | 19.95 | 24.40 |
| TRINITY_DN51074_c8_g1 | CcMYB118 | Myb-like DNA-binding domain | 0.03 | 0.38 | 0.98 |
| TRINITY_DN42970_c0_g1 | CcMYB119 | Myb-like DNA-binding domain | 54.81 | 18.23 | 28.26 |
| TRINITY_DN44966_c2_g4 | CcMYB120 | Myb-like DNA-binding domain | 3.28 | 70.42 | 70.35 |
| TRINITY_DN48238_c0_g1 | CcMYB121 | Myb-like DNA-binding domain | 1.32 | 3.45 | 0.89 |
| TRINITY_DN48238_c0_g3 | CcMYB122 | Myb-like DNA-binding domain | 1.21 | 4.13 | 0.70 |
| TRINITY_DN48227_c3_g1 | CcMYB123 | Myb-like DNA-binding domain | 66.49 | 7.48 | 48.73 |
| TRINITY_DN48227_c3_g5 | CcMYB124 | Myb-like DNA-binding domain | 17.98 | 7.10 | 12.28 |
| TRINITY_DN45569_c2_g1 | CcMYB125 | Myb-like DNA-binding domain | 4.60 | 2.88 | 0.80 |
| TRINITY_DN19910_c0_g1 | CcMYB126 | Myb-like DNA-binding domain | 6.25 | 19.95 | 24.40 |
| TRINITY_DN40431_c0_g1 | CcMYB127 | Myb-like DNA-binding domain | 0.03 | 0.38 | 0.98 |
| TRINITY_DN35704_c0_g1 | CcWRKY1 | WRKY DNA -binding domain | 4.56 | 1.13 | 3.34 |
| TRINITY_DN45791_c4_g1 | CcWRKY2 | WRKY DNA -binding domain | 0.18 | 0.67 | 0.73 |
| TRINITY_DN46744_c1_g5 | CcWRKY3 | WRKY DNA -binding domain | 0.76 | 5.12 | 2.85 |
| TRINITY_DN50241_c3_g3 | CcWRKY4 | WRKY DNA -binding domain | 1.91 | 1.89 | 0.53 |
| TRINITY_DN51409_c2_g3 | CcWRKY5 | WRKY DNA -binding domain | 0.03 | 0.30 | 3.82 |
| TRINITY_DN51409_c2_g9 | CcWRKY6 | WRKY DNA -binding domain | 2.40 | 6.15 | 3.71 |
| TRINITY_DN45494_c1_g4 | CcWRKY7 | WRKY DNA -binding domain | 0.31 | 2.96 | 1.93 |
| TRINITY_DN48629_c0_g1 | CcWRKY8 | WRKY DNA -binding domain | 108.09 | 927.99 | 128.19 |
| TRINITY_DN48629_c0_g7 | CcWRKY9 | WRKY DNA -binding domain | 1.12 | 5.49 | 1.45 |
| TRINITY_DN48228_c0_g10 | CcWRKY10 | WRKY DNA -binding domain | 0.51 | 0.26 | 7.09 |
| TRINITY_DN49750_c0_g3 | CcWRKY11 | WRKY DNA -binding domain | 6.25 | 18.25 | 51.92 |
| TRINITY_DN22541_c0_g2 | CcWRKY12 | WRKY DNA -binding domain | 0.04 | 6.05 | 1.12 |
| TRINITY_DN46259_c0_g4 | CcWRKY13 | WRKY DNA -binding domain | 1.96 | 32.14 | 11.11 |
| TRINITY_DN46587_c2_g1 | CcWRKY14 | WRKY DNA -binding domain | 9.18 | 35.00 | 57.59 |
| TRINITY_DN46572_c2_g1 | CcWRKY15 | WRKY DNA -binding domain | 9.16 | 8.08 | 26.18 |
| TRINITY_DN38622_c0_g1 | CcWRKY16 | WRKY DNA -binding domain | 7.32 | 2.43 | 3.74 |
| TRINITY_DN49545_c1_g1 | CcWRKY17 | WRKY DNA -binding domain | 8.00 | 322.41 | 137.58 |
| TRINITY_DN44317_c2_g1 | CcWRKY18 | WRKY DNA -binding domain | 6.36 | 15.36 | 17.62 |
| TRINITY_DN48296_c3_g1 | CcWRKY19 | WRKY DNA -binding domain | 1.07 | 0.48 | 0.54 |
| TRINITY_DN39627_c0_g2 | CcWRKY20 | WRKY DNA -binding domain | 37.85 | 10.67 | 10.86 |
| TRINITY_DN38119_c0_g1 | CcWRKY21 | WRKY DNA -binding domain | 2.40 | 37.37 | 118.20 |
| TRINITY_DN45810_c3_g4 | CcWRKY22 | WRKY DNA -binding domain | 25.96 | 293.60 | 222.89 |
| TRINITY_DN48306_c1_g3 | CcWRKY23 | WRKY DNA -binding domain | 1.18 | 219.66 | 135.36 |
| TRINITY_DN43541_c3_g1 | CcWRKY24 | WRKY DNA -binding domain | 59.45 | 232.01 | 109.07 |
| TRINITY_DN43541_c3_g2 | CcWRKY25 | WRKY DNA -binding domain | 2.35 | 1.74 | 8.81 |
| TRINITY_DN43541_c3_g3 | CcWRKY26 | WRKY DNA -binding domain | 3.01 | 55.75 | 54.98 |
| TRINITY_DN46028_c0_g1 | CcWRKY27 | WRKY DNA -binding domain | 0.40 | 1.30 | 0.69 |
| TRINITY_DN42888_c0_g1 | CcWRKY28 | WRKY DNA -binding domain | 5.09 | 7.45 | 36.99 |
| TRINITY_DN46617_c0_g1 | CcWRKY29 | WRKY DNA -binding domain | 4.96 | 3.75 | 8.58 |
| TRINITY_DN48000_c1_g1 | CcWRKY30 | WRKY DNA -binding domain | 39.25 | 306.36 | 215.00 |
| TRINITY_DN48000_c1_g1 | CcWRKY31 | WRKY DNA -binding domain | 7.12 | 91.05 | 38.62 |
| TRINITY_DN43812_c0_g7 | CcWRKY32 | WRKY DNA -binding domain | 24.58 | 79.33 | 25.41 |
| TRINITY_DN45566_c3_g1 | CcWRKY33 | WRKY DNA -binding domain | 37.45 | 625.83 | 225.35 |
| TRINITY_DN44923_c1_g2 | CcWRKY34 | WRKY DNA -binding domain | 0.33 | 0.08 | 2.47 |
| TRINITY_DN44923_c1_g7 | CcWRKY35 | WRKY DNA -binding domain | 3.14 | 6.42 | 0.26 |
| TRINITY_DN44923_c1_g8 | CcWRKY36 | WRKY DNA -binding domain | 0.63 | 66.87 | 22.79 |
| TRINITY_DN44923_c1_g1 | CcWRKY37 | WRKY DNA -binding domain | 7.12 | 91.05 | 38.62 |
| TRINITY_DN49652_c0_g5 | CcbZIP1 | bZIP transcription factor | 0.15 | 0.13 | 0.85 |
| TRINITY_DN44798_c4_g2 | CcbZIP2 | bZIP transcription factor | 2.06 | 0.47 | 0.66 |
| TRINITY_DN40264_c2_g2 | CcbZIP3 | bZIP transcription factor | 0.42 | 0.04 | 0.65 |
| TRINITY_DN44382_c0_g1 | CcbZIP4 | bZIP transcription factor | 0.14 | 0.11 | 0.94 |
| TRINITY_DN36888_c0_g2 | CcbZIP5 | bZIP transcription factor | 0.00 | 0.21 | 1.11 |
| TRINITY_DN50241_c0_g1 | CcbZIP6 | bZIP transcription factor | 0.13 | 0.19 | 2.56 |
| TRINITY_DN51472_c2_g2 | CcbZIP7 | bZIP transcription factor | 0.31 | 0.12 | 0.83 |
| TRINITY_DN51472_c2_g6 | CcbZIP8 | bZIP transcription factor | 0.12 | 0.10 | 0.79 |
| TRINITY_DN43628_c0_g3 | CcbZIP9 | bZIP transcription factor | 4.62 | 1.80 | 2.60 |
| TRINITY_DN41214_c1_g1 | CcbZIP10 | bZIP transcription factor | 3.30 | 2.78 | 7.28 |
| TRINITY_DN47722_c0_g5 | CcbZIP11 | bZIP transcription factor | 12.35 | 19.16 | 7.74 |
| TRINITY_DN44701_c0_g1 | CcbZIP12 | bZIP transcription factor | 0.00 | 1.16 | 1.42 |
| TRINITY_DN46543_c0_g1 | CcbZIP13 | bZIP transcription factor | 1.85 | 31.81 | 1.80 |
| TRINITY_DN38629_c1_g1 | CcbZIP14 | bZIP transcription factor | 4.28 | 0.83 | 0.10 |
| TRINITY_DN48835_c1_g1 | CcbZIP15 | bZIP transcription factor | 26.07 | 32.29 | 14.31 |
| TRINITY_DN20038_c0_g1 | CcbZIP16 | bZIP transcription factor | 0.02 | 2.69 | 1.21 |
| TRINITY_DN47036_c3_g1 | CcbZIP17 | bZIP transcription factor | 3.80 | 24.97 | 9.23 |
| TRINITY_DN47036_c3_g2 | CcbZIP18 | bZIP transcription factor | 0.05 | 0.18 | 1.86 |
| TRINITY_DN51237_c1_g3 | CcbZIP19 | bZIP transcription factor | 0.05 | 3.84 | 2.85 |
| TRINITY_DN27565_c0_g2 | CcbZIP20 | bZIP transcription factor | 0.30 | 2.32 | 3.93 |
| TRINITY_DN38123_c0_g1 | CcbZIP21 | bZIP transcription factor | 1.31 | 7.96 | 11.11 |
| TRINITY_DN35256_c0_g1 | CcbZIP22 | bZIP transcription factor | 0.08 | 5.77 | 4.27 |
| TRINITY_DN48070_c0_g1 | CcbZIP23 | bZIP transcription factor | 0.04 | 1.76 | 1.68 |
| TRINITY_DN43569_c0_g1 | CcbZIP24 | bZIP transcription factor | 0.06 | 0.42 | 1.25 |
| TRINITY_DN44375_c1_g4 | CcbZIP25 | bZIP transcription factor | 1.87 | 0.44 | 2.32 |
| TRINITY_DN46043_c0_g1 | CcbZIP26 | bZIP transcription factor | 17.87 | 6.56 | 15.42 |
| TRINITY_DN50994_c6_g5 | CcbZIP27 | bZIP transcription factor | 29.63 | 147.90 | 74.92 |
| TRINITY_DN6958_c0_g1 | CcbZIP28 | bZIP transcription factor | 4.48 | 5.72 | 37.29 |
| TRINITY_DN48868_c0_g3 | CcbZIP29 | bZIP transcription factor | 0.96 | 10.81 | 2.59 |
| TRINITY_DN41548_c0_g1 | CcbZIP30 | bZIP transcription factor | 4.86 | 4.97 | 2.98 |
| TRINITY_DN37197_c0_g2 | CcbZIP31 | bZIP transcription factor | 2.14 | 0.48 | 0.78 |
| TRINITY_DN43356_c0_g1 | CcbZIP32 | bZIP transcription factor | 1.16 | 0.31 | 1.04 |
| TRINITY_DN45559_c0_g1 | CcbZIP33 | bZIP transcription factor | 1.67 | 5.28 | 10.26 |
| TRINITY_DN44555_c3_g1 | CcbZIP34 | bZIP transcription factor | 4.35 | 4.63 | 10.46 |
| TRINITY_DN46333_c2_g1 | CcbZIP35 | bZIP transcription factor | 0.64 | 1.28 | 0.32 |
| TRINITY_DN44233_c0_g1 | CcERF1 | AP2 domain | 0.00 | 1.08 | 0.56 |
| TRINITY_DN44036_c1_g2 | CcERF2 | AP2 domain | 0.75 | 0.72 | 0.17 |
| TRINITY_DN14404_c0_g1 | CcERF3 | AP2 domain | 0.06 | 0.73 | 0.78 |
| TRINITY_DN45121_c2_g3 | CcERF4 | AP2 domain | 8.46 | 30.40 | 25.00 |
| TRINITY_DN46170_c1_g1 | CcERF5 | AP2 domain | 0.13 | 131.89 | 58.94 |
| TRINITY_DN46170_c1_g2 | CcERF6 | AP2 domain | 1.39 | 30.87 | 29.64 |
| TRINITY_DN46170_c1_g3 | CcERF7 | AP2 domain | 0.00 | 0.25 | 1.23 |
| TRINITY_DN46170_c1_g4 | CcERF8 | AP2 domain | 0.65 | 2.66 | 5.18 |
| TRINITY_DN46170_c1_g5 | CcERF9 | AP2 domain | 0.02 | 14.95 | 9.53 |
| TRINITY_DN46170_c1_g6 | CcERF10 | AP2 domain | 0.00 | 73.99 | 18.29 |
| TRINITY_DN46170_c1_g7 | CcERF11 | AP2 domain | 4.31 | 15.99 | 15.16 |
| TRINITY_DN46170_c1_g8 | CcERF12 | AP2 domain | 32.03 | 138.82 | 103.33 |
| TRINITY_DN41203_c0_g2 | CcERF13 | AP2 domain | 0.35 | 169.46 | 66.90 |
| TRINITY_DN49918_c2_g2 | CcERF14 | AP2 domain | 0.19 | 131.25 | 72.43 |
| TRINITY_DN49918_c3_g1 | CcERF15 | AP2 domain | 0.00 | 6.25 | 2.87 |
| TRINITY_DN49246_c0_g3 | CcERF16 | AP2 domain | 0.15 | 2.33 | 1.55 |
| TRINITY_DN49246_c0_g1 | CcERF17 | AP2 domain | 2.22 | 3.94 | 5.65 |
| TRINITY_DN49246_c0_g4 | CcERF18 | AP2 domain | 0.82 | 41.21 | 159.80 |
| TRINITY_DN36480_c0_g1 | CcERF19 | AP2 domain | 0.88 | 7.03 | 7.58 |
| TRINITY_DN51227_c0_g3 | CcERF20 | AP2 domain | 0.02 | 125.71 | 79.02 |
| TRINITY_DN62408_c0_g1 | CcERF21 | AP2 domain | 31.29 | 316.47 | 55.25 |
| TRINITY_DN46791_c3_g5 | CcERF22 | AP2 domain | 65.09 | 588.47 | 505.10 |
| TRINITY_DN43658_c2_g9 | CcERF23 | AP2 domain | 8.46 | 166.29 | 93.83 |
| TRINITY_DN37494_c0_g1 | CcERF24 | AP2 domain | 3.82 | 253.61 | 117.33 |
| TRINITY_DN45270_c0_g3 | CcERF25 | AP2 domain | 3.85 | 36.40 | 13.50 |
| TRINITY_DN49492_c2_g7 | CcERF26 | AP2 domain | 1.22 | 313.08 | 73.03 |
| TRINITY_DN47880_c0_g2 | CcERF27 | AP2 domain | 0.00 | 11.15 | 6.15 |
| TRINITY_DN47880_c0_g3 | CcERF28 | AP2 domain | 0.02 | 9.45 | 2.10 |
| TRINITY_DN41971_c0_g1 | CcERF29 | AP2 domain | 9.56 | 6.44 | 3.32 |
| TRINITY_DN49708_c0_g12 | CcERF30 | AP2 domain | 1.78 | 0.57 | 0.48 |
| TRINITY_DN38017_c0_g1 | CcERF31 | AP2 domain | 0.51 | 0.26 | 1.51 |
| TRINITY_DN34248_c0_g1 | CcERF32 | AP2 domain | 0.13 | 49.60 | 16.71 |
| TRINITY_DN46054_c1_g1 | CcERF33 | AP2 domain | 0.00 | 30.05 | 1.36 |
| TRINITY_DN46575_c2_g1 | CcERF34 | AP2 domain | 0.00 | 6.79 | 0.33 |
| TRINITY_DN46575_c4_g1 | CcERF35 | AP2 domain | 0.14 | 41.15 | 3.61 |
| TRINITY_DN39424_c2_g1 | CcERF36 | AP2 domain | 0.00 | 17.86 | 4.11 |
| TRINITY_DN38620_c0_g1 | CcERF37 | AP2 domain | 0.13 | 5.94 | 107.08 |
| TRINITY_DN48853_c1_g3 | CcERF38 | AP2 domain | 3.27 | 68.26 | 84.29 |
| TRINITY_DN49514_c0_g1 | CcERF39 | AP2 domain | 0.32 | 3.67 | 3.27 |
| TRINITY_DN49514_c1_g1 | CcERF40 | AP2 domain | 2.16 | 3.42 | 5.90 |
| TRINITY_DN42179_c0_g1 | CcERF41 | AP2 domain | 16.87 | 3.91 | 7.72 |
| TRINITY_DN47289_c0_g1 | CcERF42 | AP2 domain | 6.47 | 1.40 | 13.22 |
| TRINITY_DN44650_c0_g1 | CcERF43 | AP2 domain | 1.64 | 0.92 | 2.33 |
| TRINITY_DN44650_c1_g1 | CcERF44 | AP2 domain | 0.93 | 6.27 | 9.29 |
| TRINITY_DN44650_c1_g2 | CcERF45 | AP2 domain | 3.21 | 315.14 | 47.66 |
| TRINITY_DN45626_c0_g1 | CcERF46 | AP2 domain | 0.41 | 15.50 | 37.53 |
| TRINITY_DN51395_c0_g2 | CcERF47 | AP2 domain | 19.89 | 63.02 | 50.70 |
| TRINITY_DN38620_c0_g2 | CcERF48 | AP2 domain | 1.62 | 24.52 | 19.87 |
| TRINITY_DN42028_c0_g1 | CcERF49 | AP2 domain | 560.51 | 195.60 | 181.98 |
| TRINITY_DN46449_c2_g7 | CcERF50 | AP2 domain | 1.34 | 5.89 | 21.54 |
| TRINITY_DN45591_c1_g2 | CcERF51 | AP2 domain | 4.95 | 54.98 | 19.17 |
| TRINITY_DN44628_c0_g2 | CcERF52 | AP2 domain | 34.09 | 281.37 | 108.66 |
| TRINITY_DN48643_c0_g3 | CcERF53 | AP2 domain | 12.35 | 12.98 | 103.01 |
| TRINITY_DN48918_c0_g9 | CcERF54 | AP2 domain | 0.21 | 12.92 | 14.29 |
| TRINITY_DN44939_c1_g6 | CcERF55 | AP2 domain | 0.12 | 165.15 | 99.57 |
| TRINITY_DN31476_c0_g1 | CcERF56 | AP2 domain | 1.10 | 25.07 | 65.46 |
| TRINITY_DN43789_c0_g3 | CcERF57 | AP2 domain | 2.39 | 62.54 | 79.03 |
| TRINITY_DN41307_c0_g1 | CcERF58 | AP2 domain | 42.36 | 17.35 | 15.91 |
| TRINITY_DN48573_c0_g1 | CcERF59 | AP2 domain | 12.46 | 4.66 | 36.68 |
| TRINITY_DN48667_c1_g1 | CcERF60 | AP2 domain | 0.04 | 2.52 | 0.32 |
| TRINITY_DN44847_c0_g2 | CcERF61 | AP2 domain | 0.19 | 615.10 | 179.84 |
| TRINITY_DN25477_c0_g1 | CcERF62 | AP2 domain | 4.52 | 15.66 | 5.25 |
| TRINITY_DN47856_c0_g2 | CcERF63 | AP2 domain | 0.07 | 139.73 | 19.60 |
| TRINITY_DN50063_c1_g3 | CcERF64 | AP2 domain | 0.22 | 28.04 | 12.52 |
| TRINITY_DN44171_c0_g1 | CcERF65 | AP2 domain | 7.74 | 148.83 | 51.18 |
| TRINITY_DN44171_c1_g1 | CcERF66 | AP2 domain | 3.49 | 7.57 | 3.86 |
| TRINITY_DN44171_c1_g2 | CcERF67 | AP2 domain | 4.08 | 4.66 | 10.16 |
| TRINITY_DN50995_c1_g1 | CcERF68 | AP2 domain | 135.94 | 92.68 | 74.03 |
| TRINITY_DN50995_c1_g4 | CcERF69 | AP2 domain | 9.57 | 2.50 | 4.36 |
| TRINITY_DN55146_c0_g1 | CcERF70 | AP2 domain | 2.96 | 2.96 | 1.47 |
| TRINITY_DN50712_c0_g3 | CcERF71 | AP2 domain | 40.56 | 9.49 | 2.70 |
| TRINITY_DN41575_c0_g1 | CcERF72 | AP2 domain | 1.76 | 5.19 | 1.90 |
| TRINITY_DN45331_c1_g5 | CcERF73 | AP2 domain | 16.44 | 173.99 | 90.00 |
| TRINITY_DN44586_c0_g3 | CcERF74 | AP2 domain | 0.12 | 44.80 | 17.00 |
| TRINITY_DN44586_c0_g8 | CcERF75 | AP2 domain | 2.60 | 70.86 | 7.35 |
| TRINITY_DN44586_c0_g7 | CcERF76 | AP2 domain | 0.16 | 60.05 | 6.61 |
| TRINITY_DN44586_c0_g9 | CcERF77 | AP2 domain | 7.26 | 3.28 | 7.06 |
| TRINITY_DN44586_c0_g4 | CcERF78 | AP2 domain | 7.65 | 6.34 | 3.09 |
| TRINITY_DN44586_c0_g10 | CcERF79 | AP2 domain | 1.42 | 2.89 | 15.66 |
| TRINITY_DN43260_c3_g5 | CcERF80 | AP2 domain | 0.00 | 0.40 | 2.03 |
| TRINITY_DN44038_c0_g1 | CcBHLH1 | Helix-loop-helix DNA-binding domain | 0.24 | 0.19 | 1.75 |
| TRINITY_DN38527_c0_g1 | CcBHLH2 | Helix-loop-helix DNA-binding domain | 0.44 | 0.07 | 0.94 |
| TRINITY_DN19706_c0_g1 | CcBHLH3 | Helix-loop-helix DNA-binding domain | 0.02 | 0.39 | 0.63 |
| TRINITY_DN42693_c1_g1 | CcBHLH4 | Helix-loop-helix DNA-binding domain | 0.11 | 0.71 | 0.55 |
| TRINITY_DN36828_c0_g2 | CcBHLH5 | Helix-loop-helix DNA-binding domain | 1.05 | 0.25 | 0.11 |
| TRINITY_DN41060_c0_g1 | CcBHLH6 | Helix-loop-helix DNA-binding domain | 0.38 | 3.40 | 1.45 |
| TRINITY_DN45351_c1_g3 | CcBHLH7 | Helix-loop-helix DNA-binding domain | 2.15 | 0.90 | 1.23 |
| TRINITY_DN45351_c1_g1 | CcBHLH8 | Helix-loop-helix DNA-binding domain | 0.00 | 0.67 | 1.14 |
| TRINITY_DN45351_c1_g4 | CcBHLH9 | Helix-loop-helix DNA-binding domain | 3.27 | 0.97 | 14.86 |
| TRINITY_DN45368_c0_g1 | CcBHLH10 | Helix-loop-helix DNA-binding domain | 0.02 | 0.89 | 1.06 |
| TRINITY_DN49163_c2_g1 | CcBHLH11 | Helix-loop-helix DNA-binding domain | 16.75 | 7.33 | 3.99 |
| TRINITY_DN49345_c0_g1 | CcBHLH12 | Helix-loop-helix DNA-binding domain | 0.00 | 0.74 | 0.71 |
| TRINITY_DN41404_c0_g1 | CcBHLH13 | Helix-loop-helix DNA-binding domain | 0.00 | 1.42 | 0.82 |
| TRINITY_DN43268_c0_g7 | CcBHLH14 | Helix-loop-helix DNA-binding domain | 20.67 | 15.35 | 38.33 |
| TRINITY_DN44271_c2_g1 | CcBHLH15 | Helix-loop-helix DNA-binding domain | 41.29 | 17.56 | 22.85 |
| TRINITY_DN45349_c1_g1 | CcBHLH16 | Helix-loop-helix DNA-binding domain | 0.00 | 2.85 | 3.41 |
| TRINITY_DN48629_c0_g5 | CcBHLH17 | Helix-loop-helix DNA-binding domain | 0.09 | 2.52 | 0.75 |
| TRINITY_DN43084_c0_g5 | CcBHLH18 | Helix-loop-helix DNA-binding domain | 98.62 | 73.83 | 58.03 |
| TRINITY_DN31133_c0_g2 | CcBHLH19 | Helix-loop-helix DNA-binding domain | 5.93 | 3.28 | 2.89 |
| TRINITY_DN46163_c5_g1 | CcBHLH20 | Helix-loop-helix DNA-binding domain | 0.55 | 2.59 | 0.92 |
| TRINITY_DN46019_c0_g1 | CcBHLH21 | Helix-loop-helix DNA-binding domain | 4.72 | 3.48 | 2.10 |
| TRINITY_DN39922_c0_g1 | CcBHLH22 | Helix-loop-helix DNA-binding domain | 6.60 | 1.62 | 8.71 |
| TRINITY_DN49805_c0_g1 | CcBHLH23 | Helix-loop-helix DNA-binding domain | 21.69 | 61.14 | 54.84 |
| TRINITY_DN43530_c0_g1 | CcBHLH24 | Helix-loop-helix DNA-binding domain | 1.59 | 22.60 | 8.55 |
| TRINITY_DN49815_c1_g8 | CcBHLH25 | Helix-loop-helix DNA-binding domain | 20.55 | 20.82 | 11.62 |
| TRINITY_DN51231_c0_g2 | CcBHLH26 | Helix-loop-helix DNA-binding domain | 6.38 | 12.76 | 7.00 |
| TRINITY_DN48847_c1_g1 | CcBHLH27 | Helix-loop-helix DNA-binding domain | 0.01 | 1.21 | 4.31 |
| TRINITY_DN48847_c1_g5 | CcBHLH28 | Helix-loop-helix DNA-binding domain | 0.38 | 6.13 | 9.51 |
| TRINITY_DN47384_c0_g3 | CcBHLH29 | Helix-loop-helix DNA-binding domain | 0.16 | 0.73 | 0.12 |
| TRINITY_DN46790_c2_g1 | CcBHLH30 | Helix-loop-helix DNA-binding domain | 4.53 | 3.29 | 2.10 |
| TRINITY_DN48296_c2_g1 | CcBHLH31 | Helix-loop-helix DNA-binding domain | 12.76 | 7.21 | 7.70 |
| TRINITY_DN48270_c2_g3 | CcBHLH32 | Helix-loop-helix DNA-binding domain | 9.71 | 29.62 | 12.76 |
| TRINITY_DN28067_c0_g1 | CcBHLH33 | Helix-loop-helix DNA-binding domain | 5.48 | 108.39 | 48.68 |
| TRINITY_DN30426_c0_g3 | CcBHLH34 | Helix-loop-helix DNA-binding domain | 6.77 | 1006.33 | 97.68 |
| TRINITY_DN47927_c0_g1 | CcBHLH35 | Helix-loop-helix DNA-binding domain | 15.55 | 6.11 | 6.51 |
| TRINITY_DN43370_c0_g1 | CcBHLH36 | Helix-loop-helix DNA-binding domain | 69.16 | 188.74 | 152.68 |
| TRINITY_DN45803_c1_g6 | CcBHLH37 | Helix-loop-helix DNA-binding domain | 2.81 | 48.67 | 15.50 |
| TRINITY_DN45803_c1_g1 | CcBHLH38 | Helix-loop-helix DNA-binding domain | 14.20 | 103.91 | 43.93 |
| TRINITY_DN37799_c0_g2 | CcBHLH39 | Helix-loop-helix DNA-binding domain | 25.40 | 10.73 | 4.33 |
| TRINITY_DN34917_c0_g1 | CcBHLH40 | Helix-loop-helix DNA-binding domain | 8.32 | 1.64 | 14.28 |
| TRINITY_DN37053_c0_g1 | CcBHLH41 | Helix-loop-helix DNA-binding domain | 23.18 | 211.67 | 36.75 |
| TRINITY_DN28254_c0_g2 | CcBHLH42 | Helix-loop-helix DNA-binding domain | 0.41 | 83.36 | 12.08 |
| TRINITY_DN42728_c0_g1 | CcBHLH43 | Helix-loop-helix DNA-binding domain | 254.55 | 149.46 | 95.77 |
| TRINITY_DN44014_c0_g1 | CcBHLH44 | Helix-loop-helix DNA-binding domain | 5.43 | 3.33 | 1.49 |
| TRINITY_DN45579_c0_g3 | CcBHLH45 | Helix-loop-helix DNA-binding domain | 1.02 | 243.99 | 157.07 |
| TRINITY_DN46569_c0_g1 | CcBHLH46 | Helix-loop-helix DNA-binding domain | 0.34 | 0.24 | 1.08 |
| TRINITY_DN48331_c2_g5 | CcBHLH47 | Helix-loop-helix DNA-binding domain | 24.22 | 104.46 | 42.15 |
| TRINITY_DN43800_c0_g1 | CcBHLH48 | Helix-loop-helix DNA-binding domain | 0.82 | 52.56 | 6.68 |
| TRINITY_DN47882_c0_g1 | CcBHLH49 | Helix-loop-helix DNA-binding domain | 97.62 | 59.02 | 20.03 |
| TRINITY_DN49353_c1_g4 | CcBHLH50 | Helix-loop-helix DNA-binding domain | 2.64 | 10.56 | 3.99 |
| TRINITY_DN45412_c2_g4 | CcBHLH51 | Helix-loop-helix DNA-binding domain | 17.51 | 15.62 | 7.46 |
| TRINITY_DN50724_c2_g2 | CcBHLH52 | Helix-loop-helix DNA-binding domain | 5.23 | 8.01 | 3.97 |
| TRINITY_DN41360_c0_g1 | CcBHLH53 | Helix-loop-helix DNA-binding domain | 1.02 | 0.78 | 4.90 |
| TRINITY_DN45589_c1_g3 | CcBHLH54 | Helix-loop-helix DNA-binding domain | 22.97 | 25.08 | 7.88 |
| TRINITY_DN35569_c0_g1 | CcBHLH55 | Helix-loop-helix DNA-binding domain | 3.41 | 104.09 | 10.62 |
