# Supplementary material for: Metabolism and transcriptome profiling provides insight into the genes and transcription factors involved in monoterpene biosynthesis of borneol chemotype of Cinnamomum camphora induced by mechanical damage
Source: PeerJ. 2021 Jul 1;9:e11465. doi: 10.7717/peerj.11465 (PMC8255067; doi:10.7717/peerj.11465)
Supplement: Supplemental Information 15 [file peerj-09-11465-s015.docx]

| **CcTFs** | **CcTPSs** | **Correlation coefficient** | **P_ value** |
| --- | --- | --- | --- |
| CcBHLH42 | CcTPS1 | 0.823529 | 0.031462 |
| CcBHLH8 | CcTPS1 | 0.848739 | 0.021264 |
| CcBHLH7 | CcTPS1 | 0.794986 | 0.04526 |
| CcWRKY20 | CcTPS1 | 0.840336 | 0.022994 |
| CcWRKY35 | CcTPS1 | 0.811723 | 0.033522 |
| CcWRKY2 | CcTPS1 | 0.840336 | 0.022994 |
| CcWRKY19 | CcTPS1 | 0.878669 | 0.011786 |
| CcWRKY2 | CcTPS4 | 0.803354 | 0.038385 |
| CcWRKY13 | CcTPS4 | 0.883333 | 0.010787 |
| CcWRKY19 | CcTPS4 | 0.9 | 0.007283 |
| CcERF31 | CcTPS1 | 0.791004 | 0.03332 |
| CcERF73 | CcTPS1 | 0.811723 | 0.024756 |
| CcERF5 | CcTPS4 | 0.95 | 0.001497 |
| CcERF7 | CcTPS4 | 0.916667 | 0.003522 |
| CcERF11 | CcTPS4 | 0.916667 | 0.003522 |
| CcERF34 | CcTPS4 | 0.766667 | 0.044833 |
| CcERF35 | CcTPS4 | 0.833333 | 0.017618 |
| CcERF42 | CcTPS4 | 0.85 | 0.013275 |
| CcERF63 | CcTPS4 | 0.85 | 0.013275 |
| CcERF18 | CcTPS4 | 0.803354 | 0.028382 |
| CcERF47 | CcTPS4 | 0.928878 | 0.002737 |
| CcERF70 | CcTPS4 | 0.864531 | 0.010078 |
| CcMYB88 | CcTPS1 | 0.861932 | 0.018205 |
| CcMYB111 | CcTPS1 | 0.848739 | 0.021939 |
| CcMYB125 | CcTPS1 | 0.845196 | 0.023394 |
| CcMYB56 | CcTPS1 | 0.861932 | 0.018205 |
| CcMYB67 | CcTPS1 | 0.811723 | 0.036801 |
| CcMYB75 | CcTPS1 | 0.828459 | 0.029627 |
| CcMYB101 | CcTPS1 | 0.861932 | 0.018205 |
| CcMYB125 | CcTPS4 | 0.916667 | 0.007011 |
| CcMYB56 | CcTPS4 | 0.833333 | 0.027443 |
| CcMYB67 | CcTPS4 | 0.883333 | 0.013087 |
| CcMYB84 | CcTPS4 | 0.816667 | 0.034209 |
| CcMYB75 | CcTPS4 | 0.883333 | 0.013087 |
| CcMYB101 | CcTPS4 | 0.866667 | 0.016714 |
| CcMYB102 | CcTPS4 | 0.883333 | 0.013087 |
| CcMYB103 | CcTPS4 | 0.811723 | 0.036801 |
| CcMYB123 | CcTPS4 | 0.966667 | 0.00107 |
| CcMYB124 | CcTPS4 | 0.933333 | 0.004709 |
| CcMYB91 | CcTPS4 | 0.85 | 0.02136 |
| CcMYB71 | CcTPS4 | 0.916667 | 0.007011 |
| CcMYB54 | CcTPS4 | 0.933333 | 0.004709 |
| CcMYB4 | CcTPS4 | 0.928878 | 0.005517 |
| CcbZIP2 | CcTPS1 | 0.844538 | 0.029876 |
| CcbZIP7 | CcTPS1 | 0.820091 | 0.042852 |
| CcbZIP8 | CcTPS1 | 0.895405 | 0.013482 |
| CcbZIP26 | CcTPS4 | 0.95 | 0.002499 |
| CcbZIP18 | CcTPS4 | 0.85 | 0.02689 |
| CcbZIP29 | CcTPS4 | 0.816667 | 0.042852 |
| CcbZIP7 | CcTPS4 | 0.866667 | 0.022261 |
