# Supplementary material for: Metabolism and transcriptome profiling provides insight into the genes and transcription factors involved in monoterpene biosynthesis of borneol chemotype of Cinnamomum camphora induced by mechanical damage
Source: PeerJ. 2021 Jul 1;9:e11465. doi: 10.7717/peerj.11465 (PMC8255067; doi:10.7717/peerj.11465)
Supplement: Supplemental Information 16 [file peerj-09-11465-s016.docx]

| **Gene ID** | **Pathway_definition** | **KO_name** | **FPKM** | | |
| --- | --- | --- | --- | --- | --- |
|  |  |  | **CK** | **MD_2h** | **MD_6h** |
| TRINITY_DN46358_c3_g4 | alpha-Linolenic acid metabolism | PLA2G, SPLA2 | 0.696667 | 0.37 | 10.763333 |
| TRINITY_DN45893_c1_g5 | alpha-Linolenic acid metabolism | DAD1 | 0.013333 | 2.656667 | 6.646667 |
| TRINITY_DN43794_c1_g1 | alpha-Linolenic acid metabolism | LOX2S | 0.39 | 1.26 | 0.696667 |
| TRINITY_DN51091_c0_g1 | alpha-Linolenic acid metabolism | LOX2S | 33.706667 | 902.73667 | 257.96 |
| TRINITY_DN41294_c0_g1 | alpha-Linolenic acid metabolism | LOX2S | 653.96 | 972.31333 | 793.4 |
| TRINITY_DN47634_c0_g1 | alpha-Linolenic acid metabolism | AOS | 1.413333 | 42.856667 | 67.39 |
| TRINITY_DN51210_c1_g2 | alpha-Linolenic acid metabolism | AOC | 15.513333 | 96.053333 | 285.90667 |
| TRINITY_DN51210_c1_g1 | alpha-Linolenic acid metabolism | AOC | 4.91 | 61.173333 | 125.31667 |
| TRINITY_DN50872_c0_g1 | alpha-Linolenic acid metabolism | OPR | 2.763333 | 3.463333 | 7.446667 |
| TRINITY_DN48700_c1_g2 | alpha-Linolenic acid metabolism | OPR | 0.223333 | 5.636667 | 1.38 |
| TRINITY_DN50872_c0_g2 | alpha-Linolenic acid metabolism | OPR | 0 | 3.71 | 0.396667 |
| TRINITY_DN44970_c5_g1 | alpha-Linolenic acid metabolism | OPR | 5.333333 | 25.493333 | 44.223333 |
| TRINITY_DN43748_c3_g2 | alpha-Linolenic acid metabolism | OPR | 21.67 | 11.526667 | 36.15 |
| TRINITY_DN47828_c0_g3 | alpha-Linolenic acid metabolism | OPR | 2.16 | 1.276667 | 56.186667 |
| TRINITY_DN47828_c0_g1 | alpha-Linolenic acid metabolism | OPR | 0.386667 | 4.946667 | 27.703333 |
| TRINITY_DN51325_c4_g4 | alpha-Linolenic acid metabolism | OPR | 18.133333 | 10.546667 | 28.58 |
| TRINITY_DN43748_c3_g1 | alpha-Linolenic acid metabolism | OPR | 30.546667 | 439.25 | 378.95333 |
| TRINITY_DN47495_c3_g5 | alpha-Linolenic acid metabolism | OPCL1 | 0.136667 | 9.813333 | 1.06 |
| TRINITY_DN50804_c2_g2 | alpha-Linolenic acid metabolism | OPCL1 | 33.283333 | 536.91333 | 141.22667 |
| TRINITY_DN47495_c3_g8 | alpha-Linolenic acid metabolism | OPCL1 | 29.376667 | 409.89333 | 146.16333 |
| TRINITY_DN51016_c0_g6 | alpha-Linolenic acid metabolism | OPCL1 | 1.383333 | 2.673333 | 0.903333 |
| TRINITY_DN49017_c1_g5 | alpha-Linolenic acid metabolism | MFP2 | 41.093333 | 56.893333 | 124.99667 |
| TRINITY_DN46183_c0_g6 | alpha-Linolenic acid metabolism | ACAA1 | 1.14 | 56.956667 | 26.466667 |
| TRINITY_DN46919_c1_g8 | alpha-Linolenic acid metabolism | ACAA1 | 0.086667 | 4.276667 | 4.07 |
| TRINITY_DN46919_c0_g2 | alpha-Linolenic acid metabolism | ACAA1 | 0 | 0.46 | 1.983333 |
| TRINITY_DN45702_c0_g1 | alpha-Linolenic acid metabolism | ACAA1 | 0.146667 | 0.186667 | 1.076667 |
| TRINITY_DN46919_c1_g4 | alpha-Linolenic acid metabolism | ACAA1 | 0 | 0.296667 | 2.42 |
| TRINITY_DN46183_c0_g1 | alpha-Linolenic acid metabolism | ACAA1 | 0.123333 | 3.796667 | 4.063333 |
| TRINITY_DN46919_c1_g15 | alpha-Linolenic acid metabolism | ACAA1 | 0.53 | 1.073333 | 0 |
| TRINITY_DN46358_c3_g3 | alpha-Linolenic acid metabolism | JMT | 19.396667 | 143.23333 | 975.23667 |
| TRINITY_DN44551_c3_g3 | alpha-Linolenic acid metabolism | JMT | 0.166667 | 18.003333 | 13.366667 |
| TRINITY_DN40210_c0_g1 | alpha-Linolenic acid metabolism | JMT | 0.61 | 1.83 | 0.143333 |
| TRINITY_DN49992_c0_g3 | alpha-Linolenic acid metabolism | JMT | 11.86 | 4.03 | 2.993333 |
| TRINITY_DN42088_c0_g2 | JA-signaling pathway | JAR | 6.046667 | 2.74 | 10.33 |
| TRINITY_DN47723_c0_g7 | JA-signaling pathway | JAR | 83.726667 | 754.61667 | 128.24333 |
| TRINITY_DN50977_c1_g2 | JA-signaling pathway | JAR | 144.60667 | 970.25333 | 213.06333 |
| TRINITY_DN45678_c2_g1 | JA-signaling pathway | JAZ | 4.456667 | 1712.4267 | 519.81333 |
| TRINITY_DN47146_c1_g3 | JA-signaling pathway | JAZ | 2.926667 | 531.6 | 110.32667 |
| TRINITY_DN48887_c1_g1 | JA-signaling pathway | JAZ | 17.433333 | 700.92333 | 189.36667 |
| TRINITY_DN50312_c0_g4 | JA-signaling pathway | JAZ | 13.163333 | 60.753333 | 17.06 |
| TRINITY_DN47882_c0_g1 | JA-signaling pathway | MYC2 | 23.18 | 211.67333 | 36.753333 |
| TRINITY_DN48847_c1_g1 | JA-signaling pathway | MYC3 | 24.22 | 104.46333 | 42.146667 |
| TRINITY_DN48847_c1_g5 | JA-signaling pathway | MYC4 | 0.823333 | 52.563333 | 6.676667 |
