# Supplementary figures and images for: Metabolism and transcriptome profiling provides insight into the genes and transcription factors involved in monoterpene biosynthesis of borneol chemotype of Cinnamomum camphora induced by mechanical damage
Source: PeerJ. 2021 Jul 1;9:e11465. doi: 10.7717/peerj.11465 (PMC8255067; doi:10.7717/peerj.11465)

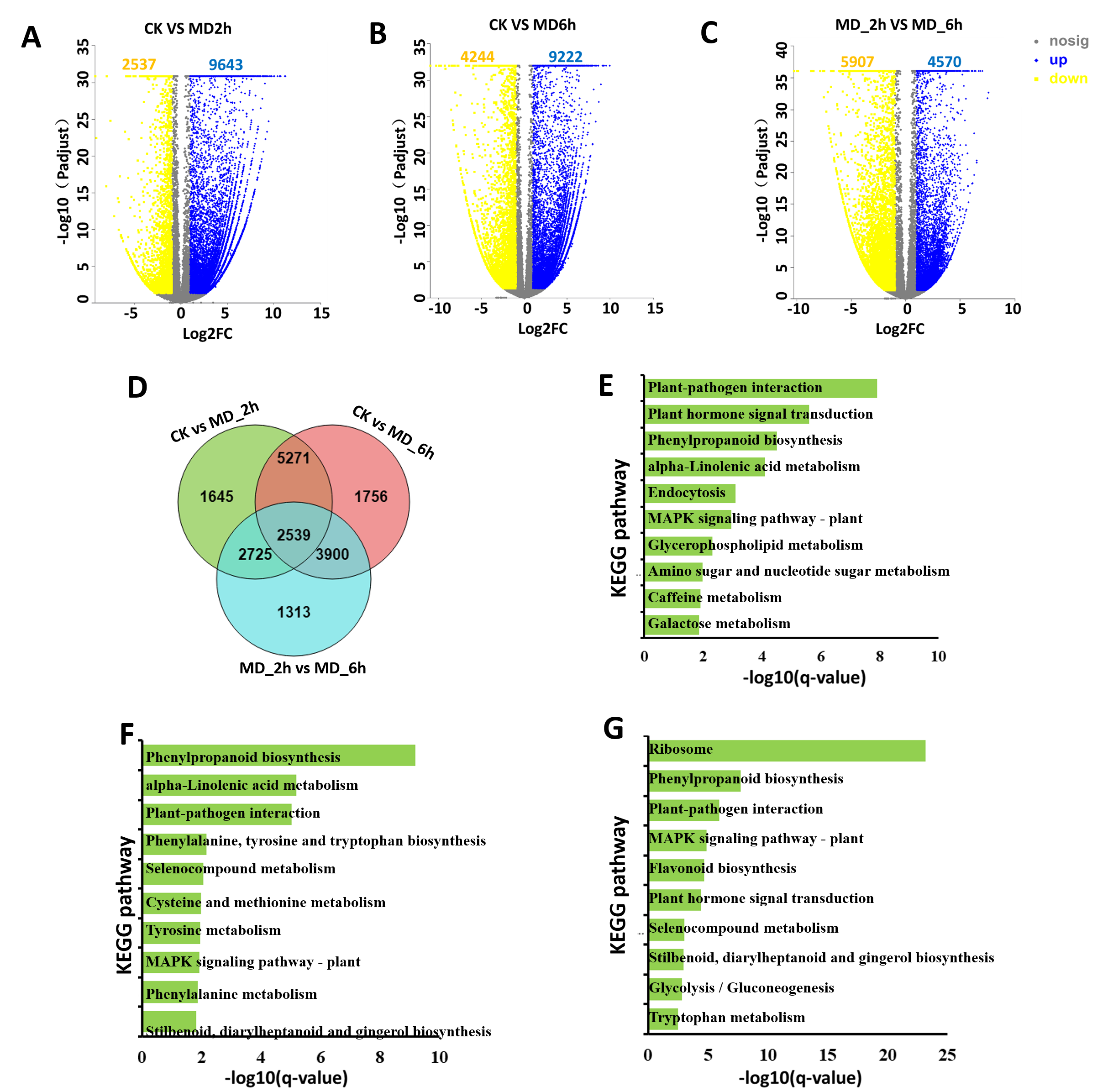

Supplement: Supplemental Information 18 — A Volcano map showed the numbers of up- and down-regulation of DEGs between CK and MD_2h; B Volcano map showed the numbers of up- and down-regulation of DEGs between CK and MD_6h; C Volcano map showed the numbers of up- and down-regulation of DEGs between MD_2h and MD_6h; D A venn diagram showed the numbers of common and specific DEGs in three comparisons; E KEGG enrichment analysis of DEGs between CK and MD_2h; F KEGG enrichment analysis of DEGs between CK and MD_6h; G KEGG enrichment analysis of DEGs between MD_2h and MD_6h. [file peerj-09-11465-s018.png]

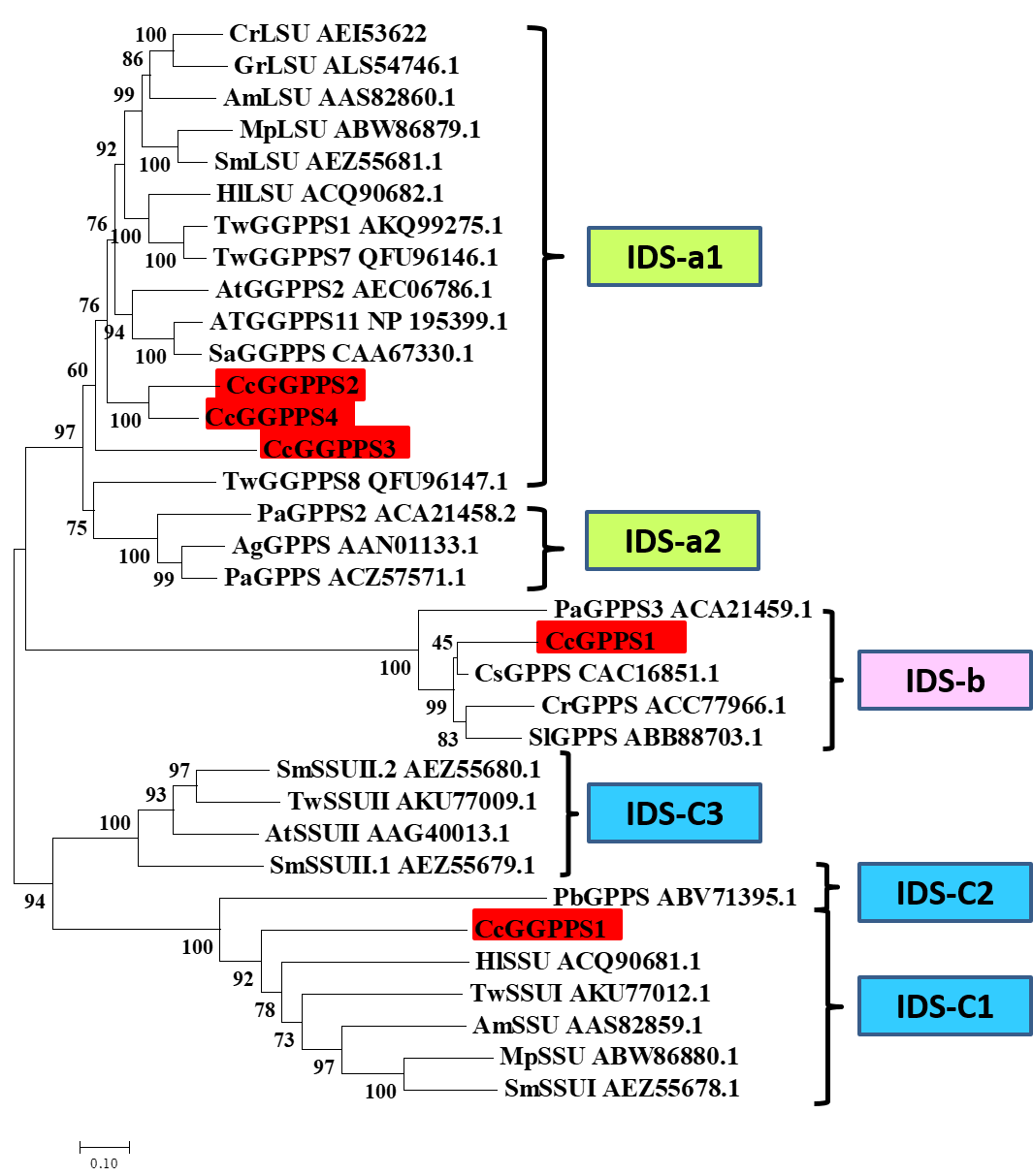

Supplement: Supplemental Information 19 — GPPS/GGPPS identified in this study were shown in red square. [file peerj-09-11465-s019.png]

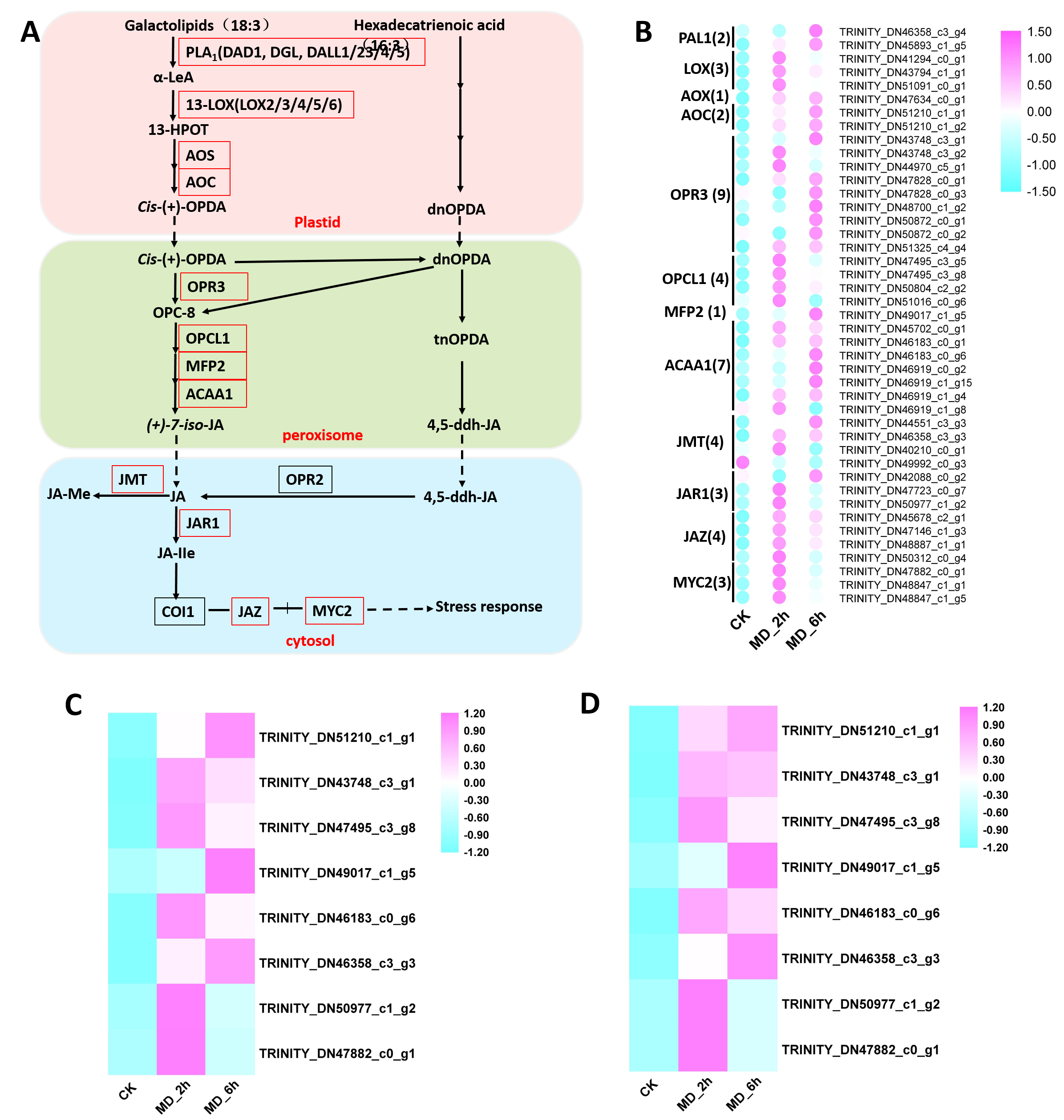

Supplement: Supplemental Information 20 — A Enzymes involved in biosynthesis and catabolism of JA; Enzymes identified in this study were shown in red frames, otherwise black frame. Enzymes with similar functions are listed in brackets. PLA1, phospholipase A1;DAD1, delayed anther dehiscence1; DGL and DALL, DAD1-LIKE lipase; LOX, lipoxygenase; AOS, allene oxide synthase; AOC, allene oxide cyclase; OPR3, OPDA reductase3; JMT, jasmonic acid carboxyl methyl transferase; OPR2, OPDA reductase2; JAR1, jasmonic resistant 1 (a JA amino acid conjugate synthase); cis- (+) -OPDA, cis- (+) -12-oxo-phytodienoic acid; OPC-8, 3-oxo-2- (2-pentenyl) -cyclopentane-1-octanoic acid; JA-Me, JA methyl ester; dnOPDA, dinor-OPDA; tnOPDA, tetranor-OPDA; 4,5-ddh-JA, 4,5-didehydro-JA; COI1: Coronatine-insensitive protein 1; JAZ: Jasmonate ZIM domain-containing protein; MYC2: Myelocytomatosis proteins; B Expression analysis of the unigenes related to the JA biosynthesis and JA signaling pathway; C qRT-PCR results of the 8 selected genes; D FPKM results of the 8 selected genes. [file peerj-09-11465-s020.png]
